# Supplementary material for: Coverage of antenatal, intrapartum, and newborn care in 104 districts of Ethiopia: A before and after study four years after the launch of the national Community-Based Newborn Care programme
Source: PLoS One. 2021 Aug 5;16(8):e0251706. doi: 10.1371/journal.pone.0251706 (PMC8341496; doi:10.1371/journal.pone.0251706)
Supplement: S1 File — (PDF) [file pone.0251706.s001.pdf]

COMMUNITY BASED NEWBORN CARE IN ETHIOPIA

HOUSEHOLD SURVEY QUESTIONNAIRE V 2.1

Module 1

**SECTION 1: Household Identifiers and Characteristics**			
100	Date (dd/mm/yyyy)	|__|__| /|__|__|/ |__|__|__|__|	
101	Region	|__|__|__|__|__|__|__|__|__|__|__|__|__|__|__|	
102	Zone	|__|__|__|__|__|__|__|__|__|__|__|__|__|__|__|	
103	Woreda name	|__|__|__|__|__|__|__|__|__|__|__|__|__|__|__|	
104	PHCU/Health Center name	|__|__|__|__|__|__|__|__|__|__|__|__|__|__|__|	
105	Kebele name	|__|__|__|__|__|__|__|__|__|__|__|__|__|__|__|	
106	Gote name	|__|__|__|__|__|__|__|__|__|__|__|__|__|__|__|	
107	PHCU Cluster Number<br>***Enter the cluster number***	|__|__|__|	
108	Household number<br>***Enter the household number***	|__|__|	
109	Unique household ID<br>***To be copied onto all documents, e.g. consent forms***	|__|__|__| / |__|__| cluster household	
110	GPS Latitude	|__|__| : |__|__|__|__|__|	
111	GPS Longitude	|__|__| : |__|__|__|__|__|	
112	Name of household head	|__|__|__|__|__|__|__|__|__|__|__|__|__|__|__|	
113	Date of first visit	Day |___|___| Month |___|___| Year |___|___|___|___|	
114	Interviewer's name	Name ________________________________	
115	***Interviewer: Have you read him/her the consent form?***	**1 = Yes**<br>**2 = No**	|__|

116	***Interviewer: Does the respondent agree?***	**1 = Yes**<br>**2 = No- *end the interview here and move on to the next household.***	**|__|**
117	What is the ethnic group of the household head?	**1 = Agew**<br>**2 = Amhara**<br>**3 = Bench**<br>**4 = Burji**<br>**5 = Dizi**<br>**6 = Gedeo**<br>**7 = Gurage**<br>**8 = Hadiya**<br>**9 = Keficho**<br>**10= Kembata**<br>**11= Konta**<br>**12 = Me'enite**<br>**13 = Oromo**<br>**14 = Silite**<br>**15 = Tigray**<br>**16=Welayita**<br>**17 = Other Ethiopian National Groups**<br>**18 = From two or more ethnic groups**	**|__|__|**

Visits		**1st**	**2nd**	**3rd**	**Final Visit**
Date (dd/mm/yyyy)		|__|__| / |__|__| / |__|__|__|__|	|__|__| / |__|__| / |__|__|__|__|	|__|__| / |__|__| / |__|__|__|__|	**Day |__|__|**<br>**Month |__|__|**<br>**Year |__|__|__|__|**<br>**Result |__|**
Interviewer's name					
Result (Enter relevant code below)		|__|	|__|	|__|	
If not completed during this interview, when is the next visit (appointment)	***Date***(dd/mm/yyyy)		|__|__| / |__|__| / |__|__|__|__|	|__|__| / |__|__| / |__|__|__|__|	
	Time				

Result Codes:
1. Completed
2. Partly completed
3. Postponed
4. Not at home
5. No-one competent to respond
6. Refused

If refused end interview.

<table>
<tr><td>118</td><td colspan="9">Household Listing
Now I would like to ask you about people who are residents of this house. By resident, I mean everyone who has been present for the last 3 months and who normally eat together.
Start with head of the household and older people, then children from oldest to youngest.</td></tr>
<tr><th rowspan="2">Person Number</th><th>Person Name</th><th>Sex</th><th>Date of birth</th><th>Age</th><th>Eligible? (woman aged 13-49)</th><th>Marital status</th><th>Formal years of education completed</th><th>Religion</th></tr>
<tr><td>Enter First Name (If Identical names in the same household use the initial of the last name)</td><td>1 = Male
2 = Female</td><td>(dd/mm/yyyy)
Enter 99 if don’t know dd
Enter 99 if don’t know mm
Enter 9999 if don’t know yyyy</td><td>Enter age in completed years

If person is less than one year, enter 0</td><td>1 = Yes
2 = No</td><td>1 = Currently married
2 = In a union
3 = Never married
4 = Divorced
5 = Widowed
6 = Not Applicable</td><td>Enter number of formal years of education completed</td><td>1 = Orthodox
2 = Catholic
3 = Protestant
4 = Muslim
5 = Other
6 = Not Applicable</td></tr>
<tr><td>1</td><td>|__|__|__|__|__|__|__|__|__|__|__|__|__|_</td><td>|__|</td><td>|__|__|/|__|__|/|__|__|__|__|</td><td>|__|__|</td><td>|__|</td><td>|__|</td><td>|__|__|</td><td>|__|</td></tr>
<tr><td>2</td><td>|__|__|__|__|__|__|__|__|__|__|__|__|__|</td><td>|__|</td><td>|__|__|/|__|__|/|__|__|__|__|</td><td>|__|__|</td><td>|__|</td><td>|__|</td><td>|__|__|</td><td>|__|</td></tr>
<tr><td>3</td><td>|__|__|__|__|__|__|__|__|__|__|__|__|__|</td><td>|__|</td><td>|__|__|/|__|__|/|__|__|__|__|</td><td>|__|__|</td><td>|__|</td><td>|__|</td><td>|__|__|</td><td>|__|</td></tr>
<tr><td>4</td><td>|__|__|__|__|__|__|__|__|__|__|__|__|__|</td><td>|__|</td><td>|__|__|/|__|__|/|__|__|__|__|</td><td>|__|__|</td><td>|__|</td><td>|__|</td><td>|__|__|</td><td>|__|</td></tr>
<tr><td>5</td><td>|__|__|__|__|__|__|__|__|__|__|__|__|__|</td><td>|__|</td><td>|__|__|/|__|__|/|__|__|__|__|</td><td>|__|__|</td><td>|__|</td><td>|__|</td><td>|__|__|</td><td>|__|</td></tr>
<tr><td>6</td><td>|__|__|__|__|__|__|__|__|__|__|__|__|__|</td><td>|__|</td><td>|__|__|/|__|__|/|__|__|__|__|</td><td>|__|__|</td><td>|__|</td><td>|__|</td><td>|__|__|</td><td>|__|</td></tr>
<tr><td>7</td><td>|__|__|__|__|__|__|__|__|__|__|__|__|__|</td><td>|__|</td><td>|__|__|/|__|__|/|__|__|__|__|</td><td>|__|__|</td><td>|__|</td><td>|__|</td><td>|__|__|</td><td>|__|</td></tr>
<tr><td>8</td><td>|__|__|__|__|__|__|__|__|__|__|__|__|__|</td><td>|__|</td><td>|__|__|/|__|__|/|__|__|__|__|</td><td>|__|__|</td><td>|__|</td><td>|__|</td><td>|__|__|</td><td>|__|</td></tr>
<tr><td>9</td><td>|__|__|__|__|__|__|__|__|__|__|__|__|__|</td><td>|__|</td><td>|__|__|/|__|__|/|__|__|__|__|</td><td>|__|__|</td><td>|__|</td><td>|__|</td><td>|__|__|</td><td>|__|</td></tr>
<tr><td>10</td><td>|__|__|__|__|__|__|__|__|__|__|__|__|__|</td><td>|__|</td><td>|__|__|/|__|__|/|__|__|__|__|</td><td>|__|__|</td><td>|__|</td><td>|__|</td><td>|__|__|</td><td>|__|</td></tr>
</table>

Now I want to ask you some questions about the characteristics of your household

Interviewer: In this section and throughout the questionnaire, please read out all the options to the interviewee UNLESS it is clearly stated 'Do not read list'.

119	What is the main material of the walls?	**1 = No walls<br>2 = Natural materials (cane, wood, mud, straw)<br>3 = Stone with mud<br>4 = Stone/bricks with cement<br>5 = Other**	**|__|**
120	What is the main floor material?	**1 = Natural floor (earth/sand/dung)<br>2 = Rudimentary floor (wood/palm/bamboo)<br>3 = Finished floor (polished wood/ vinyl/tiles/cement/carpet)<br>4 = Other**	**|__|**
121	What is the main material of the roof?	**1 = Thatch/grass or leaves<br>2 = Iron sheets or tiles<br>3 = Other**	**|__|**
122	What kind of toilet facilities does your household have?	**1 = No facility/bush/field<br>2 = Pit toilet/latrine<br>3 = Flush toilet**	**|__|**
123	What is the main source of drinking water for the household?<br><br>**Do not read list**	**1 = Piped connection into house<br>2 = Piped connection into yard<br>3 = Public standpipes<br>4 = Boreholes<br>5 = Protected dug wells<br>6 = Protected springs<br>7 = Rainwater collection<br>8 = Surface water<br>9 = Open dug wells<br>10 = Unprotected springs<br>11 = Vendor provided water<br>12 = Bottled water<br>13 = Tanker**	**|__||__|**
124	Do you do anything to the water to make it safer to drink?	**1 = Yes<br>2 = No-GO to 127<br>3 = Don't know --GO to 127**	**|__|**
125	If yes, what is the main thing you do?<br><br>**Do not read list**	**1 = Let it stand and settle<br>2 = Strain through a cloth<br>3 = Use water filter (ceramic/sand/composite/etc)<br>4 = Boil<br>5 = Solar disinfection<br>6 = Add bleach/chlorine<br>7 = Other<br>8 = Don't know**	**|__|**
126	If OTHER, specify	**Specify**________________________________	
127	What type of fuel does your household mostly use for cooking?<br><br>**Do not read list**	**1 = Dung<br>2 = Firewood/straw<br>3 = Charcoal<br>4 = Kerosene<br>5 = Gas<br>6 = Electricity<br>7 = Other**	**|__|**
128	Is the house connected to electricity?	**1 = Yes<br>2 = No**	**|__|**

In total, how many of the following items are owned by residents of this household?		**Enter number of items (zero if none)**	
	129	Wrist watch	**|__||__|**

**Add the household total for each item**	130	Gold (in grams)	|__||__|
	131	A kerosene lamp/pressure lamp	|__||__|
	132	A bed	|__||__|
	133	Non-mobile phone	|__||__|
	134	Mobile phone	|__||__|
	135	Bicycle	|__||__|
	136	Car	|__||__|
	137	Radio	|__||__|
	138	TV	|__||__|
	139	Fridge	|__||__|

140	Do you own this house?	**1 = Yes**<br>**2 = No**	|__|
141	Does any member of the household own any agricultural land?	**1 = Yes**<br>**2 = No-GO to 143**	|__|
142	How many hectares of agricultural land do members of this household own?	**Enter total number of hectares**<br>**(If less than 1, Enter in decimals (example 0.5)**<br>**Enter 9999 if hectares are not known**	|__||__|.|__||__|
143	Does this household own any livestock, herds, other farm animals, or poultry?	**1 = Yes**<br>**2 = No-Go to Section 2**	|__|

How many of the following animals does this household own?	**For each: Enter number. If none, enter 0**		
	144	Chickens	|__|__|__|
	145	Goats	|__|__|__|
	146	Sheep	|__|__|__|
	147	Donkeys	|__|__|__|
	148	Horses	|__|__|__|
	149	Mules	|__|__|__|
	150	Camels	|__|__|__|
	151	Milk cows	|__|__|__|
	152	Bulls	|__|__|__|

If there are no eligible women 13-49 in the household listing end interview here

Module 2

2. Identification of eligible women
**Interviewer: These questions are to be asked of all resident women aged 13-49 who were listed. Ask to see eligible women from household listing in question 118. Start with the oldest eligible woman.**<br><br>**For each woman listed by the household head you must first complete the consent procedure (up to 204) before proceeding with the interview.**<br><br>**If unable to interview or complete the interview, state the reason and next appointment**

Visits	**1st**	**2nd**	**3rd**
Date (dd/mm/yyyy)	|__|__| / |__|__| / |__|__|__|__|	|__|__| / |__|__| / |__|__|__|__|	|__|__| / |__|__| / |__|__|__|__|
Interviewer's name			
Result (Enter relevant code below)	|__|	|__|	|__|
If not completed, next visit appointment date(dd/mm/yyyy)	|__|__| / |__|__| / |__|__|__|__|	|__|__| / |__|__| / |__|__|__|__|	
If not completed, next visit appointment time			

Result Codes:
1. Completed
2. Partly completed
3. Postponed
4. Not at home
5. No-one competent to respond
6. Refused

If at the third visit, you are unable to complete the interview, please move on to the next woman in the household or the next household.

If possible please conduct the interview alone with the interviewee.

200	**Interviewer*:* Write name of the woman**	**|___|___|___|___|___|___|___|___|___|___|___|___|**	
201	**Interviewer*:* Write unique ID of the woman**<br><br>**(Obtain from: cluster ID = Q107 /household ID = Q108 /Person (woman) number = Q118)**	**|___|___|___| |___|___| |___|___|**<br>**cluster household woman**	
202	Interviewer's name	Name ______________________________	
203	**Interviewer*:* Have you read her the consent form?**	**1 = Yes**<br>**2 = No**	**|__|**
204	**Interviewer: Does the woman agree?**<br>**If no, end the interview here. Interview other eligible women in the household (if there are any). Start with Section 2 using a new questionnaire.**	**1 = Yes**<br>**2 = No**	**|__|**
**Thank you for agreeing to participate in this interview. I would like to first ask you about your involvement in the health development army (HDA)**			
205	Are you an HDA 1-5 leader?	**1 = Yes**<br>**2 = No**	**|__|**
206	Are you a 1 to 30 development team leader?	**1 = Yes**<br>**2 = No**	**|__|**

Now I would like to ask you about your pregnancy history.			
207	Are you currently pregnant?	**1 = Yes**<br>**2 = No**	**|__|**
208	Have you ever been pregnant before?	**1 = Yes**<br>**2 = No (end interview)**	**|__|**
209	Have you ever given birth to a child?	**1 = Yes**<br>**2 = No**	**|__|**
210	How many children have you given birth to in total?	**Enter number**	**|__|**
211	***I understand that it is not easy to talk about children who have died, or pregnancies that ended before full term, but it is important that you tell us about all of them, so that the government can develop programs to improve children's health.***<br><br>Have you ever had a pregnancy that didn't end up in a live birth?	**1 = Yes**<br>**2 = No (Go to 213)**	**|__|**

212	How many pregnancies have you had that did not end in a live birth?	**Enter number**	**|__|**
213	***Now would like to ask about the information regarding pregnancies beginning of 2004 (Ethiopian calendar).***<br><br>Were you pregnant at the time of Mäskäräm 2004 or any time afterwards?	**1 = Yes**<br>**2 = No (end interview)**	**|__|**

Now I would like to ask you about information on pregnancies* at the time of Mäskäräm 2004 or afterwards*. By this I mean all the pregnancies whether they ended up born alive, dead or even lost before full term pregnancy, whether they are still living or not, and whether they live with you or somewhere else.

Interviewer: start interview with a) asking the women to bring all the birth cards, if she has any, b) start with the birth outcome of the most recent pregnancy and work back to Mäskäräm 2004 (Ethiopian calendar). If the woman is currently pregnant do not include here – enter only pregnancies that have already ended.

For twin birth or two births, please record each baby separately (different lines, with different ID numbers)

Lost before full term is when baby was born before its due date, without woman going into labor.

ID Number of pregnancy	Outcome of pregnancy	Baby's name	Date of birth/Date Pregnancy ended	Born as a twin?	Sex	Still alive?	If still alive, how old in completed months?	If the child died, when did s/he die?
Start with most recent pregnancy	1 = Live birth<br>2 = Baby born dead<br>3= Lost before full term	Enter "not given" if not given a name	Enter 01 for day if not known.<br>Probe for months/years if not known	1 = Yes<br>2 = No<br>3=Don't know	1 = Male<br>2 = Female<br>3=Don't know	1 = Yes<br>2 = No	If less than a 28 days enter number of days	Enter 01 for day if not known.<br>Probe for months/years if not known
1	|__|		dd |__|__| mm |__|__| yyyy |__|__|__|__|	|__|	|__|	|__|	dd|__|__| mm |__|__|	dd |__|__| mm |__|__| yyyy |__|__|__|__|
2	|__|		dd |__|__| mm |__|__| yyyy |__|__|__|__|	|__|	|__|	|__|	dd|__|__| mm |__|__|	dd |__|__| mm |__|__| yyyy |__|__|__|__|
3	|__|		dd |__|__| mm |__|__| yyyy	|__|	|__|	|__|	dd|__|__| mm |__|__|	dd |__|__| mm |__|__| yyyy |__|__|__|__|

			|__|__|__|__|					
**4**	**|__|**		**dd |__|__|**<br><br>**mm |__|__|**<br><br>**yyyy |__|__|__|__|**	**|__|**	**|__|**	**|__|**	**dd|__|__|**<br><br>**mm |__|__|**	**dd |__|__|**<br><br>**mm |__|__|**<br><br>**yyyy |__|__|__|__|**
**5**	**|__|**		***dd* |__|__|**<br><br>***mm* |__|__|**<br><br>***yyyy* |__|__|__|__|**	**|__|**	**|__|**	**|__|**	**dd|__|__|**<br><br>**mm |__|__|**	**dd |__|__|**<br><br>**mm |__|__|**<br><br>**yyyy |__|__|__|__|**
**6**	**|__|**		***dd* |__|__|**<br><br>***mm* |__|__|**<br><br>***yyyy* |__|__|__|__|**	**|__|**	**|__|**	**|__|**	**dd|__|__|**<br><br>**mm |__|__|**	**dd |__|__|**<br><br>**mm |__|__|**<br><br>**yyyy |__|__|__|__|**

According to the given pregnancy history since* Mäskäräm 2004 *(Ethiopian), I would like to confirm that you have:			
214	Total number of live births = XX	**1 = Yes**<br>**2 = No**	**|__|**
215	Total number born dead = XX	**1 = Yes**<br>**2 = No**	**|__|**
216	Total number pregnancy loss before full term = XX	**1 = Yes**<br>**2 = No**	**|__|**

Note: In case of discrepancy, the interviewer will revisit the pregnancy history with the respondent women

<u>Continue with interview for each reported pregnancy since Mäskäräm 2004(Ethiopian).</u>

Module 3

Section 3. Pregnancy ID		
**Interviewer: Please obtain child information from the pregnancy event table.**		
300	**Write name of the child (if live birth)**	**|__|__|__|__|__|__|__|__|__|__|__|__|** Enter 99 if not live birth or name not given
301	**Write unique ID of the pregnancy Obtain from = cluster ID = Q107 /household ID = Q108 /Person (woman) number = Q118/pregnancy = pregnancy history table**	**|__|__|__| |__|__| |__|__| |__|__|** **cluster household woman pregnancy**

Section 4. Antenatal care (ANC)			
**Interviewer: in instances where the birth ended in a stillbirth or pregnancy was lost, please use the pregnancy number to refer to the birth (instead of baby name)**<br><br>***Now I want to talk to you about your pregnancy with the birth of [Child name / pregnancy number] (write no name if name was not given)***<br><br>**Abbreviation code**<br>**HDA = Health Development Army**<br>**HEW = Health Extension Worker**			
400	When pregnant with CHILD NAME / PREGNANCY NUMBER, did you inform anyone outside your family?	**1 = Yes**<br>**2 = No - GO to 404**	|__|
401	If you informed a health worker, who did you first inform about your pregnancy amongst health workers	**1 = HDA**<br>**2 = HEW**<br>**3 = Other health staff (i.e. nurse) at the health center**<br>**4 = Other**	|__|
402	If OTHER	Specify________________________	
403	What was the duration of your pregnancy when you informed an HEW, HDA or other health workers?	**Write number of weeks**<br>**99 if don't know**	|__|__|
404	Do you have a family health card with information about that pregnancy and birth?	**1 = Yes**<br>**2 = No – GO to 406**	|__|
405	***If yes:*** May I see your family health card?<br><br>**Interviewer: Is a family health card available?**	**1 = Yes**<br>**2 = No**	|__|

406	When pregnant with [CHILD NAME / PREGNANCY NUMBER], did you receive any care during pregnancy?<br><br>**Probe: care at the HP, at a health facility, or visits at home from a HEW/HDA**<br><br>**If no, skip ANC questions**	**1 = Yes**<br>**2 = No-GO to Section 6**	**|__|**

If **YES**, where did you get for ANC visits?<br><br>**Please select all that apply**	**For each: 1= Yes 2 = No**		
	407	Home	**|__|**
	408	Health Post	**|__|**
	409	Health Center	**|__|**
	410	Hospital	**|__|**
	411	Other	**|__|**
	412	(Specify)____________________	

**Antenatal care from a health post**			
413	Did you receive pregnancy care from a health post?	**1 = Yes**<br>**2 = No – GO to 422**	**|__|**
414	How many times did you attend the health post for pregnancy care in that pregnancy?	**Enter the number of times**<br>**99 if don't know**	**|__|__|**
415	When did your first visit to the health post take place?<br><br>**If this information is available from the family health card, use it.**	**dd |___|___| mm |___|___| yyyy |___|___|___|___|**<br>**Write 99/99/9999 if not available**	
416	**Interviewer: did Q415 information come from the woman or the card?**	**1 = Woman**<br>**2 = Card**	**|__|**
417	How old was your pregnancy at the first visit?	**Record number of weeks**<br>**99 if don't know**	**|__||__|**
418	**Interviewer: record gestation of the pregnancy at first ANC visit from family health card if available**	**Record number of weeks**<br>**99 if not available**	**|__||__|**

419	Can you tell us whether or not you were satisfied with the pregnancy care you received at the health post?<br><br>**Do not read list of options**	**1 = Yes was satisfied<br>2 = No was not satisfied (GO to 421)<br>3 = Neither satisfied nor dissatisfied (Go to 422)**	**|___|**
420	IF YES, then what was the level of satisfaction<br>**Read both options**	**1 = Fully satisfied-Go to 422<br>2 = Somewhat satisfied-Go to 422**	**|___|**
421	IF NO, then what was the level of dissatisfaction<br>**Read both options**	**1 = Fully dissatisfied<br>2 = Somewhat dissatisfied**	**|___|**

**Antenatal care from a health center**			
422	Did you receive pregnancy care from a health center?	**1 = Yes<br>2 = No– GO to 434**	**|__|**
423	Was your first ANC visit at a health center?	**1 = Yes<br>2 = No**	**|__|**
424	How many times did you attend the health center for pregnancy care in that pregnancy?	**Enter the number of times**<br>**Enter 99 if not known**	**|__||__|**
425	When did your first visit to the health center take place?<br><br>**Interviewer: Probe date of visit; record month and year if she does not remember date**	**dd |___|___| mm |___|___| yyyy |___|___|___|___| Write 99/99/9999 if not available**	
426	**Interviewer: Record from family health card if available.**	**dd |___|___| mm |___|___| yyyy |___|___|___|___| Write 99/99/9999 if not available from family health card.**	
427	How old was your pregnancy at the first visit?<br><br>**Record from family health card if available**	**Record number of weeks**<br>**99 if day is not known**	**|__||__|**
428	**Interviewer: did this information come from the woman or the card?**	**1 = Woman<br>2 = Card**	**|__|**
429	Who saw you at that first visit?	**1 = Nurse<br>2= Midwife<br>3=Health officer<br>4 = Other (HEW)<br>5=I don't know**	**|__|**
430	If **OTHER** please specify	Specify ______________________	
431	Can you tell us whether or not you were satisfied with the pregnancy care you received at the health center?<br><br>**Do not read list of options**	**1 = Yes was satisfied<br>2 = No was not satisfied (GO to 433)<br>3 = Neither satisfied nor dissatisfied (Go to 434)**	**|___|**
432	IF YES, then what was the level of satisfaction<br>**Read both options**	**1 = Fully satisfied -Go to 434<br>2 = Somewhat satisfied-Go to 434**	**|___|**

433	IF NO, then what was the level of dissatisfaction<br>**Read both options**	**1 = Fully dissatisfied**<br>**2 = Somewhat dissatisfied**	|___|

Antenatal care at home.			
434	Did you receive pregnancy carefroma health worker in your own home?	**1 = Yes**<br>**2 = No – GO to 445**	|__|
435	How many times were you visited at home by a health worker for pregnancy care in that pregnancy?	**Enter the number of times**	|__|
436	When did the first visit to you at home take place?<br>**Record from family health card if available**	**dd |___|___| mm |___|___| yyyy |___|___|___|___|**<br>**99 if day is not known**	
437	**Interviewer: did Q436 information come from the woman or the card?**	**1 = Woman**<br>**2 = Card**	|__|
438	How old was your pregnancy the first time that you were visited at home for pregnancy care?	**Record number of weeks**<br>**Enter 99 if not available.**	|__||__|
439	**Interviewer: Record from family health card if available.**	**Record number of weeks**<br>**Enter 99 if not available.**	|__||__|
440	Who was it that came to visit you the first time?	**1 = HEW**<br>**2 = HDA**<br>**3 = Other**	|__|
441	If **OTHER** please specify:	**Specify________________________________**	
442	Can you tell us whether or not you were satisfied with the pregnancy care you received at home?<br>**Do not read list of options**	**1 = Yes was satisfied**<br>**2 = No was not satisfied (GO to 444)**<br>**3 = Neither satisfied nor dissatisfied (Go to 445)**	|___|
443	**IF YES,** then what was the level of satisfaction<br>**Read both options**	**1 = Fully satisfied -Go to 445**<br>**2 = Somewhat satisfied-Go to 445**	|___|
444	**IF NO,** then what was the level of dissatisfaction<br>**Read both options**	**1 = Fully dissatisfied**<br>**2 = Somewhat dissatisfied**	|___|

Interviewer: I would now like to ask you about who provided specific elements of pregnancy care.

In instances where birth was a still birth or pregnancy was lost, use the pregnancy number instead of name.

When you were pregnant with CHILD NAME / PREGNANCY NUMBER, did you have the following at any time?

Interviewer: this applies to cares at home, health or health centre

445	Was your weight measured?	**1 = Yes**<br>**2 = No – GO to 448**	|__|

446	Which was the provider who did this the first time?<br><br>**Put 9 if not known**	**1 = HDA**<br>**2 = HEW**<br>**3 = Nurse/midwife**<br>**4 = Health Officer**<br>**5 = Doctor**<br>**6 = Other**	**|__|**
447	In which location was this service provided?	**1 = Home**<br>**2 = Health Post**<br>**3 = Health Centre**<br>**4 = Hospital**<br>**5 = Other**	**|__|**
448	Was your height measured?	**1 = Yes**<br>**2 = No – GO to 451**	**|__|**
449	Which was the provider who did this the first time?<br><br>**Put 9 if not known**	**1 = HDA**<br>**2 = HEW**<br>**3 = Nurse/midwife**<br>**4 = Health Officer**<br>**5 = Doctor**<br>**6 = Other**	**|__|**
450	In which location was this service provided?	**1 = Home**<br>**2 = Health Post**<br>**3 = Health Centre**<br>**4 = Hospital**<br>**5 = Other**	**|__|**
451	Did you receive information about breastfeeding]?	**1 = Yes**<br>**2 = No – GO to 454**	**|__|**
452	Which was the provider who did this the first time?<br><br>**Put 9 if not known**	**1 = HDA**<br>**2 = HEW**<br>**3 = Nurse/midwife**<br>**4 = Health Officer**<br>**5 = Doctor**<br>**6 = Other**	**|__|**
453	In which location was this service provided?	**1 = Home**<br>**2 = Health Post**<br>**3 = Health Centre**<br>**4 = Hospital**<br>**5 = Other**	**|__|**
454	Was your blood pressure tested?<br><br>**(PROBE: when a strap was put around your upper arm and a measure taken)**	**1 = Yes**<br>**2 = No – GO to 457**	**|__|**
455	Which was the provider who did this the first time?<br><br>**Put 9 if not known**	**1 = HDA**<br>**2 = HEW**<br>**3 = Nurse/midwife**<br>**4 = Health Officer**<br>**5 = Doctor**<br>**6 = Other**	**|__|**
456	In which location was this service provided?	**1 = Home**<br>**2 = Health Post**<br>**3 = Health Centre**<br>**4 = Hospital**<br>**5 = Other**	**|__|**
457	Did you give a urine sample for a test?	**1 = Yes**<br>**2 = No – GO to 460**	**|__|**
458	Which was the provider who did this the first time?<br><br>**Put 9 if not known**	**1 = HDA**<br>**2 = HEW**<br>**3 = Nurse/midwife**<br>**4 = Health Officer**<br>**5 = Doctor**<br>**6 = Other**	**|__|**
459	In which location was this service provided?	**1 = Home**<br>**2 = Health Post**<br>**3 = Health Centre**<br>**4 = Hospital**<br>**5 = Other**	**|__|**

460	Did you give blood for any test for syphilis?	**1 = Yes**<br>**2 = No – GO to 463**<br>**3 = Don't know – GO to 463**	|__|
461	Which was the provider who did this the first time?<br><br>**Put 9 if not known**	**1 = HDA**<br>**2 = HEW**<br>**3 = Nurse/midwife**<br>**4 = Health Officer**<br>**5 = Doctor**<br>**6 = Other**	|__|
462	In which location was this service provided?	**1 = Home**<br>**2 = Health Post**<br>**3 = Health Centre**<br>**4 = Hospital**<br>**5 = Other**	|__|
463	Did you receive iron folate tablets or iron syrup?<br><br>**Interviewer: show a sample or picture of iron tables/syrup and folate tablet**	**1 = Yes**<br>**2 = No – GO to 467**	|__|
464	Which was the provider who did this the first time?<br><br>**Put 9 if not known**	**1 = HDA**<br>**2 = HEW**<br>**3 = Nurse/midwife**<br>**4 = Health Officer**<br>**5 = Doctor**<br>**6 = Other**	|__|
465	In which location was this service provided?	**1 = Home**<br>**2 = Health Post**<br>**3 = Health Centre**<br>**4 = Hospital**<br>**5 = Other**	|__|
466	**If yes:** For how many days did you take the tablets or syrup?	**Enter number of days**<br>**Enter 999 if not known**	|__||__||__|
467	Were you given an injection in the arm to prevent the baby from getting tetanus, that is, convulsions after birth?<br><br>**(On field manual please explain the need to describe tetanus toxid shots part)**	**1 = Yes**<br>**2 = No – GO to 473**	|__|
468	**If yes:** How many times did you get a tetanus injection?	**Write number of times**	|__||__|
469	**If less than 2 times:** At any time before this pregnancy did you receive any tetanus injections?	**1 = Yes**<br>**2 = No – GO to 473**	|__|
470	**If 469 is answered YES:** Before this pregnancy, how many times did you receive a tetanus injection?	**Enter number of times**<br>**Enter 99 if not known**	|__||__|
471	**If 469 is answered YES:** How many years ago did you receive the last tetanus injection before this pregnancy?	**Write number of years ago**	|__||__|
472	In which health facility was this service provided?	**1 = Home**<br>**2 = Health Post**<br>**3 = Health Centre**<br>**4 = Hospital**<br>**5 = Other**	|__|
473	Did you receive HIV information?	**1 = Yes**<br>**2 = No – GO to 476**	|__|
474	Which was the provider who did this the first time?<br><br>**Put 9 if not known**	**1 = HDA**<br>**2 = HEW**<br>**3 = Nurse/midwife**<br>**4 = Health Officer**<br>**5 = Doctor**<br>**6 = Other**	|__|
475	In which location was this service provided?	**1 = Home**	|__|

		**2 = Health Post**<br>**3 = Health Centre**<br>**4 = Hospital**<br>**5 = Other**	
476	Did you receive HIV testing?	**1 = Yes**<br>**2 = No – GO to 479**	**|__|**
477	Which was the provider who did this the first time?<br><br>**Put 9 if not known**	**1 = HDA**<br>**2 = HEW**<br>**3 = Nurse/midwife**<br>**4 = Health Officer**<br>**5 = Doctor**<br>**6 = Other**	**|__|**
478	In which location was this service provided?	**1 = Home**<br>**2 = Health Post**<br>**3 = Health Centre**<br>**4 = Hospital**<br>**5 = Other**	**|__|**
479	Did you receive STI testing?	**1 = Yes**<br>**2 = No – GO to 482**	**|__|**
480	Which was the provider who did this the first time?<br><br>**Put 9 if not known**	**1 = HDA**<br>**2 = HEW**<br>**3 = Nurse/midwife**<br>**4 = Health Officer**<br>**5 = Doctor**<br>**6 = Other**	**|__|**
481	In which location was this service provided?	**1 = Home**<br>**2 = Health Post**<br>**3 = Health Centre**<br>**4 = Hospital**<br>**5 = Other**	**|__|**
482	Did you receive any STI treatment?	**1 = Yes**<br>**2 = No – GO to 485**	**|__|**
483	Which was the provider who did this the first time?<br><br>**Put 9 if not known**	**1 = HDA**<br>**2 = HEW**<br>**3 = Nurse/midwife**<br>**4 = Health Officer**<br>**5 = Doctor**<br>**6 = Other**	**|__|**
484	In which location was this service provided?	**1 = Home**<br>**2 = Health Post**<br>**3 = Health Centre**<br>**4 = Hospital**<br>**5 = Other**	**|__|**
485	Did you receive information on nutrition?	**1 = Yes**<br>**2 = No – GO to 488**<br>**3 = Not applicable – GO to 488**	**|__|**
486	Which was the provider who did this the first time?<br><br>**Put 9 if not known**	**1 = HDA**<br>**2 = HEW**<br>**3 = Nurse/midwife**<br>**4 = Health Officer**<br>**5 = Doctor**<br>**6 = Other**	**|__|**
487	In which location was this service provided?	**1 = Home**<br>**2 = Health Post**<br>**3 = Health Centre**<br>**4 = Hospital**<br>**5 = Other**	**|__|**

488	Did you receive information on danger signs?	**1 = Yes**<br>**2 = No – GO to 491**	|__|
489	Which was the provider who did this the first time?<br><br>**Put 9 if not known**	**1 = HDA**<br>**2 = HEW**<br>**3 = Nurse/midwife**<br>**4 = Health Officer**<br>**5 = Doctor**<br>**6 = Other**	|__|
490	In which location was this service provided?	**1 = Home**<br>**2 = Health Post**<br>**3 = Health Centre**<br>**4 = Hospital**<br>**5 = Other**	|__|
491	Were you advised on birth preparedness and complication readiness?<br><br>**Probe: for finances, for help during delivery, for transport, for emergencies?**	**1 = Yes**<br>**2 = No – GO to 501**	|__|
492	Which was the provider who did this the first time?<br><br>**Put 9 if not known**	**1 = HDA**<br>**2 = HEW**<br>**3 = Nurse/midwife**<br>**4 = Health Officer**<br>**5 = Doctor**<br>**6 = Other**	|__|
493	In which location was this service provided?	**1 = Home**<br>**2 = Health Post**<br>**3 = Health Centre**<br>**4 = Hospital**<br>**5 = Other**	|__|
494	Was your birth preparedness and complication readiness plan recorded?	**1 = Yes**<br>**2 = No – GO to 501**	|__|
495	Which was the provider who did this the first time?<br><br>**Put 9 if not known**	**1 = HDA**<br>**2 = HEW**<br>**3 = Nurse/midwife**<br>**4 = Health Officer**<br>**5 = Doctor**<br>**6 = Other**	|__|
496	In which location was this service provided?	**1 = Home**<br>**2 = Health Post**<br>**3 = Health Centre**<br>**4 = Hospital**<br>**5 = Other**	|__|
**Section 5. Antenatal care continued**			
500	Is there a record in family health card about her birth preparedness and complication plan?<br><br>**Ask to see Family Health Card, if available.**	**1 = Yes**<br>**2 = No**<br>**3= No family health card**	|__|

Can you tell me danger signs during pregnancy?<br><br>**Do not read list. Do not probe. This is about knowledge of danger signs, not**	**For each: 1 = Yes 2 = No**		
	501	Vaginal bleeding	|__|
	502	Severe abdominal pain	|__|

**about her own specific experience.**	503	Offensive discharge from the birth canal	|__|
	504	Fever	|__|
	505	Headache, dizziness, or blurred vision	|__|
	506	Convulsions or unconsciousness	|__|
	507	Swollen hands and face	|__|

Can you tell me the components of birth preparedness and complication readiness plan<br><br>**Do not read list. Do not probe. This is about knowledge, not about her own specific experience with planning.**	**For each: 1 = Yes 2 = No**		
	508	Financial	|__|
	509	Transport	|__|
	510	Nutritious and sufficient food	|__|
	511	Identify birth attendant	|__|
	512	Identify facility where you would give birth	|__|
	513	Identification of blood donor, if required	|__|
	514	Clean clothes	|__|
	515	Cover to deliver on	|__|
	516	Gloves	|__|
	517	Cotton gauze	|__|
	518	Soap	|__|
	519	New razor blades	|__|
	520	Sterilized scissors	|__|
	521	Sterilized thread	|__|

522	Did you make any preparations for your delivery?<br><br>**Probe: for finances, for help during delivery, for transport, for emergencies**	**1 = Yes**<br>**2 = No – GO to 539**	|__|

**If YES:** What preparations did you make for your delivery?<br><br>**Probe but do not read out the list. Select all that apply.**	**For each: 1 = Yes 2 = No**		
	523	Financial	|__|
	524	Transport	|__|
	525	Nutritious and sufficient food	|__|
	526	Identify birth attendant	|__|
	527	Identify facility where you would give birth	|__|
	528	Identification of blood donor, if required	|__|
	529	Clean clothes	|__|

	530	Cover to deliver on	|__|
	531	Gloves	|__|
	532	Cotton gauze	|__|
	533	Soap	|__|
	534	New razor blades	|__|
	535	Sterilized scissors	|__|
	536	Sterilized thread	|__|
	537	Other	|__|
	538	Specify ________________________________	

539	During your last pregnancy have you attended a pregnant women's conference/Forum?	**1 = Yes**<br>**2 = No- GO to 552**	|__|
540	If yes, how many times did you attend pregnant women's forum in your last pregnancy?	**Enter number of times**<br>**Enter 99 if not known**	|__||__|

What was discussed in the pregnant women's forum?<br><br>**Probe but do not read out the list. Select all that apply.**	**For each: 1 = Yes 2 = No**		
	541	Birth preparedness	|__|
	542	Importance of ANC	|__|
	543	Institutional delivery	|__|
	544	Importance of post natal checks	|__|
	545	Seeking newborn care	|__|
	546	Other (specify)	|__|
	547	Specify |____________________________________|	

548	Who informed you about the pregnant women's forum?	**1 = HDA**<br>**2 = HEW**<br>**3 = Other (Specify)**	|__|
549	If **OTHER**, specify	Specify______________________	
550	Where did the pregnant women conference take place?	**1 = at HDA's place**<br>**2 = at gote/kebele meeting place**<br>**3 = health post**<br>**4 = health centre**<br>**5 = Other**	
551	If **OTHER**, specify	Specify______________________	
552	Can you tell us whether or not you were satisfied with the care you received while you were pregnant?<br>**Do not read list of options**	**1 = Yes was satisfied**<br>**2 = No was not satisfied (GO to 554**<br>**3 = Neither satisfied nor dissatisfied (Go to 555)**	|___|

553	IF YES, then what was the level of satisfaction<br>**Read both options**	**1 = Fully satisfied-Go to 555**<br>**2 = Somewhat satisfied-Go to 555**	|___|
554	IF NO, then what was the level of dissatisfaction<br>**Read both options**	**1 = Fully dissatisfied**<br>**2 = Somewhat dissatisfied**	|___|

**Interviewer**<br><br>If the interviewee has reported having facility based care for ANC for this birth, ask the following questions			
When having a pregnancy related medical examination at a health facility, did you experience any of the following with someone?<br><br>**Select all that apply**	**For each: 1 = Yes 2 = No**		
	555	Encourage you to ask questions about delivery	|___|
	556	Give you the choice for choosing any specific option (date of delivery, normal or operative delivery etc.)	|___|
	557	Unnecessarily motivating you for having C-section	|___|
	558	Give explanation on the procedure s/he is going to use (e.g. normal delivery or c-section or time that is going to take)	|___|
	559	Maintain privacy while examining you (e.g. placed a curtain/separator before examination)	|___|
	560	Use non-dignified language (for example passing insulting and/or degrading statements about you/family/community/ethnicity) during examination	|___|
	561	The primary care (senior doctor)provider deny to provide you services because you belong to any specific ethnic group/community	|___|
	562	Use harsh tone or shouted on your during examination	|___|
	563	Use abusive language with you during examination	|___|
	564	Threaten you e.g. if you do not cooperate I will do the caesarean operation during delivery	|___|
	565	Leave you abandoned during examination	|___|
	566	Share results/diagnosis of medical reports with you during examination	|___|
	567	Share the results when other could easily hear	|___|
	568	Assure you that your medical information/records will be kept confidential	|___|
	569	Deny you providing any specific services (that you asked for) due to lack of money	|___|

If outcome of pregnancy is pregnancy lost (Outcome #3 in pregnancy history) stop interview and Check for other pregnancy

**6. Delivery care**			
**Now I have some questions to ask you about what happened to you during and after the delivery for CHILD NAME / PREGNANCY NUMBER**			
600	Where did you give birth?	**1 = Home- GO to 602**<br>**2 = Health post – GO to 609**<br>**3 = Health centre – GO to 609**<br>**4 = Hospital – GO to 609**<br>**5 = Other (specify)**	**|__|**
601	If OTHER, please specify:	**Specify |______________________________|**	

	**For each: 1 = Yes 2 = No**		
If delivered at home, why?<br><br>**Select all that apply**	602	Always delivered at home	**|__|**
	603	Husband/mother/mother-in-law does not allow	**|__|**
	604	Don't like health facilities	**|__|**
	605	Expensive to go to health facilities	**|__|**
	606	Tradition/religious reason	**|__|**
	607	Other	**|__|**
	608	Specify |___________________________________|	
	**For each: 1 = Yes 2 = No**		
If delivered in health post, health centre or hospital, why?<br><br>**Select all that apply**	609	Always delivered at a health facility	**|__|**
	610	Due to existing complications	**|__|**
	611	Referred by HDA/HEW	**|__|**
	612	Advised at pregnant women's forum	**|__|**
	613	Taken to health facility due to prolonged labour or delivery related risks	**|__|**
	614	Convenient	**|__|**
	615	Affordable (free)	**|__|**
	616	Other	**|__|**
	617	Specify |___________________________________|	

618	Who was the primary person that assisted you with the delivery?	**1 = Doctor**<br>**2 = Nurse/midwife**<br>**3 = Health extension worker**<br>**4 = HDA**<br>**5 = traditional birth attendant**<br>**6 = Relative/friend**<br>**7 = Nobody**<br>**8 = Other**	|__|

Who else was present at the delivery?<br>**Select all that apply**	**For each: 1 = Yes 2 = No**		
	619	Doctor	|__|
	620	Nurse/midwife	|__|
	621	Health extension worker	|__|
	622	HDA	|__|
	623	Traditional birth attendant	|__|
	624	Relative/friend	|__|
	625	Nobody	|__|
	626	Other	|__|

627	When you gave birth, did the main person assisting you wash her/his hands with soap before the delivery?	**1 = Yes**<br>**2 = No**<br>**3 = Don't know**	|__|
628	When you gave birth, did the main person assisting you wear gloves during delivery?	**1 = Yes**<br>**2 = No**<br>**3 = Don't know**	|__|
629	When you gave birth, did the delivery take place on a clean surface? (**Clean surface: clean space or carpet or mat**)	**1 = Yes**<br>**2 = No**<br>**3 = Don't know**	|__|
630	Where you given a drug called misoprostol to help control bleeding? These are pills.	**1 = Yes**<br>**2 = No**<br>**3 = Don't know**	|__|
631	**Interviewer: ask the following if the woman gave birth at a health facility, if no, SKIP to 633**<br><br>After giving birth, how many days did you stay at the health facility in total?	**Enter number of days.**<br>**Enter 0 if the woman left on the same day as delivery**	|__|__|
632	Was [CHILD NAME/ PREGNANCY NUMBER] delivered by caesarean, that is, did they cut your belly open to take the baby out?	**1 = Yes**<br>**2 = No**	|__|

During the delivery of [CHILD NAME / PREGNANCY NUMBER] did you experience any of the following?<br><br>**Read out the list**	**For each 1 = Yes 2 = No**		
	633	Heavy bleeding	|__|
	634	Labour more than 12 hours	|__|
	635	Loss of consciousness	|__|

	636	Premature labour	|__|
	637	Foul discharge	|__|
	638	Baby in abnormal position	|__|

639	During delivery were you advised to go to a higher health facility to get special care?	**1 = Yes**<br>**2 = No – GO to 646**	|__|
640	What was the reason for which you were referred?<br><br>**Interviewer: please check if there is a record on referral; danger signs = see the list**	**1 = Due to existence of one or more danger signs**<br>**2 = Due to lack of equipment/space**<br>**3 = Due to lack of trained human resources**<br>**4 = Other**	|__|
641	**If OTHER,** please specify	**Specify**___________________________	
642	When you were referred to a higher health facility, did you go to the higher health facility?	**1 = Yes – GO to 645**<br>**2 = No**	|__|
643	If **NO**, why?	**1 = Facility was too far**<br>**2 = Cost too much money**<br>**3 = Don't like going to different facility**<br>**4 = No permission to go**<br>**5 = Don't like the quality of care provided**<br>**6 = Other**	|__|
644	**If OTHER** please specify	**Specify**___________________________	
645	Was ambulance made available for you for that referral?	**1 = Yes**<br>**2 = No**	|__|
646	Can you tell us whether or not you were satisfied with the delivery care you received?<br>**Do not read list of options**	**1 = Yes was satisfied**<br>**2 = No was not satisfied (GO to 648)**<br>**3 = Neither satisfied nor dissatisfied (Go to 649)**	|___|
647	IF YES, then what was the level of satisfaction<br>**Read both options**	**1 = Fully satisfied -Go to 649**<br>**2 = Somewhat satisfied -Go to 649**	|___|
648	IF NO, then what was the level of dissatisfaction<br>**Read both options**	**1 = Fully dissatisfied**<br>**2 = Somewhat dissatisfied**	|___|

**Interviewer**<br><br>Only ask if participant reported having given a birth at a facility ask the following questions (649– 670)			
During a birth at a health facility, did you experience any of the following with someone?<br><br>**Select all that apply**	**For each: 1 = Yes 2 = No**		
	649	Regularly explain and inform you about the progress or procedure being performed	|___|
	650	Cover you while taking to the delivery room	|___|
	651	Delay in providing care after a decision has been made e.g. caesarean section	|___|
	652	Use abusive language with you during the delivery	|___|
	653	Leave you abandoned during the delivery	|___|
	654	Perform any procedure without your consent or informing you (like caesarean sections, blood transfusion, sterilization etc.)	|___|
	655	Ignore you while asking pain relief/medication during delivery	|___|
	656	Use harsh tone or shouted on you during the delivery	|___|
	657	Slap you during the delivery	|___|
	658	Pinch you irritably during the delivery	|___|
	659	Beat you during the delivery	|___|
	660	Push you badly to change your position during the delivery	|___|
Soon after giving birth at a facility, did you experience any of the following with someone?<br><br>**Select all that apply**	**For each: 1 = Yes 2 = No**		
	661	Encourage you to ask questions post-delivery	|___|
	662	Cover you after the delivery	|___|
	663	Ignore you while asking pain relief/medication after the delivery	
	664	Leave you abandoned immediately after the delivery	|___|
	665	Use harsh tone or shouted on you after the delivery	|___|
	666	Use abusive language with you after the delivery	|___|
	667	Ask you to clean delivery couches post-delivery	|___|
	668	Ask you to clean dirty bathroom/toilets post-delivery	|___|
	669	Detain for non/partial-payment (e.g. keep you or your child in custody for full	|___|

		payment)	
670	Did you know where to go to lodge a complain	1 = Yes<br>2 = No	|___|

**7. Postnatal care of the mother (PNC)**			
**Now I want to ask you about any postnatal checks you had for your own health after that birth**			
700	Did anyone check on your <u>own</u> health within 6 weeks of giving birth?<br><br>**Probe to see if check was done by HEW/HC staff/HDA/Nurse/Doctor.**	**1 = Yes**<br>**2 = No – GO to Section 8**	**|__|**
701	How many days after giving birth did you have your first health check?<br><br>**Clarify that this is a health check for the mother**	**Enter number of days**<br>**Enter 99 if not known**	**|__|__|**
702	Where did the first check take place?	**1 = In own home**<br>**2 = Health post**<br>**3 = Health centre**<br>**4 = Hospital**<br>**5 = Other**	**|__|**
703	By whom?<br>**Put 9 if not known**	**1 = Doctor**<br>**2 = Nurse/midwife**<br>**3 = HEW**<br>**4 = Health Officer/Assistant**<br>**5 = HDA**	**|__|**
704	Was a second visit conducted?	**1 = Yes**<br>**2 = No – GO to 712**	**|__|**
705	How many days after giving birth did you have your second health check?<br>**Clarify that this is a health check for the mother.**	**Enter number of days**<br>**Enter 99 if not known**	**|__|__|**
706	Where did the checking take place?	**1 = In own home**<br>**2 = Health post**<br>**3 = Health centre**<br>**4 = Hospital**<br>**5 = Other**	**|__|**
707	By whom?<br>**Put 9 if not known**	**1 = Doctor**<br>**2 = Nurse/midwife**<br>**3 = HEW**<br>**4 = Health Officer/Assistant**<br>**5 = HDA**	**|__|**
708	Was a third visit conducted?	**1 = Yes**<br>**2 = No – GO to 712**	**|__|**
709	How many days after giving birth did you have your third health check?<br>**Clarify that this is a health check for the mother.**	**Enter number of days**<br>**Enter 99 if not known**	**|__|__|**
710	Where did the checking take place?	**1 = In own home**<br>**2 = Health post**<br>**3 = Health centre**<br>**4 = Hospital**<br>**5 = Other**	**|__|**

711	By whom?<br>**Put 9 if not known**	**1 = Doctor<br>2 = Nurse/midwife<br>3 = HEW<br>4 = Health Officer/Assistant<br>5 = HDA**	**|__|**

During visits after giving birth, what was done to check your health?

Read List out loud

Mark all that apply

If the visit did not happen at specified time, leave that column of answers blank

	**For each: 1 = Yes 2 = No**					
	(Question #)	0-2 days	(Question #)	3-7 days	(Question #)	8-42 days
Checked breasts	712	|__|	713	|__|	714	|__|
Advised breast feeding	715	|__|	716	|__|	717	|__|
Oriented about danger signs	718	|__|	719	|__|	720	|__|
Educated on family planning	721	|__|	722	|__|	723	|__|
Information given on nutrition	724	|__|	725	|__|	726	|__|
Referred to a health facility	727	|__|	728	|__|	729	|__|
Measured Blood Pressure	730	|__|	731	|__|	732	|__|
Checked/treated birth-related wound (if applicable)	733	|__|	734	|__|	735	|__|
Other	736	|__|	737	|__|	738	|__|
Specify ___________						

739	Can you tell us whether or not you were satisfied with the post-natal care you received?<br><br>**Interviewer: explain that this is care for the mother, not the newborn.Do not read list of options**	**1 = Yes was satisfied<br>2 = No was not satisfied (GO to 741)<br>3 = Neither satisfied nor dissatisfied (Go to Section 8)**	**|___|**
740	IF YES, then what was the level of satisfaction<br>**Read both options**	**1 = Fully satisfied (Go to Section 8)<br>2 = Somewhat satisfied(Go to Section 8)**	**|___|**
741	IF NO, then what was the level of dissatisfaction<br>**Read both options**	**1 = Fully dissatisfied<br>2 = Somewhat dissatisfied**	**|___|**

INTERVIEWER: IF THE PREGNANCY OUTCOME IN THE PREGNANCY HISTORY TABLE WAS#2 (STILLBIRTH), END THE INTERVIEW HERE AND CHECK FOR OTHER PREGNANCIES

8. Immediate newborn care			
**Now I have some questions about what happened to [CHILD NAME] at birth and immediately afterwards.**			
800	**Interviewer: What is the pregnancy ID number of [CHILD NAME]?**	**Enter ID number from the table**	**|__|**
801	Was this a single birth?	**1 =Yes**<br>**2 =No**	**|__|**
802	Was [CHILD NAME] weighed at birth?	**1 = Yes**<br>**2 = No – GO to 804**<br>**3 = Don't know– GO to 804**	**|__|**
803	**If YES**: how much did [CHILD NAME] weigh at birth?	**Enter weight in grams e.g. if the weight was 1.9 kilogrammes enter 1900; don't know write 9999. Use the weight recorded on the card if possible**	**|__|__|__|__|**
804	Did [CHILD NAME] have any difficulty crying/breathing at birth?	**1 = Yes**<br>**2 = No – GO to 807**<br>**3= Don't know**	**|__|**

Did anyone do any of the following to [CHILD NAME] immediately after birth?<br><br>**Select all that apply**	**For each: 1 = Yes 2 = No**		
	805	Rubbing/stimulating	|__|
	806	Mouth-to-mouth/resuscitation	|__|

807	Where was [CHILD NAME] placed immediately after delivery?	**1 = Alone/on the floor**<br>**2 = On the mother's Belly/chest**<br>**3 = Beside the mother**<br>**4 = With someone else**<br>**5 = Other**<br>**6 = Don't know**	**|__|**
808	How long after [CHILD NAME] was born was s/he dried/wiped?<br><br>***Check for time after the baby was born, not time after the placenta came out.***	**Enter in minutes,**<br>**999 if don't know.**	**|__||__||__|**
809	How long after [CHILD NAME] was born was s/he wrapped in a cloth?<br><br>***Check for time after the baby was born, not time after the placenta came out.***	**Enter in minutes,**<br>**999 if don't know.**	**|__||__||__|**

810	What was used to tie the cord?	**1 = New string/thread**<br>**2 = Boiled string/thread**<br>**3 = Any string/thread**<br>**4 = Clamp**<br>**5 = Nothing**<br>**6 = Don't know**<br>**7 = Other**	**|__|**
811	What was used to cut the cord?	**1 = new razor blade**<br>**2 = any razor blade**<br>**3 = sterilized scissors**<br>**4 = any scissors**<br>**5 = don't know**<br>**6 = other**	**|__|**
812	Was anything applied to the cord after cutting and tying?	**1 = Yes**<br>**2 = No – GO to 824**	**|__|**

		**For each: 1 = Yes 2 = No**	
**If yes:** What was applied to the cord just after cutting the cord?<br><br>**Do not prompt, select all that apply**	813	Butter	|__|
	814	Ash	|__|
	815	Ointment (non-medicinal)	|__|
	816	Animal dung	|__|
	817	Oil	|__|
	818	Cold water	|__|
	819	Other	|__|

820	Was an antiseptic applied to the cord?	**1 = Yes**<br>**2 = No – GO to 824**<br>**3 = Don't know– GO to 824**	**|__|**
821	If yes, was this chlorhexidine?	**1 = Yes**<br>**2 = No– GO to 824**<br>**3 = Don't know– GO to 824**	**|__|**
822	If YES, for how many days was chlorhexidine applied?	**Enter number of days**<br>**Enter 99 if not known**	**|__||__|**
823	If YES, how many times per day was chlorhexidine applied?	**Enter number of times applied per day**<br>**Enter 99 if not known**	**|__||__|**
824	Did [CHILD NAME] receive TTC eye ointment soon after delivery?	**1 = Yes**<br>**2 = No**<br>**3 = Don't know**	|__|
825	When [CHILD NAME] was born, how soon did you bathe him/her?	**1 = In the first hour**<br>**2 = After one hour – GO to 827**<br>**3 = After one day– GO to 828**	|__|
826	**If in the first hour:** After how many minutes would you say?	**Write number of minutes.**<br>**Enter 99 if don't know.**	|__||__|
827	**If after one hour:** After how many hours would you say?	**Write number of full hours.**<br>**e.g. if response is 'after one hour' enter 1, if response is 'after one and a half hours' enter 1, if**	|__||__|

		**response is 'after two and a half hours' enter 2.**<br>**Enter 99 if don't know.**	
828	**If after one day:** After how many days would you say?	**Write number of full days.**<br><br>**e.g. if response is 'after one day' enter 1, if response is 'after one and a half days' enter 1.**<br>**Enter 99 if don't know.**	|__||__|
829	In the first week of life, did you hold [CHILD NAME] skin to skin against your breasts during the daytime and night-time?	**1 = Yes, 1-7 times a day**<br>**2 = Yes, 8-12 times a day**<br>**3 = Yes, more than 12 times a day**<br>**4 = Never**<br>**5 = Don't know**	|__|
830	In the first week of life, did you sleep with [CHILD NAME] against you at night, or did you lay him/her alone on the bed or elsewhere?	**1 = Slept with mother**<br>**2 = Baby slept alone**<br>**3 = Baby slept with another person**	|__|
831	Did you ever breastfeed [CHILD NAME]?	**1 = Yes**<br>**2 = No**	|__|
832	Did you exclusively breastfed [CHILD NAME] during the first 28 days of life?	**1 = Yes – GO to 837**<br>**2 = No**	|__|
833	If No, what did you mainly give [CHILD NAME], other than breastmilk?	**1 = Water**<br>**2 = Non-human milk**<br>**3 = Butter**<br>**4 = Sugar/glucose/salt water solution**<br>**5 = Juice**<br>**6 = Tea/infusions**<br>**7 = Other**	|__|
834	If OTHER, specify	**Specify** ____________________	
835	And, why did you provide other drinks besides your breast milk?	**1 = My breast milk is not enough**<br>**2 = I do not stay with NAME throughout the day**<br>**3 = Advised by friends or relatives to do so**<br>**4 = Tradition or culture**<br>**5 = Other**	|__|
836	If OTHER, specify	**Specify** ____________________	
837	How long after birth did you first put [CHILD NAME] to the breast?	**1 = In the first hour**<br>**2 = After one hour but during the first day**<br>**3 = After the first day of life**	|__|
838	Did you squeeze out and throw away the first milk?	**1 = Yes**<br>**2 = No**	|__|

839	In the first 6 weeks after [CHILD NAME] was born, did any health care provider excluding traditional birth attendant check on his/her health?<br><br>**Probe for checks done at the place of birth on the same day as birth, and checks after.**	**1 = Yes**<br>**2 = No - GO to 892**	**|__|**
840	If YES, how many days after birth was [CHILD NAME]'s first health check?	**Enter number of days**<br>**Enter 99 if not known**	**|__|__|**
841	If YES, where did the health checks on [CHILD NAME] take place?	**1 = In own home**<br>**2 = Health post**<br>**3 = Health centre**<br>**4 = Hospital**<br>**5 = Other**	**|__|**
842	If YES, who checked on the health of [CHILD NAME]?<br>**Probe for most qualified person**	**1 = Doctor**<br>**2 = Nurse/midwife**<br>**3 = HEW**<br>**4 = Health Officer/Assistant**<br>**5 = HDA**<br>**6=Don't know**	**|__|**
843	Was a second check conducted?	**1 = Yes**<br>**2 = No - GO to 855**	**|__|**
844	If YES, how many days after birth was [CHILD NAME]'s second health check?	**Enter number of days**<br>**Enter 99 if not known**	**|__|__|**
845	If YES, Where did the health checks on [CHILD NAME] take place?	**1 = In own home**<br>**2 = Health post**<br>**3 = Health centre**<br>**4 = Hospital**<br>**5 = Other**	**|__|**
846	If YES, Who checked on the health of [CHILD NAME]?<br>**Probe for most qualified person**	**1 = Doctor**<br>**2 = Nurse/midwife**<br>**3 = HEW**<br>**4 = Health Officer/Assistant**<br>**5 = HDA**<br>**6=Don't know**	**|__|**
847	Was a third check conducted?	**1 = Yes**<br>**2 = No - GO to 855**	**|__|**
848	If YES- how many days after birth was [CHILD NAME]'s third health check?	**Enter number of days**<br>**Enter 99 if not known**	**|__|__|**
849	If YES, Where did the health checks on [CHILD NAME] take place?	**1 = In own home**<br>**2 = Health post**<br>**3 = Health centre**<br>**4 = Hospital**<br>**5 = Other**	**|__|**
850	If YES, Who checked on the health of [CHILD NAME]?<br>**Probe for most qualified person**	**1 = Doctor**<br>**2 = Nurse/midwife**<br>**3 = HEW**<br>**4 = Health Officer/Assistant**<br>**5 = HDA**<br>**6=Don't know**	**|__|**

851	Was a forth check conducted?	**1 = Yes**<br>**2 = No - GO to 855**	**|__|**
852	If YES, how many days after birth was [CHILD NAME]'s forth health check?	**Enter number of days**<br>**Enter 99 if not known**	**|__|__|**
853	Where did the checking take place?	**1 = In own home**<br>**2 = Health post**<br>**3 = Health centre**<br>**4 = Hospital**<br>**5 = Other**	**|__|**
854	By whom?	**1 = Doctor**<br>**2 = Nurse/midwife**<br>**3 = HEW**<br>**4 = Health Officer/Assistant**<br>**5 = HDA**<br>**6=Don't know**	**|__|**

At any of the health checks, what was done to check the health of [CHILD NAME]?

Interviewer: Read list out loud. Mark all that apply

If the visit did not happen at the specified time, leave that column of answers blank

	**For each: 1 = Yes 2 = No**					
	(Question #)	0-2 days	(Question #)	3-7 days	(Question #)	8-42 days
Generally examined /looked at baby's body	855	|__|	856	|__|	857	|__|
Weighed baby	858	|__|	859	|__|	860	|__|
Checked cord	861	|__|	862	|__|	863	|__|
Advised breastfeeding	864	|__|	865	|__|	866	|__|
Observed breastfeeding	867	|__|	868	|__|	869	|__|
Advised skin-to-skin contact/warmth	870	|__|	871	|__|	872	|__|
Checked baby for danger signs (including sepsis)	873	|__|	874	|__|	875	|__|
Educated on danger signs	876	|__|	877	|__|	878	|__|
Referred to a health facility	879	|__|	880	|__|	881	|__|
Provided information on washing hands before touching baby	882	|__|	883	|__|	884	|__|
Advised keeping the cord clean	885	|__|	886	|__|	887	|__|
Advised not to bathe the baby within 24 hours after birth	888	|__|				

889	Can you tell us whether or not you were satisfied with the immediate newborn care?<br>**Do not read list of options**	**1 = Yes was satisfied**<br>**2 = No was not satisfied (GO to 891)**<br>**3 = Neither satisfied nor dissatisfied (Go to 892)**	**|___|**

890	**IF YES,** then what was the level of satisfaction<br>**Read both options**	**1 = Fully satisfied Go to 892**<br>**2 = Somewhat satisfied Go to 892**	|___|
891	**IF NO,** then what was the level of dissatisfaction<br>**Read both options**	**1 = Fully dissatisfied**<br>**2 = Somewhat dissatisfied**	|___|
892	**The last time you gave birth, did you keep your newborn at home for several days or weeks without taking the baby out?**	**1 = Yes**<br>**2 = No – GO to 894**	|___|
893	**IF YES, what is the number of days that you kept your newborn in the house?**	**Enter number of days**<br>**Enter 999 if not known**	|___||___||___|
894	**The last time you gave birth, how many days passed before you had visitors come to your house to see the baby? This includes visitors for any reason: health care workers, extended family, or friends.**	**Enter number of days**<br>**Enter 999 if not known**	|___||___||___|
895	**The last time you gave birth, how many days passed before someone other than you had physical contact with the baby? Physical contact means any kind of touching, even if the person did not hold the baby.**	**Enter number of days**<br>**Enter 999 if not known**	|___||___||___|

9. Care of sick newborns

900	**Interviewer – stop to check: was the baby born after September 2004] Ethiopian calendar?**<br><br>**If yes, continue with interview.**<br><br>**If no, end interview at this point and continue with other women aged 13-49 in this household.**	**1 = Yes**<br>**2 = No-End interview**	|__|

Interviewer*: Now I want to talk to you about any sickness [CHILD NAME] experienced in the first 28 days of life*

901	Has [CHILD NAME] ever been sick during first 28 days of life?	**1 = Yes**<br>**2 = No**	|__|

Can I just check, has [CHILD NAME] ever had any of the following symptoms during the first 28 days of life?

Read list of symptoms out loud

**Enter 1 if Yes and continue along row**<br><br>**Enter 2 if No and go to next symptom (row)**<br><br>**If no symptoms, go to Section 10**	**Age at first episode**<br><br>**Enter age in number of days**	**Was care sought for?**<br><br>**1 = Yes**<br>**2 = No**	**If YES, who did you seek care from?**<br><br>**1 = Health post**<br>**2 = Health centre**<br>**3 = Hospital**<br>**4 = HDA**<br>**5 = Traditional Healers**<br>**6 = Pharmacy**	**If care was sought, who cared for (CHILD NAME?)**<br><br>**1 = HEW**<br>**2 = Nurse**<br>**3 = Health Officer**<br>**4 = Pharmacist**<br>**5 = Doctor**<br>**6 = Traditional healer**<br>**7 = Other**	**If care was not sought, why not?**<br><br>**1 = Expected him/her to get better**<br>**2 = Health facility too far**<br>**3 = Cost of treatment too expensive**<br>**4 = Don't trust the facility**<br>**5 = Family member**

<table>
<tr><td colspan="3"></td><td colspan="2"></td><td colspan="2"></td><td colspan="2"></td><td colspan="2"></td><td colspan="2">didn't allow
6 = Community advisor advised against it
7 = Other</td></tr>
<tr><td>Reduced feeding</td><td>902</td><td>|__|</td><td>903</td><td>|__||__|</td><td>904</td><td>|__|</td><td>905</td><td>|__|</td><td>906</td><td>|__|</td><td>907</td><td>|__|</td></tr>
<tr><td>Child was underweight</td><td>908</td><td>|__|</td><td>909</td><td>|__||__</td><td>910</td><td>|__|</td><td>911</td><td>|__|</td><td>912</td><td>|__|</td><td>913</td><td>|__|</td></tr>
<tr><td>Difficult or fast breathing</td><td>914</td><td>|__|</td><td>915</td><td>|__||__|</td><td>916</td><td>|__|</td><td>917</td><td>|__|</td><td>918</td><td>|__|</td><td>919</td><td>|__|</td></tr>
<tr><td>Chest in-drawing</td><td>920</td><td>|__|</td><td>921</td><td>|__||__|</td><td>922</td><td>|__|</td><td>923</td><td>|__|</td><td>924</td><td>|__|</td><td>925</td><td>|__|</td></tr>
<tr><td>Unusually hot or cold</td><td>926</td><td>|__|</td><td>927</td><td>|__||__|</td><td>928</td><td>|__|</td><td>929</td><td>|__|</td><td>930</td><td>|__|</td><td>931</td><td>|__|</td></tr>
<tr><td>Less active than usual</td><td>932</td><td>|__|</td><td>933</td><td>|__||__|</td><td>934</td><td>|__|</td><td>935</td><td>|__|</td><td>936</td><td>|__|</td><td>937</td><td>|__|</td></tr>
<tr><td>Yellow palms/soles/eyes</td><td>938</td><td>|__|</td><td>939</td><td>|__||__|</td><td>940</td><td>|__|</td><td>941</td><td>|__|</td><td>942</td><td>|__|</td><td>943</td><td>|__|</td></tr>
<tr><td>Had diarrhea</td><td>944</td><td>|__|</td><td>945</td><td>|__||__|</td><td>946</td><td>|__|</td><td>947</td><td>|__|</td><td>948</td><td>|__|</td><td>949</td><td>|__|</td></tr>
<tr><td>Convulsions</td><td>950</td><td>|__|</td><td>951</td><td>|__||__|</td><td>952</td><td>|__|</td><td>953</td><td>|__|</td><td>954</td><td>|__|</td><td>955</td><td>|__|</td></tr>
<tr><td>Skin pustules</td><td>956</td><td>|__|</td><td>957</td><td>|__||__|</td><td>958</td><td>|__|</td><td>959</td><td>|__|</td><td>960</td><td>|__|</td><td>961</td><td>|__|</td></tr>
<tr><td>Cord red or draining puss</td><td>962</td><td>|__|</td><td>963</td><td>|__||__|</td><td>964</td><td>|__|</td><td>965</td><td>|__|</td><td>966</td><td>|__|</td><td>967</td><td>|__|</td></tr>
<tr><td>Other (specify)</td><td>968</td><td>|__|</td><td>969</td><td>|__||__|</td><td>970</td><td>|__|</td><td>971</td><td>|__|</td><td>972</td><td>|__|</td><td>973</td><td>|__|</td></tr>
<tr><td>Specify
__________</td><td colspan="12"></td></tr>
</table>

<table>
<tr><td colspan="4">Interviewer: fill in questions 974-983 only if in the above table the interviewee mentioned that care was sought out for any one of the symptoms mentioned</td></tr>
<tr><td>974</td><td>If any of the above symptoms occurred
After how many days did you seek care the first time?</td><td>Write number of days from the onset of illness; if first day of illness, write 0; If possible use the medical record to confirm

99 if care not sought</td><td>|__|__|</td></tr>
<tr><td>975</td><td>Was the [CHILD NAME] diagnosed with very severe disease by a health worker?
Interviewer: list the signs for very sever disease for the mother</td><td>1 = Yes
2 = No</td><td>|__|</td></tr>
<tr><td>976</td><td>Was [CHILD NAME] prescribed medicine for his/her illness?</td><td>1 = Yes
2 = No</td><td>|__|</td></tr>
<tr><td>977</td><td>Did [CHILD NAME] receive 7 consecutive days of gentamycin injection?</td><td>1 = Yes
2 = No</td><td>|__|</td></tr>
<tr><td>978</td><td>Did [CHILD NAME] receive 7 consecutive days of Amoxicillin?

Interviewer: explain Amoxicillin as an oral tablet that</td><td>1 = Yes
2 = No</td><td>|__|</td></tr>
</table>

	**is dissolved and taken as a liquid.**		
979	At any time during the illness, did [CHILD NAME] take any drugs for the illness?	**1 = Yes**<br>**2 = No**	|__|
980	Was [CHILD NAME] managed with expressed breast milk?	**1 = Yes**<br>**2 = No**	|__|
981	Can you tell us whether or not you were satisfied with the care for the sick newborn?<br>**Do not read list of options**	**1 = Yes was satisfied**<br>**2 = No was not satisfied (GO to 983)**<br>**3 = Neither satisfied nor dissatisfied (Go to Section 10)**	|___|
982	**IF YES**, then what was the level of satisfaction<br>**Read both options**	**1 = Fully satisfied (Go to Section 10)**<br>**2 = Somewhat satisfied (Go to Section 10)**	|___|
983	**IF NO**, then what was the level of dissatisfaction<br>**Read both options**	**1 = Fully dissatisfied**<br>**2 = Somewhat dissatisfied**	|___|

Section 10. Children no longer alive (<u>and died before reaching 28 days</u>)

Interviewer: if child is no longer <u>alive and died before reaching 28 days</u>, ask the following questions around symptoms, care-seeking, and cause of death

I understand that it is not easy to talk about children who have died and so please do let me know if you need time to answer the questions. This information is important and will allow the government can develop programs to improve children's health.

Which symptoms did you observe in (CHILD NAME'S) immediately before death?<br><br>**Select all that apply**	For each: 1 = Yes 2 = No		
	1000	Difficult or fast breathing	|___|
	1001	Chest in-drawing	|___|
	1002	Unusually hot or cold	|___|
	1003	Less active than usual	|___|
	1004	Yellow palms/soles/eyes	|___|
	1005	Had diarrhea	|___|
	1006	Convulsions	|___|
	1007	Yellow palms/soles/eyes	|___|
	1008	Skin pustules	|___|
	1009	Cord red or draining puss	|___|
	1010	Other	|___|
1011	If OTHER, please specify	Specify ________________________	

1012	If CHILD NAME died due to an illness, was care sought for that illness?	**1 = Yes**<br>**2 = No–go to end of questionnaire**	
1013	If yes, who provided that care?<br>Put **9 if not known**	**1 = HDA**<br>**2 = HEW**<br>**3 = Nurse**<br>**4 = Doctor**<br>**5 = Health Officer**<br>**6 = Pharmacist**<br>**7 = Other**	**|__|**
1014	If care was sought before [CHILD NAME] died, what was the cause of death diagnosed by the health providers	**1 = Pneumonia**<br>**2 = Diarrhea**<br>**3 = Severe infections**<br>**4 = Other (Specify)___**	
1015	If OTHER	**(Specify)______________________**	

Interviewer: Please double check:

1. Is there another woman aged 13-49 in this household who has not been interviewed?

2. Is there another woman in this household who has been pregnant between [DATE] and [DATE] and has not been interviewed?

(3) Is there another woman in this household who has given birth between [DATE and [DATE] and has not been interviewed?

(4) Is there another woman with a newborn who has not been interviewed

If yes to any of them, please make sure you interview them

If No, end of interview – thank the participant for their time.
