# Supplementary material for: Coverage of antenatal, intrapartum, and newborn care in 104 districts of Ethiopia: A before and after study four years after the launch of the national Community-Based Newborn Care programme
Source: PLoS One. 2021 Aug 5;16(8):e0251706. doi: 10.1371/journal.pone.0251706 (PMC8341496; doi:10.1371/journal.pone.0251706)
Supplement: S3 File — (PDF) [file pone.0251706.s003.pdf]

UNKAA ITTIN GAAFFI QORANNOO
KUNUUNSA DAA'IMMAN HAARAA DHALATANII
KAN HAWWAASA BU'UREEFFATE

Moojuulii 1

**KUTAA 1: Ibsaa Waa'ee Teessoo fi Haala Jireenya Maatii**		
100	Guyyaa	|__|__| / |__|__| / |__|__|__|__|
101	Naannoo	|__|__|__|__|__|__|__|__|__|__|__|__|__|__|__|__|
102	Godina	|__|__|__|__|__|__|__|__|__|__|__|__|__|__|__|__|__|__|__|__|__|__|
103	Maqaa Aanaa	|__|__|__|__|__|__|__|__|__|__|__|__|__|__|__|__|__|__|__|__|__|__|
104	Maqaa PHCU (maqaa buufataa Faayyaa)	|__|__|__|__|__|__|__|__|__|__|__|__|__|__|__|__|__|__|__|__|__|__|
105	Maqaa Gandaa (kebele)	|__|__|__|__|__|__|__|__|__|__|__|__|__|__|__|__|__|__|__|__|__|__|
106	Maqaa Garee	|__|__|__|__|__|__|__|__|__|__|__|__|__|__|__|__|__|__|__|__|__|__|
107	Lakkoofsa Klaastarii<br>**Haa Galmaa'u**	|__|__|__|
108	Lakkoofsa (koodii) Maatii<br>**Haa Galmaa'u**	|__|__|
109	Lakk.Eenyummaa Addaa Maatii (HH ID)<br>**Sanadoota Hunda Irratti Kan Garagalfamu**<br>**Fkn:** Unkaa Odeeffannoo Hirmaattootaa	|__|__|__| / |__|__| Kilaasterii(Zoonii) Maatii (HH)
110	GPS Latitude<br>Laatitiwudii GPS (kan manaa)	|__|__| : |__|__|__|__|__|
111	GPS Longitude<br>Loongitiwudii GPS(kan manaa)	|__|__| : |__|__|__|__|__|
112	Maqaa Abbaa/Haadha Warraa	|__|__|__|__|__|__|__|__|__|__|__|__|__|__|__|__|__|__||__|__|__|__|__|__|__|__|
113	Guyyaa Daawwiin Jalqabaa	Guyyaa |__|__| Ji'a |__|__| Bara |__|__|__|__|
114	**Maqaa Nama Gaaffi Dhiheessu/itu**	Maqaa ______________________________

115	***Gaafataa፡ Unkaan odeeffannoo Hirmaataa dubbifameefi jiraa?***	1 = Eeyyen<br>2 = Lakki	|__|
116	***Gaafataa፡ Gaafatamaan waligaleeraa***	1 = Eeyyen<br>2 = Lakki________________________________<br>*Gaafachu dhiisuun gara abbaa warraa biroo deemi*	|__|
117	Sabummaan abbaa/haadha warraa maatiichaa maali?	1 = Agaw<br>2 = Amaara<br>3 = Beench<br>4 = Burjii<br>5 = Dizzi<br>6 = Geedi'oo<br>7 = Guraagee<br>8 = Haadiyaa<br>9 = Kafficho<br>10= Kambaataa<br>11 = Konta<br>12 = Mee'innit<br>13 = Oromoo<br>14= Silxee<br>15= Tigraay<br>16= Walaayitaa<br>17 = Lammii /Sablammii Ittoophiyaa kan Biro<br>18 = Saba/Sablammii lama/lama ol irraa	|__|__|

	Daawwii	1^{ffaa}	2^{ffaa}	3^{ffaa}	Daawwii Dhumaa
Guyyaa (**Guyyaa/Ji'a/Bara)**		|__|__| / |__|__| / |__|__|__|__|	|__|__| / |__|__| / |__|__|__|__|	|__|__| / |__|__| / |__|__|__|__|	Guyyaa|__|__| Ji'a |__|__| Bara|__|__|__|__| <br>Bu'aa daawwii |__|
**Maqaa Gaafataa/Gaafattu**					
**Bu'aa (kodii barbaachisaa tahe armaan gaditti galchi)**		**|__|**	**|__|**	**|__|**	
Gafiin guyyaa kanatti yoo hin xumuramne yeroon beellama itti aanuu yoomi? **(Guyyaa/yeroo beellamaa)**	Guyyaa beellamaa **(Guyyaa/Ji'a/Bara)**		|__|__| / |__|__| / |__|__|__|__|	|__|__| / |__|__| / |__|__|__|__|	
	**Sa'aatii beellamaa**				

Kodii bu'aa daawwii:

1. Guutamee dhumeera

2. Hanga tokko guutameera

3. Kan dabarfame (beellamaan)

4. Mana keessatti kan hin argamne

5. Gaaffiicha deebisuu kan hin dandeenye

6. Deebii kennuuf kan hin haayyamne /hin feene

Yoo didaan gaafii asiratti dhaabii

118 **Tarreeffama Maatii**

Waa’ee Jiraattoota mana kanaa gaafachuun barbaada. Jiraattoota yammuun jadhu namoota Ji’a 3 fi issan ol mana kana keessa jiraatan fi kan wajjin nyaattan jechuu dha.

Isin dabalate , maqaa jiraattoota mana kanaa gaafachuu danda’aa? (Abbaa/Haadha warraa irraa jalqabee akkaataa tartiiba umrii isaaniitiin guddaa irraa gara xiqqaatti maqaa isaanii naaf tarreessaa)

<table>
<tr><th rowspan="2">Lakk. Tarree</th><th>Maqaa</th><th>Saala</th><th>Guyyaa Dhalootaa Guyyaa/Ji’a/Bara</th><th>Umrii</th><th>Dubartoota Waggaa 13-49 Keessatti Haammataman</th><th>Haala Fuudhaa fi Heerumaa</th><th>Yeroo Barnoota idilee itti raawwatan</th><th>Amantii</th></tr>
<tr><th>Maqaan Haa Galmaa’u</th><th>1 = Dhiira
2 =Dhalaa</th><th>(Guyyaa/Ji’a/Bara)
Yoo hin beekamne, guyyaaf 99 barreessi; yoo hin beekne ji’aaf 99 barreessi; yoo hin beekne baraaf 9999 barreessi.</th><th>Umrii guutuu waggaadhan barreessii
Yoo umriin isaani waggaa tokko gadi ta’e 0 barreessii</th><th>1 = Eeyyen
2 = Lakki</th><th>1 =Kan Fuudhe/heerumte
2 = Kan hin fuune/heerumne garu kan wajiin jiraatan
3 = Kan hin fuune/heerumne
4 = Kan addaan bahan
5 =Kan du’aan addaan bahan
6=hin ilaalatuu</th><th>Baayyinni Waggoota Barnoonni idilee kessa turanii haa ibsamu</th><th>1 = Ortodoksii
2 = Kaatolikii
3 = Proteestaantii
4 = Islaama
5 = Kan Biro
6=hin ilaalatuu</th></tr>
<tr><td>1</td><td>|__|__|__|__|__|__|__|__|__|__|__|__|__|__|_</td><td>|__|</td><td>|__|__|/|__|__|/|__|__|__|__|</td><td>|__|__|</td><td>|__|</td><td>|__|</td><td>|__|__|</td><td>|__|</td></tr>
<tr><td>2</td><td>|__|__|__|__|__|__|__|__|__|__|__|__|__|__|</td><td>|__|</td><td>|__|__|/|__|__|/|__|__|__|__|</td><td>|__||__|</td><td>|__|</td><td>|__|</td><td>|__|__|</td><td>|__|</td></tr>
<tr><td>3</td><td>|__|__|__|__|__|__|__|__|__|__|__|__|__|__|</td><td>|__|</td><td>|__|__|/|__|__|/|__|__|__|__|</td><td>|__|__|</td><td>|__|</td><td>|__|</td><td>|__|__|</td><td>|__|</td></tr>
<tr><td>4</td><td>|__|__|__|__|__|__|__|__|__|__|__|__|__|__|</td><td>|__|</td><td>|__|__|/|__|__|/|__|__|__|__|</td><td>|__|__|</td><td>|__|</td><td>|__|</td><td>|__|__|</td><td>|__|</td></tr>
<tr><td>5</td><td>|__|__|__|__|__|__|__|__|__|__|__|__|__|__|</td><td>|__|</td><td>|__|__|/|__|__|/|__|__|__|__|</td><td>|__|__|</td><td>|__|</td><td>|__|</td><td>|__|__|</td><td>|__|</td></tr>
<tr><td>6</td><td>|__|__|__|__|__|__|__|__|__|__|__|__|__|__|</td><td>|__|</td><td>|__|__|/|__|__|/|__|__|__|__|</td><td>|__|__|</td><td>|__|</td><td>|__|</td><td>|__|__|</td><td>|__|</td></tr>
<tr><td>7</td><td>|__|__|__|__|__|__|__|__|__|__|__|__|__|__|</td><td>|__|</td><td>|__|__|/|__|__|/|__|__|__|__|</td><td>|__|__|</td><td>|__|</td><td>|__|</td><td>|__|__|</td><td>|__|</td></tr>
<tr><td>8</td><td>|__|__|__|__|__|__|__|__|__|__|__|__|__|__|</td><td>|__|</td><td>|__|__|/|__|__|/|__|__|__|__|</td><td>|__|__|</td><td>|__|</td><td>|__|</td><td>|__|__|</td><td>|__|</td></tr>
<tr><td>9</td><td>|__|__|__|__|__|__|__|__|__|__|__|__|__|__|</td><td>|__|</td><td>|__|__|/|__|__|/|__|__|__|__|</td><td>|__|__|</td><td>|__|</td><td>|__|</td><td>|__|__|</td><td>|__|</td></tr>
<tr><td>10</td><td>|__|__|__|__|__|__|__|__|__|__|__|__|__|__|</td><td>|__|</td><td>|__|__|/|__|__|/|__|__|__|__|</td><td>|__|__|</td><td>|__|</td><td>|__|</td><td>|__|__|</td><td>|__|</td></tr>
</table>

Amma waa'ee haala jireenya maatii kanaa gaafachuun barbaada.<br><br>***Hubachiisaa Gaafataadhaaf: Kutaa kanaafi kutaalee hafan hundumaaf, filaannnowan hundumaa tokko tokkon dubbiisiif, yoo filannoo hin dubbisiin jedhame malee.***			
119	Girgiddaan/dhaabnii manaa irra caalaan maal irraa hojjatame**?**	1 = Girgiddaa hin qabu<br>2 = Meeshaa uumamaa, shambaqqoo/agadaa, muka, dhoqqee<br>3 = Dhagaa fi dhoqqe<br>4 = Dhagaa/bolookeeta fi simintoo<br>5 = Kan biroo	|__|
120	Irra caalaa lafti mana keessaa maal irraa hojjatame**?**	1 =<br>Waan uumamaa /lafa /cirracha/falti/<br>2 = Waan salphaa (muka/zanbaabaa/ leemana)<br>3 = Waan qophaa'ee dhume (muka xaawlaa/afata laastikaa/ xuubii shaklaa/siminto afame/<br>4 = Kan biroo	|__|
121	Gubbaan manaa irra caalaa maal irraa hojjatame**?**	1 = Cuqqaalli citaa/baalaa<br>2 = Qorqorroo/xuubii<br>3 =Kan biroo	|__|
122	Manni fincaanii maatiin kun itti fayyadamu gosa kami**?**	1 = Hin qaban/bosana/dirree<br>2 = Boolla taa'umsa qabu<br>3 = Kan bishaan itti dhanga'lau	|__|
123	**Maatiin kun bishaan dhugaatii itti fayyadamu irra caalaatti eessa irraa argata?**<br><br>**Filannoowan hin dubbisiin**	1. Ujummoo (tubboo) kallattiin gara manaa keessaatti diriire<br>2. Ujummo (tubboo) kallattiin gara mooraatti diriire<br>3. Ujummoo (tubboo) naannoo (kan walinii)<br>4. Boolla bishaanii<br>5. Boolla bishaanii eeggamu<br>6. Madda eeggamu<br>7. Bishaan roobaa cimmisuun<br>8. Bishaan dachee (laga, haroo, haroo xiqqa namaan qotame)<br>9. Boolla banaa tahe<br>10. Madda hin eeggamne<br>11. Raabsaa bishani irraa<br>12. Bishaan	|__|

		lastikaan/qaruuraan qadaadame omishamu<br>13. Taankarii	
124	Bishaan dhugaatiif akka mija'aa tahu ykn qulqulleessuuf waanti gootan jiraa?	1 = Eeyyen<br>2 = Lakki - gara kutaa 127 darbi<br>3 = Hin beeku - gara kutaa 127 darbi	|__|
125	Waanti gootan yoo jiraate, irra caalaa wantii isiin gootan maali?<br>**Hin dubbisin**	1 = Xuriin anga jalatti kuufamuuttii eeggu<br>2 = Carqi/hucuu dhaan xalalchu/dhinbiibuu<br>3 = Xalalchituu bishaanii fayyadamuun (Biyyee/cirracha/kompostii/kkf)<br>4 = Danfisuun<br>5 = Ifa aduutiin danfisuu<br>6 = Mala ammayyaa bishaan ittiin walaanan /kloorinii itti naquun<br>7 = Kan biroo, haa ibsamu<br>____________________<br>8 = Hin Beeku	|__|
126	**Kan biroo yoo jiraate haa ibsamu**	Haa Ibsamu______________________	
127	Maatiin kun, nyaata bilcheessuuf madda humnaa yeroo baay'ee gosa kamitti fayyadama?<br><br>**Hin dubbisiin**	1 = Kosii<br>2 = Muka bobeessaa<br>3 = Cilee guraachaa/kasala<br>4 = Buttaa gaazii<br>5 = Laambaa/gaazii<br>6 = Eleektirika<br>7 = Kan biroo	|__|
128	Maatiin kun humna eleektirikaa ni qaba?	1 = Eeyyen<br>2 = Lakki	|__|

Maatiin kun meeshaalee tarreeffaman keessaa isaan kam fi ammam qaba?<br><br>**Kan namootni maatii keessa jiraatan hundi qaban hida'i**		**Baay'innii lakkoofsaa haa barraahuu: (yoo hin jiranne 0 Barreessi)**	
	129	Sa'aatii harkaa	|__|
	130	Warqii/ziqeeya giraamaan	|__|
	131	Ifaa laambaa/ ifaa humna ukkaamameen argamu	|__|
	132	Siree	|__|
	133	Bilbila manaa/kan haadaa	|__|

	134	Bilbila moobaayilii		|__|
	135	Biskileeta/saaykilii		|__|
	136	Konkoolaata		|__|
	137	Raadiyoona		|__|
	138	Teelevizhiina		|__|
	139	Firijii/qorriisiistuu		|__|
140	Mannii keessaa jiraattan kun kan dhuunfaa keessaniiti?		1 = **Eeyyen**<br>2 = **Lakki**	|__|
141	Maatii keessaa namni qabeenya lafa qonnaa qabu jiraa?		1 = **Eeyyen**<br>2 = **Lakki gara 143 deemii**	|__|
142	Miseensoonni maatii kanaa walitti qabaatti lafa qonnaa heektaara meeqa qaba**?**		Bal'ina W/G heektaaraan agarsiisi (Tokko gad yoo tahe hiraan agarsiisi; fkn: 0.5) yoo heektarrii hin beekamnee 99 barreessi	|__||__.||__|
143	Maatiin kun beeladoota kan akka horii, lukkuu ykn beeladoota tajaajila qonnaa kennan qaba?		1 = Eeyyen<br>2 = Lakki, gara kuta 2 deemii	|__|

Kan armaan gaditti tarreeffaman keessaa maatiin kun gosa beeladootaa isaan kam fi ammam qaba**?**	**Tokkoon tokkoon isaaniitif lakkoofsa barreessii,yoo homaa hin jirree 0 barrressii**		
	144	Lukku/Indaaqqo	|__|__|__|
	145	Re'ee	|__|__|__|
	146	Hoolaa	|__|__|__|
	147	Harree	|__|__|__|
	148	Farda	|__|__|__|
	149	Gaangee	|__|__|__|
	150	Gaala	|__|__|__|
	151	Horii aananii	|__|__|__|
	152	Qotiyoo/sangaa	|__|__|__|

Yoo dubartiin umriin 13-49 tahe Tarree Maatii (Gaafii 118) keessaa hin jirree gaafii xumurii.

Moojuulii 2

Kutaa 2. Dubartoota Qorannoo Kana Keessaatti Hammatamu Danda'an

Gaafataa : Gaafiiwan kanaan gadii dubartoota umriin isaanii 13-49 tahan hudaaf dhihaachu qabu. Dubartoota umriin isaanii 13-49 tahan kan Tartiba Maatii (Gaafii 118) kessaatti galmeefaman hundi gaafatamuu qabu. Dubartoota umriin 13-49 kessaa ishee angafaatiin gaafii jalqabi.

Gara gaafiitti darbuu kee dura , duraan dursamee unkaan odeeffannoo hirmaattootaa guutamu qaba (Hanga 204)

Gaafiicha xumuruun yoo hin danda'amne, sababni isaa ibsamee beellamni haa qabamu

Yeroo Daawwii	**1ffaa**	**2ffaa**	**3ffaa**
**Guyyaa/Ji'a/Bara**	|__|__| / |__|__| / |__|__|__|__|	|__|__| / |__|__| / |__|__|__|__|	|__|__| / |__|__| / |__|__|__|__|
**Maqaa Gaafataa**			
**Bu'aa (Koodiin sirrii tahe haag uutamu)**	**|__|**	**|__|**	**|__|**
**Gaafiiin guyyaa kana yoo xumuramu baate, Guyyaa Beellamaa itti aanu (Guyyaa/Ji'a/Bara)**		|__|__| / |__|__| / |__|__|__|__|	|__|__| / |__|__| / |__|__|__|__|
**Gaafataa : Gaafiiin guyyaa kana yoo xumuramu baate , sa'aati beellamaa itti aanu yoom taha (Beellama fudhu) ?**		__|__| : __|__	__|__ : |__|__

Kodii Bu'aa
1. Kan guutame
2. Kan hanga tokko Guutame
3. Yeroo biraaf kan dabarfame
4. Mana keessatti kan hin argamne
5. Gaaficha deebisuuf kan hin dandeenye
6. Deebisuuf kan hin haayyamne/kan didan

Marsaa 3ffaa deebi'uudhaan gaafiicha xumuru yoo hin dandeenye, gara dubartii biroo maati kana keessa jirtu ykn gara maatii birootti darbi.yoo danda'amee qophaa qophaatti gaafichaa gaafachuu yaali.

200	**Gaafataa: Maqaa dubartii ishee jalqabaa (tokkoffaa) galmeessi**	|__|__|__|__|__|__|__|__|__|__|__|__|__|__|__|__|__|__|__|__|
201	**Gaafataa:** Lakk. eenyumaa /kodii addaa dubartii kanaa galmeessi **Lakk. Kodii ulfaa Kodii Klaastarii = Gaf.107**	|__|__|__| Klaastarii; |__|__| Maatii; |__|__| Dubartii

	**Kodii Maatii = Gaf.108**<br>**Kodii Dubarti = Gaf.118**		
202	**Maqaa Gaafataa/Gaafattuu**	Maqaa______________________________	
203	**Gaafataa:** Unkaan odeeffannoo hirmaataa dubbifameefi jira?	1 = Eeyyen<br>2 = Lakki	|__|
204	**Gaafataa: Dubartiin kun waligaltee jirti? Lakki yoo tahe, gaafii kee asumaatti dhaabii. Dubartoota biro miseensa maati kanaa umriin isaanii 13-49 tahan gaafadhu. Yoo jiraatan Unkaa Gaaffii biroo kutaa 2 irraa jalqabii itti fuuffii.**	1 = Eeyyen<br>2 = Lakki	|__|
**Hirmaannaa keetiif si galatoomfadha, amma waa'ee Raayyaa Misooma Fayyaa si gaafachuun barbaada.**			
205	**Ati Raayyaa Misooma Fayyaa keessatti miseensa? (1:5 hoggantuudha)?**	1 = Eeyyen<br>2 = Lakki	|__|
206	**Ati Raayyaa Misooma Fayyaa keessatti haadha garee dha? (1:30)**	1 = Eeyyen<br>2 = Lakki	|__|

Amma waa'ee ulfaa (garaatti baachuu) si gaafachuun barbaada.			
207	Yeroo ammaa ulfa garaa qabdaa?	1 = Eeyyen<br>2 = Lakki	|__|
208	Kanaan dura ulfa taatee (garaatti baattee) beektaa?	1 = Eeyyen<br>2 = Lakki (Gaaficha dhaabi)	|__|
209	Kanaan dura ilmo deesse (dhalte) beektaa?	1 = Eeyyen<br>2 = Lakki	|__|
210	Waligalatti ijoolle meeqa deesse (dhalte)?	Lakkoofsa barreessii	|__|
211	Waa'ee daa'iman lubuun isaani darbite ykn ulfa karatti bade dubbachuun akka nama dhibu ykn akka ulfaatu nin beeka. Garu rakkoole si mudatan hunda yoo nutti himte mootummaan fayyaa haadholee fi daa'immanii kunuunsuuf tattaaffii godhu foyyeessuuf gargaara.<br><br>Ulfi bade (karatti hafe) si mudatee beekaa?	1 = Eeyyen<br>2 = Lakki (Gara 213)	
212	Ulfa meeqaatu bade ykn lubuun hin dhalanne?	Lakkoofsa Galchi	
213	Amma waa'ee ulfa Fulbaana 2004 irraa jalqabee turan si gaafadha.<br>Fulbaana 2004 as ulfoofte beektaa?	1 = Eeyyen<br>2 = Lakki (Gaaficha dhaabi)	|__|

Amma waa'ee ulfa Fulbaana 2004 booda turan si gaafachuun barbaada. Yammuun ulfa unduma jedhu ulfoota kan lubbuun dhalatan, kan lubbun hin dhalanne ykn kan badan fi kan idda dhalatanii du'anis yoo jiratan dabalate jechuu kooti. Akkasumas kan amma gara biraa jiratanis hunda dabalata.

Gaafata:a) (a) duraan dursii kaardiin dhaloota daa'imani yoo jirate gaafadhu. (b) ulfa ykn dahumsa isa dhiyoo irraa calqabii anga Fulbaana 2004tti deebi'un ulfa ykn dahumsa hundaa gaafadhu. Yoo isheen yeroo amma ulfaa taatee ulfa isa yeroo kana garaa jiru itti hin dabaliin. Ulfa raawwate duwwa lakkaawi.

Gaafata: Yoo lakkuu deesse tokkoon tokkoonsanii qobaa qoobaatti galmeessi (koodii adda baasu qobaa qobaatti kenniif). Akkasumas, yoo Fuulbaana 2004 as ijoollee tokko ol deesses hundumaa qobaa qobaatti galmeessi (koodii adda baasus qobaa qobaatti kenniif).

Yeroo guutuu dura jechuun yoo daa'imni osoo yeroon da'umsaa hin geennee dhalate, osoo haadha ciniinsuun hin qabne jechuudha.

Lakk. adda baasaa ulfaa (Pregnancy ID)	Bu'aa Ulfaa	Maqaa Daa'imaa	Guyyaa dhalootaaa/guyyaa ulfii raawatamee	Lakku tahanii kan dhalatan	Saala	Ammaa lubbuun jiraa/ jirtii?	Yoo lubbuun jiran hanga ji'a darbe umriin meeqa tahaa?	Yoo lubbuun darbe, yoom?
Ulfa yeroo dhihoo turee irraa jalqabi	1 = Lubbuun kan dhalate/tte<br>2 = Lubbun kan hin dhalatin<br>3= ulfa ji'a 9 ffaa dura bade	Maqaan yoo hin baaneef, hin baaneef jadhi guuti	Guyyaan yoo hin beekamne 01 galchi<br>Kan guyyaa hin beekneef Ji'a/bara akka siif himan isaan jajjabeesi	1 = Eeyyen<br>2 = Laki<br>3=hin beekuu	1 =Dhiira<br>2 = Dhalaa<br>3= hin beekuu	1 =Eeyyen<br>2 =Lakki	Guyyoota 28 gad yoo tahe guyyootaan barreessi	Guyyaan yoo hin beekamne 01 galchi<br>Kan guyyaa hin beekneef Ji'a/bara akka siif himan isaan jajjabeesi
1	|__|		Guyyaa |__|__| Ji'a |__|__| Bara|__|__|__|__|	|__|	|__|	|__|	Guyyaa |__|__| Ji'a|__|__|	Guyyaa |__|__| Ji'a |__|__| Bara|__|__|__|__|
2	|__|		Guyyaa |__|__| Ji'a |__|__| Bara|__|__|__|__|	|__|	|__|	|__|	Guyyaa |__|__| Ji'a|__|__|	Guyyaa |__|__| Ji'a |__|__| Bara|__|__|__|__|
3	|__|		Guyyaa |__|__| Ji'a |__|__| Bara|__|__|__|__|	|__|	|__|	|__|	Guyyaa |__|__| Ji'a|__|__|	Guyyaa |__|__| Ji'a |__|__| Bara|__|__|__|__|

4	|__|		Guyyaa |__|__| Ji'a |__|__| Bara|__|__|__|__|	|__|	|__|	|__|	Guyyaa |__|__| Ji'a|__|__|	Guyyaa |__|__| Ji'a |__|__| Bara|__|__|__|__|
5	|__|		Guyyaa |__|__| Ji'a |__|__| Bara|__|__|__|__|	|__|	|__|	|__|	Guyyaa |__|__| Ji'a|__|__|	Guyyaa |__|__| Ji'a |__|__| Bara|__|__|__|__|
6	|__|		Guyyaa |__|__| Ji'a |__|__| Bara|__|__|__|__|	|__|	|__|	|__|	Guyyaa |__|__| Ji'a|__|__|	Guyyaa |__|__| Ji'a |__|__| Bara|__|__|__|__|

Akka amma naaf himteen Fulbaana 2004 irraa jalqabee ulfa turan mirkaneessuun barbaada.			
214	Ida'aama lakkoofsa daa'iman lubbuun dhalatanii = |__|	1 = Eeyyen<br>2 = Lakki	|__|
215	Ida'aama lakkoofsa daa'iman du'anii dhalatanii = |__|	1 = Eeyyen<br>2 = Lakki	|__|
216	Baay'innaa ulfaa yeroo malee badanii = |__|	1 =Eeyyen<br>2 =Lakki	|__|

Yaadachiisa: Odeefannoon ida'aamnii yoo sirri dhufuu baatan , gaafataan dubarti gaafachuun adda baasee sirreessu qaba

Fulbaana 2004 irraa jalqabee ulfi yoo jiraate gaaficha itti fufi.

Moojuulii 3

**KUTAA 3. Lakkoofsa Ulfaa (Pregnancy ID)**			
**Gaafataa: Odeefannoo waa'ee daa'imaa gabatee galmee ulfaa (kanaan ol jiru) irraa fudhadhu.**			
300	Maqaa daa'imaa lubbun dhalatee	☐☐☐☐☐☐☐☐☐☐☐☐ Yoo daa'imni du'ee dhalatee 99 barreessii	
301	**Lakk. Kodii ulfaa**<br>**Kodii Klaastarii = Gaf.107**<br>**Kodii Maatii = Gaf.108**<br>**Kodii Dubarti = Gaf.118**<br>**Koodii ulfaa = Gabatee galmee ulfaa) guuti.**	|__|__|__| Klaastarii |__|__| Maatii |__|__| Dubarti |__|__| Ulfaa	
**KUTAA 4. Kununsa Dahumsaa Duraa**			
**Gaafataa፡ Gaafiilee kanaan gadii maqa'aa daa'imaa caqasii gaafadhu. Bu'aan ulfaa kan du'ee dhalate ykn ulfa bade yoo tahe gaafilee irratti bakka maqaa daa'imaa lakkoofsaa ulfaa fayyadami.**<br><br>**Amma waa'ee ulfa (maqaa daa'imaa/lakk ulfaa) sigaafachuun barbaada**<br><br>**RMF- Raayyaa Misooma Fayyaa**<br>**REF-Raayyaa Ekisteenshinii Fayyaa**			
400	Yeroo **(Maqaa daa'imaa/lakk. ulfa) ulfoofte sana ulfaa'uu kee maatii kee ala namoota birootiif himte ture**?	1 = Eeyyen<br>2 = Lakki (Gara 404 deemi)	|__|
401	Ogeessoota fayyaatiif himte yoo ture, yeroo jalqabaa eenyuuf himte?	1 = RMF<br>2 = REF<br>3 = Ogeessoota fayyaa kan biro (Narsii)<br>4 = Kan biroo	|__|
402	Kan biroo (Haa Ibsamu)	Haa Ibsamu ______________________________	
403	Ulfaa'uu kee RMF tti/REFtti/oggeessa fayyaa kan birootiif yammu himtu ulfa ji'a meeqaa turte?	Lakkoofsa torbaanii barreessi yoo hin beekamne 99 barreessi	|__|__|
404	Kaardii Faayyaa Maatii kan odeeffannoo ulfa fi da'umsaa qabuu ni qabda?<br><br>**Gaafataa:** Gosa kaardiichaa agarsiisi gaafadhu	1 = Eeyyen<br>2 = Lakki (Gara 406 Deemi)	|__|

405	**Eeyyen yoo tahe:** Kardii fayyaa maatii keessan ilaalu danda'aa?<br><br>**Gaafataa:** kaardiin fayyaa hadhoolii fi daa'imanii ni jiraa**?** **Yoo lakkii** ta'ee gara gaafii kunuunsaa da'umsaa duraattii (ANC) darbi.	1 = Eeyyen<br>2 = Lakki	|__|
406	Yeroo ulfa (maqaa daa'imaa/lakk ulfa) hordooffi **ulfaa ykn kununsa duhmsa duraa gootani ture**?<br><br>**Haa Ibsamu: kununsa dahumsa duraa Kellaa Fayyaa, Buufata Fayyaa irraa ykn REF/RMF tiin mana isaanii keessatti ilaalamani jiru? Yoo lakkii ta'ee gaafilee waa'ee dahumsa duraa darbi?**	1 = Eeyyen<br>2 = Lakki (Gara kutaa 6 darbi)	|__|

Eeyyen yoo tahe, daawwii kunuunsa dahumsa duraatiif (ANC) eessa dhaqtanii ture? (Hunda agarsiisi)<br>**Filaannoowan deebii'an hundumaa filadhu**	Tokko tokkof: 1 = Eeyyen 2 = Lakki		
	407	Mana	|__|
	408	Keellaa Fayyaa	|__|
	409	Buufata Fayyaa	|__|
	410	Hospitaala	|__|
	411	Kan biro	|__|
	412	(Haa Ibsamu) ____________________	

**Kunuunsa Dahumsaa Duraa Keellaa Fayyaa irraa**			
413	Kellaa Fayyaa irraa kunuunsa dahumsa duraa/haadholee ulfaa argattee beektaa?	1 = Eeyyen<br>2 = Lakki Yoo tahe Gara 422 darbi	|__|
414	Yeroo ulfaa keeti kunuunsa dahumsa duraa/haadholee ulfaa Keellaa Fayyaa irraa yeroo meeqa argatee?	Yeroo meeqa akka tahe galmeessi;	|__|__|

		Kan hin beekamne yoo tahe 99 Barreessi	
415	Keellaa Fayyaatti yeroo duraa tiif kan ilaalamte yoom ture?<br>Odeefanno kana yoojiraate Kardii Fayyaa haadholee fi daa'immanii irraa guuti?	Guyyaa |__|__| Ji'a |__|__| Bara |__|__|__|__| Yoo guyyaan hin beekmanee 99 barreessii	
416	**Gaafataa: Ragaan haadha irraa moo kardii irraa argame**	1 = Haadha irraa<br>2 =Kardii Irraa	|__|
417	Yeroo duraatiif yammuu ilaalamtu ulfa torbaan meeqaa turte?	Lakkoofsa Torbanii galmeessi<br>Yoo hin beeknee 99 barreeessii	|__||__|
418	**Gaafataaf:Yeroo kunuunsaa da'umsaa duraa yammu ilaalamtu yeroon ulfaa kardii irra yoo jiraatee lakkoofsa isaa barreessii**	Lakkoofsa Torbanii galmeessi<br>Yoo lakkoofsaa hinbedeknee 99 barreessii	|__||__|
419	Kunuunsa dahumsaa duraa Keellaa Fayyaa irraa argattetti quuftee moo itti hin quufne<br>**Filannoowwan hin dubbisin**	**1 =** Eeyyen itti quufeera<br>**2 =** Lakki itti hin quufne<br>**(Gara 421 deemi)**<br>**3 = Itti quufeeras itti hin quufnes jechuu hin danda'u**<br>**(Gara 422 Deemi)**	|___|
420	Eeyyeen yoo tahe, ammam ittin quufte**?**<br>**Filannoowwan lachuu dubbisi**	1 = Guutuu guutuutti itti quufeera (Gara 422 Deemi)<br><br>2 = Hanga tokko itti quufeera<br>(Gara 422 Deemi)	|___|
421	Lakki yoo tahe , ammam itti hin quufne?<br>**Filannoowwan lachuu dubbisi**	1 = Guutuu guutuutti itti hin quufne<br>2 = Hanga tokko itti hin quufne	|___|

**Kunuunsa Dahumsaa Duraa Buufata Fayyaa Keessatti**			
422	Buufata fayyaa keessatti kunuunsa dahumsaa duraa argatte jiraa?	**1** = Eeyyen<br>2 = Lakki **(Gara 434 Darbi)**	|__|
423	Kunuunsa dahuumsa duraatiif yeroo jalqabaa kan ilaalamte buufata fayyaa keessatti ture?	**1** = Eeyyen<br>2 = Lakki	|__|
424	Buufata fayyaatti yeroo meeqaaf kunuunsa dahuumsa duraa tiif ilaalamte?	**Baayina Yeroo barreessi**<br>**Yoo hin beeknee 99**	|__||__|

		**barreessii**	
425	Buufata fayyaatti yeroo duraa tiif kan ilaalamte yoom ture?<br>**Gaafataa : Guyyaa, Ji'a fi Bara akka himan jajjabeessu yaali. Yoo guyyaa yaadechuu baatte, ji'a fi bara galmeessi**	Guyyaa |__|__| Ji'a |__|__| Bara |__|__|__|__| **Yoo hin beeknee 99 barreesssi**	
426	Gaafataa፡ Kaardiin Hordoffii Fayyaa haadholee fi daa'imanii yoo jiraate isa irraa ilaaali galmeessi	Guyyaa |__|__| Ji'a |__|__| Bara |__|__|__|__| yoo odeefannoon kaardii faayyaa maatii irraa hin argamnee 99/999/9999 tarbii isaatiin guuti.	
427	Yeroo duraatiif yammuu ilaalamtu ulfa yeroo meeqaa turte?<br>Gaafataa፤ Kaardiin Hordoffii Fayyaa haadholee fi daa'imanii yoo jiraate isa irraa ilaaali galmeessi	Baayyina Torbanii galmeessi<br>Yoo hin beekne ta'e 99 barreessi	|__||__|
428	**Gaafataa: Ragaan haadha irraa moo kardii irraa argame**	1 = Haadha irraa<br>2 = Kardii irraa	|__|
429	Yeroo jalqabaa yammu ilaalamtu, eenyuutu si ilaale?	1 = Narsii<br>2 = Deessiftuu<br>3 = Qondaala Fayyaa(HO)<br>4 = Kan biro (haa ibsamu)<br>5=hin beeku	|__|
430	**Kan biro yoo tahe (Haa Ibsamu)**	Haa Ibsamu__________________	
431	Kunuunsa dahumsaa duraa Buufata Fayyaa irraa argattetti quuftee moo itti hin quufne?<br><br>**Filannoowwan hin dubbisin**	**1 = Eeyyen itti quufeera**<br>**2 = Lakki itti hin quufne**<br>(Gara 433 deemi)<br>**3 = Itti quufeeras itti hin quufnes jechuu hin danda'u**<br>(Gara 434 Deemi)	|___|
432	Eeyyeen yoo tahe, ammam itti quufte**?**<br><br>**Filannoowwan lachuu dubbisi**	**1 = Guutuu guutuutti itti quufeera (gara 434 deemii)**<br><br>**2 = Hanga tokko itti quufeera (gara 434 deeemii)**	|___|
433	Lakki yoo tahe, ammam itti hin quufne?<br>**Filannoowwan lachuu dubbisi**	**1 = Guutuu guutuutti itti hin quufne**<br>**2 = Hanga tokko itti hin quufne**	|___|

**Kunuunsa Dahumsaa Duraa Mana Keessatti**			
434	Oggeessa fayyaa irraa hordoffiin kunuunsa dahumsaa duraa mana keessan keessatti isinii godhamee jiraa?	1 = Eeyyen<br>2 = Lakki (**gara 445 deemii**)	|__|
435	Yeroo ulfa keetii yeroo meeqaaf ogeessi fayyaatiin mana kee keessatti ilaallamte?	**Baayyina yeroo Galchi**	|__|
436	Yeroo jalqabaatiif mana keessatti kan ilaalamte yoom ture?<br><br>**Gaafataa፤** Kaardiin fayyaa haadholee fi daa'immanii yoo jiraate isa irraa galmeessi	Guyyaa |__|__| Ji'a |__|__| Bara |__|__|__|__|__|	

437	**Ragaan kun haadha irraa moo kardii irraa argame**	**1** = Haadha irraa<br>2 = Kaardii irraa	|__|
438	Yeroo duraatiif manatti yammu ilaalamtu ulfa torban meeqaa turte?	Baayyina torbanii galmeessi	|__||__|
439	Gaafataa: Kaardiin Hordoffii Fayyaa haadholee fi daa'imanii yoo jiraate isa irraa ilaaali galmeessi	Baayyina torbanii galmeessi	|__||__|
440	Yeroo jalqabaaf si ilaaluuf gara mana keeti kan dhufe eenyu ture?	**1** = REF<br>2 = RMF<br>3 = Kan biro	|__|
441	Kan biro (Haa Ibsamu)	Haa Ibsamu___________________________	
442	Kunuunsa dahumsaa duraa mana kee keessatti argattetti quuftee moo itti hin quufne?<br>**Filannoowwan hin dubbisin**	1 = Eeyyen itti quufeera<br>2 = Lakki itti hin quufne<br>(Gara 444 deemi)<br>3 = Itti quufeeras itti hin quufnes jechuu hin danda'u<br>(Gara 445 Deemi)	|___|
443	Eeyyeen yoo tahe, ammam itti quufte**?**<br>**Filannoowwan lachuu dubbisi**	1 = Guutuu guutuutti itti quufeera (gara 445 deemii)<br><br>2 = Hanga tokko itti quufeera (gara 445 deeemii)	|___|
444	Lakki yoo tahe , ammam itti hin quufne?<br>**Filannoowwan lachuu dubbisi**	1 = Guutuu guutuutti itti hin quufne<br>2 = Hanga tokko itti hin quufne	|___|

Amma yeroo kunuunsi ulfaa isiniif keennamu eenyuu akka isiniif keenneen isiin gaafadha.<br>*Gaafaata:* **Du'ee kan dhalatee/ulfaa bade yoo tahe, bakka maqaa daa'imaa lakkoofsa kodii dahumsaa/ulfa fayyadamii.**<br><br>*Daa'ima kana (Maqaa daa'imaa .lakkoofsa kodii) yeroo ulfooftan tajaajilawwan armaan gaditti argaman isiniif keennamani ture?* **(Gaafataa፡-Kunis kunuunsa dahumsa duraa kan Mana keessa, Keellaa Fayyaatti fi Buufata Fayyaatti tajaajila keenname tahu danda'aa.)**			
445	Ulfina (Kiloograamaan) saffaramtani ture?	1 = Eeyyen<br>2 = Lakki(gara 448 deemii)	|__|
446	Ogeessi fayyaa isiin ilaale kam ture**?**<br>**Yoo hin beekamnee 9 barreessii**	1 = RMF<br>2 = REF<br>3 = Narsi/Deessiftu<br>4 = Qondaala Fayyaa<br>5 = Dooktara<br>6 = Kan biro	|__|
447	Tajaajila kana iddoo kamitti argattan?	1 = Mana<br>2 = Keellaa Fayyaa<br>3 = Buufata Fayyaa<br>4 = Hospitaala<br>5 = Kan biro	|__|
448	Dheerinni kee saffarame ture?	1 = Eeyyen<br>2 = Lakki(gara 451 deemii)	|__|
449	Eeyyen yoo tahe, tajaajila kana yeroo duraatiif kan siif kenne isa kami dha?<br>**Yoo hin beekamnee 9 barreessii**	1 = RMF<br>2 = REF<br>3 = Narsi/Deessiftu<br>4 = Qondaala Fayyaa<br>5 = Dooktara<br>6 = Kan biro	|__|
450	Tajaajila kana iddoo kamitti argattan?	1 = Mana<br>2 = Keellaa Fayyaa<br>3 = Buufata Fayyaa<br>4 = Hospitaala<br>5 = Kan biro	|__|
451	Waa'ee harma hoosissuu oddeeffaannoo argatanii beektuu?	**1** = Eeyyen<br>2 = Lakki **(gara 454deemii)**	|__|
452	Eeyyen yoo tahe, tajaajila kana yeroo duraatiif kan siif **k**enn**e** isa kami dha?<br>**Yoo hin beekamnee 9 barreessii**	1 = RMF<br>2 = REF<br>3 = Narsi/Deessiftu<br>4 = Qondaala Fayyaa<br>5 = Dooktara<br>6 = Kan biro	|__|
453	Tajaajila kana iddoo kamitti argattan?	1 = Mana	|__|

		2 = Keellaa Fayyaa<br>3 = Buufata Fayyaa<br>4 = Hospitaala<br>5 = Kan biro	
454	Dhibbaan dhigaa isiniif saffarame/ilaalame ture?<br>**Haa Ibsamu, saffartuu harka gubbaa irratti maranii ittiin ilaalani**	**1 =** Eeyyen<br>2 = Lakki **(gara 457 deemii)**	|__|
455	**Eeyyen Yoo tahe, tajaajila kana yeroo duraatiif kan siif kenne isa kami dha?**<br>**Yoo hin beekamnee 9 barreessii**	1 = RMF<br>2 = REF<br>3 = Narsi/Deessiftu<br>4 = Qondaala Fayyaa<br>5 = Dooktara<br>6 = Kan biro	|__|
456	Tajaajila kana iddoo kamitti argattan?	1 = Mana<br>2 = Keellaa Fayyaa<br>3 = Buufata Fayyaa<br>4 = Hospitaala<br>5 = Kan biro	|__|
457	Qorannoon fincaanii sii godhamee turee?	**1 = Eeyyen**<br>**2 = Lakki(gara 460)**	|__|
458	Eeyyen yoo tahe, tajaajila kana yeroo duraatiif kan siif kenne isa kami dha?<br>**Yoo hin beekamnee 9 barreessii**	1 = RMF<br>2 = REF<br>3 = Narsi/Deessiftu<br>4 = Qondaala Fayyaa<br>5 = Dooktara<br>6 = Kan biro	|__|
459	Tajaajila kana iddoo kamitti argattan?	1 = Mana<br>2 = Keellaa Fayyaa<br>3 = Buufata Fayyaa<br>4 = Hospitaala<br>5 = Kan biro	|__|
460	Qorannoo dhukkubba fantoo/ falxaataa/wurdee siif godhanii ture?	1 = Eeyyen<br>2 = Lakki(**gara 463 deemii**)<br>3= hin beekuu(**gara 463 deemii**)	|__|
461	Eeyyen yoo tahe, tajaajila kana yeroo duraatiif kan siif kenne isa kami dha?<br>**Yoo hin beekamnee 9 barreessii**	1 = RMF<br>2 = REF<br>3 = Narsi/Deessiftu<br>4 = Qondaala Fayyaa<br>5 = Dooktara<br>6 = Kan biro	|__|
462	Tajaajila kana iddoo kamitti argattan?	1 = Mana<br>2 = Keellaa Fayyaa<br>3 = Buufata Fayyaa<br>4 = Hospitaala<br>5 = Kan biro	|__|

463	Albuuda sibila qaamaa keessaa yabbisuuf (iron folate jedhamu) kiniina diimaa ykn shirooppi fudhatte ture?<br>**Kininicha ykn fakkii agarsiisii**	1 = Eeyyen<br>2 = Lakki **(gara 467 deemii)**	|__|
464	**Eeyyen yoo tahe,** tajaajila kana yeroo duraatiif kan siif kenne isa kami dha?<br>**Yoo hin beekamnee 9 barreessii**	1 = RMF<br>2 = REF<br>3 = Narsi/Deessiftu<br>4 = Qondaala Fayyaa<br>5 = Dooktara<br>6 = Kan biro	|__|
465	Tajaajila kana iddoo kamitti argattan?	1 = Mana<br>2 = Keellaa Fayyaa<br>3 = Buufata Fayyaa<br>4 = Hospitaala<br>5 = Kan biro	|__|
466	**Yoo fudhatte,** guyyoota meeqaaf kiniina/shirooppi fudhatte?	Bayyina guyyootaa barreessi Yoo hin yaadanne 999 Barreessi	|__||__||__|
467	Kitibaata arka ykn irree irrati kennamu kan daa'imni akka dhibee teetaanas jedhamuun hin qabamne kan dhoowwu si waraananii turee?<br><br>**Qajeelfamaa dirree irratti gaafataan fakki ykn fakeenya (sample) agarsiisuu isaani mirkaneessi**	1 = Eeyyen<br>2 = Lakki **(gara 473 deemii)**	|__|
468	Eeyyeen yoo ta'e kitibaata titaanas yeroo meeqa waraanamte?	**Yeroo isaa barreessi**	|__||__|
469	Lamaa gadi yoo ta'e, ulfa kana duraanoo kitibaata tiitanas waraannatteettaa?	**1=** Eeeyyeen<br>2= Lakkii**,gara 473 deemii**	||__|
470	Yoo 469 eyyeen ta'e, ulfa kana duraan yeroo meeqa kitibaata tiitanas waraannatte?	**Baay'innaa yeroo barreessii**<br>**Yoo hin beeknee 99 barreessii**	|__||__|
471	Yoo 469 eeyyeen ta'ee, ulfaa kana duraa waggaa meeqa ta'a erga teetanasii warannamtee?	**Baay'innaaa waggaa barreessii**<br>**Yoo hin beeknee 99 barreesssii**	|__|__|
472	Tajaajila kana iddoo kamitti argattee?	1 = Mana<br>2 = Keellaa Fayyaa<br>3 = Buufata Fayyaa<br>4 = Hospitaala<br>5 = Kan biro	|__|
473	Waa'ee HIV oddeeffannoo ykn barnoota argatte turtee?	1 = Eeyyen<br>2 = Lakki, gara 476 deemii	|__|
474	Tajaajilicha yeroo jalqabaaf eenyu irraa argatte?<br><br>**Yoo hin beekamnee 9 barreessii**	1 = RMF<br>2 = REF<br>3 = Narsii/Deessiftuu<br>4 = Qondaala Fayyaa<br>5 = Dooktoota<br>6 = Kan biro	|__|

475	Iddo Kamitti tajaajilicha Argatte?	1 = Mana<br>2 = Keellaa Fayyaa<br>3 = Buufata Fayyaa<br>4 = Hospitaala<br>5 = Kan biro	|__|
476	Qorannoo HIV argattee turtee?	1 = Eeyyen<br>2 = Lakki (gara 479 deemii)	|__|
477	Tajaajila kana yeroo jalqabaaf eenyu irraa argatte?<br>**Yoo hin beekamnee 9 barreessii**	1 = RMF<br>2 = REF<br>3 = Narsii/Deessiftuu<br>4 = Qondaala Fayyaa<br>5 = Dooktoota<br>6 = Kan biro	|__|
478	Iddo kamitti tajaajilicha argatte?	1 = Mana<br>2 = Kellaa Fayyaa<br>3 = Buufata Fayyaa<br>4 = Hospitaala<br>5 = Kan biro	|__|
479	Waa'ee qorannoo STI (dhukkuboota walqunamtii saalaatiin dadarban) argattee turee?	1 = Eeyyen<br>2 = Lakki(gara 482 deemii)	|__|
480	Tajaajila kana yeroo jalqabaaf eenyu irraa argatte?<br>**Yoo hin beekamnee 9 barreessii**	1 = RMF<br>2 = REF<br>3 = Narsii/Deessiftuu<br>4 = Qondaala Fayyaa<br>5 = Dooktoota<br>6 = Kan biro	|__||__|
481	Iddo kamitti tajaajilicha argatte?	1 = Eeyyen<br>2 = Lakki	|__|
482	Yeroo ulfa keetii yeroo waldhaansa dhukkuba STI (dhukkuboota walqunamti saalaatin dadarban) argatte ture?	1 = Eeyyeen<br>2 = Lakkii (gara 485 deemii)	|__||__|
483	Tajaajilicha jalqabaaf eenyu irraa argatte?<br>**Yoo hin beekamnee 9 barreessii**	1 = RMF<br>2 = REF<br>3 = Narsii/Deessiftuu<br>4 = Qondaala Fayyaa<br>5 = Dooktoota<br>6 = Kan biro	|__|
484	Dhaabbata fayyaa kamitti tajaajilicha argattan?	1 = Mana<br>2 = Keellaa Fayyaa<br>3 = Buufata Fayyaa<br>4 = Hospitaala<br>5 = Kan biro	|__|
485	Yeroo ulfa keetii sana waa'ee sirna nyaataa odeeffanno ykn barumsa argatte ture?	1 = Eeyyen<br>2 = Lakki(gara 488 deemii)<br>3= hin barbaachisuu (gara 488 deemii)	|__|
486	Tajaajilicha yeroo jalqabaaf eenyu irraa argatte?<br>**Yoo hin beekamnee 9 barreessii**	1 = RMF<br>2 = REF<br>3 = Narsii/Deessiftuu<br>4 = Qondaala Fayyaa<br>5 = Dooktoota<br>6 = Kan biro	|__|

487	Iddo Kamitti tajaajilicha argatte?	1 = Mana<br>2 = Kellaa Fayyaa<br>3 = Buufata Fayyaa<br>4 = Hospitaala<br>5 = Kan biro	|__|
488	Yeroo ulfa keetii waa'ee mallattowwan dhukkuba cimoo yeroo ulfaa dhufuu danda'anii odeeffanno ykn barumsa argatte ture?	1 = Eeyyen<br>2 = Lakki(gara 491 deemii)	|__|
489	Tajaajila kana yeroo jalqabaaf eenyu irraa argatte?<br>**Yoo hin beekamnee 9 barreessii**	1 = RMF<br>2 = REF<br>3 = Narsii/Deessiftuu<br>4 = Qondaala Fayyaa<br>5 = Dooktoota<br>6 = Kan biro	|__|
490	Iddo kamitti tajaajilicha argatte?	1 = Mana<br>2 = Kellaa Fayyaa<br>3 = Buufata Fayyaa<br>4 = Hospitaala<br>5 = Kan biro	|__|
491	Waa'ee qophiilee dahumsaa godhaman fi rakkoolee dahumsa waliin walqabatanii dufuu danda'an akkaataa salphisuuf gorsa argattaniittuu?<br>**Haa Ibsamu,** Maallaqaan , Gargaarsa yeroo deessu, Geejiiba	1 = Eeyyen<br>2 = Lakki(gara 501 deemii)	|__|
492	Tajaajilicha yeroo jalqabaaf eenyu irraa argatte?<br>**Yoo hin beekamnee 9 barreessii**	1 = RMF<br>2 = REF<br>3 = Narsii/Deessiftuu<br>4 = Qondaala Fayyaa<br>5 = Dooktoota<br>6 = Kan biro	|__|
493	Iddo kamitti tajaajilicha argatte?	1 = Mana<br>2 = Keellaa Fayyaa<br>3 = Buufata Fayyaa<br>4 = Hospitaala<br>5 = Kan biro	|__|
494	Qophii dahumsaa duraa fi rakkoolee dahumsa waliin walqabatanii dufuu danda'an ilaalchisee adeemsii karooraa galmaa'e argamaa?	1 = Eeyyen<br>2 = Lakki(gara 501deemii)	|__|
495	Tajaajilicha yeroo jalqabaaf eenyu irraa argatte?<br>**Yoo hin beekamnee 9 barreessii**	1 = RMF<br>2 = REF<br>3 = Narsii/Deessiftuu<br>4 = Qondaala Fayyaa<br>5 = Dooktoota<br>6 = Kan biro	|__|
496	Iddo kamitti tajaajilicha argatte?	1 = Mana<br>2 = Kellaa Fayyaa<br>3 = Buufata Fayyaa<br>4 = Hospitaala<br>5 = Kan biro	|__|

Kutaa 5. kunuunsaa da'umsaa duraa itti fuufaa			
500	Waa'een qophii da'umsaa fi rakkoolee dahumsa waliin walqabatanii dufuu danda'anii kaardii faayyaa maatii irraa ni jira?<br>**Kaardiin Fayyaa Maatii yoo jiraate, odeeffannoo isaa irraa fudhu.**	1 = Eeyyen<br>2 = Lakki<br>3=kaardiin faayyaa maatii hin jiru	|__|

Dubartii hulfaaf mallattoowwan balaa maal maal akka ta'an natti himuu dandeessaa?<br><br>**Gaafataa: Kan Ibsaman qofa barreessi, filaannoo hin dubbisiin. Gaafiin kun waa'ee beekumsa mallatoolee cimooti malee waa'ee muxannoo ishee tii miti.**	**Tokko tokkof: 1 = Eeyyen 2 = Lakki**		
	501	Dhiiga Qaama Hormaataa	|__|
	502	Dhukkuba Garaa Cimaa(Garaa muruu/ciru)	|__|
	503	Dhamgala'aa Gadaameessa irraa yaa'u	|__|
	504	O'a qaamaa	|__|
	505	Dhukkuba mataa ykn ijaan sirritti ilaalu dhabu	|__|
	506	Dagachu/Irraanfachu	|__|
	507	Dhiita'a Harkaa fi Miilaa	|__|

Karoorri fi qophiin dahumsa duraa maal maal of keessaa akka qabatu natti himu dandeessaa?<br><br>Gaafataa: Kan Ibsaman qofa Barreessi, filaannoo hin dubbisii.<br>**Gaafataa: Kan Ibsaman qofa Barreessi, filaannoo hin dubbisii.kun waa'ee beekumsaa mallatoolee cimooti malee waa'ee muxannoo ishee tii miti.**	Tokkon tokkon gaafiileetiif: 1 = Eeyyen 2 = Lakki		
	508	Maallaqa	|__|
	509	Geejiba	|__|
	510	Nyaata ga'aa	|__|
	511	Yeroo dahumsaa nama kunuunsa godhu	|__|
	512	Iddo itti dahaan/dhalan adda baasanii beekuu	|__|
	513	Barbaachisaa Yoo tahe, namni dhiiga gumaachu/arjoomu jiraachu isaa	|__|
	514	Huccu qulqulluu	|__|
	515	Huccu yeroo dhalan irratti dahan /dhalan	|__|
	516	Guwantii	|__|
	517	Faashaa	|__|
	518	Saamuna	|__|
	519	Haadu/milaaci haaraa	|__|
	520	Maqasa affeelame	|__|
	521	Jibrii/haada kan affeelame	|__|

522	Qophii dahumsaa gootani beektuu?<br><br>**Haa Ibsamu:** Maallaqa, Nama isiin Gargaaguu, Geejiba	**1** = Eeyyen<br>2 = Lakki,gara 539 deemii	|__|

	**Tokko tokkof: 1 = Eeyyen 2 = Lakki**		
	523	Maallaqa	|__|
	524	Geejiba	|__|
	525	Nyaata Ga'aa	|__|
	526	Yeroo dahumsaa nama kunuunsa godhu	|__|
	527	Iddo itti dahaan/dhalan adda baasanii beekuu	|__|
	528	Barbaachisaa Yoo tahe, namni dhiiga gumaachu/arjoomu jiraachu isaa	|__|
Eeyyen yoo tahe, dahumsa keetiif qophii maali goote?<br><br>**Kan armatti tarreeffaman osso hin ibsiniif kan siif deebisan barreessi**	529	Huccu Qulqullu	|__|
	530	Huccu yeroo dahan/dhalan itti uwwissan	|__|
	531	Guwantii	|__|
	532	Faashaa /	|__|
	533	Saamuna	|__|
	534	Haadu/milaaci Haaraa	|__|
	535	Maqasa affeelaman	|__|
	536	JIbrii/haada affeelame kan hodhaaf ta'u	|__|
	537	Kan biroo	|__|
	538	Kan biro yoo jiraate Haa Ibsamu ____________________________	

539	Yeroo ulfa keetii, walgahii dubartoota ulfaa irratti hirmaattee ture?	1 = Eeyyen<br>2 = Lakki **(gara 552deemii)**	|__|
540	Eeyyen yoo tahe, walgahii dubartoota ulfoo irratti yeroo meeqaaf hirmaatte?	**Baayyina isaa barreessii**	|__|

	**Tokko tokkof: 1 = Eeyyen 2 = Lakki**		
	541	Qophii dahumsaa	|__|
Walgahii dubartoota ulfoo kana irratti, kan armatti tarreeffaman keessaa kam irratti marii'atame?	542	Barbaachisummaa kunuunsa dahumsaa duraa	|__|
	543	Dhaabbata fayyaa keessatti dahuu/dhaluu	|__|
	544	Barbaachisummaa hordoffi dahumsaa boodaa	|__|
	545	Kunuunsa daa'imman haaraa dhalatan	|__|
	546	Kan biroo	|__|

	547	Haa Ibsamu_____________________________|	
548	Waa’ee walgahii dubartoota ulfaa eenyutu sitti hime?	**1** = RMF<br>2 = HEF<br>**3 =** Kan biroo (Haa Ibsamu**)**	|__|
549	Kan biro yoo jiraate, haa ibsamu.	**Haa Ibsamu___________________**	
550	Walgahiin dubartoota ulfa eessatti gaggeefame?	1= RMF<br>2= Iddo walgahii Gooxii/Araddaa<br>3= Keellaa Fayyaa<br>4= Buufata Fayyaa<br>5= Kan biro___________	
551	Kan biro yoo jiraate, haa ibsamu.	**Haa Ibsamu___________________**	
552	Yeroo ulfa keetii kunuunsa argattetti quuftee moo hin quufne?<br><br>Tarreeffama Fillannoo hin dubbisiin	**1 =** Eeyyen itti quufeera<br>2 = Lakki itti hin quufne (Gara 554 deemi)<br>3 = Itti quufeeras itti hin quufnes jechuu hin danda’u (Gara 555 Deemi)	|___|
553	**Eeyyen yoo tahe, ammam itti quufte?**<br>Filannowwan lamaan dubbisi	1 = Guutuu guutuutti itti quufeera (gara 555 deemii)<br><br>2 = Hanga tokko itti quufeera (gara 555 deeemii)	|___|
554	Yoo**, itti hin quufne ta’e ammam itti hin quufne?**<br>Filannowwan lamaan dubbisi	1 = Guutuu guutuutti itti hin quufne<br>2 = Hanga tokko itti hin quufne	|___|

**Gaafataa:**<br>Yoo gaafatamtuun kunuunsaa da'umsaa duraa (ANC) dhaabata fayyaatti kan argatte tahe gaafilee dabalata (waa'ee ANC) armaan gadi gaafadhu			
	**Tokkoon tokkon gaafileetiif:1=eeyyeen 2=lakkii**		
Yoo dhaabbaataa faayyaattii qorannoo faayyaa ulfaan wal qabatu adeemsiiftuu, kannneen armaan gadii keessaa maal maal maal hubattan/taajabdan?<br><br>**Fillaannoowan debiin hundumaa filadhu**	555	gaafii waa'ee da'umsaa akka gaafattuu sii jaajjabeesuu	|___|
	556	Filannoo siif keennuu akka filannoo kaamiyyuu filattuu (fkn guyyaa da'umsaa, seeraan ykn da'umsaa baqaqsaanii da'uu fkkf.)	|___|
	557	Kaka'umsaa hin taane gochuun akka kutaa –c fudhaattu ykn garaa baqaqsaan akka deessu sii taasisuu	|___|
	558	Adeemsaa kam innii/isheen akka fayyaadamtuu ibsaa siif keennu (fkn. da'umsaa uumamaa ykn kutaa –C ykn yeroo ammam akka fudhattuu ibsuu)	|___|
	559	Yeroo qorannoo mirgaa qophummaa kee siif eeguu ykn sitti huwisuu (fkn.maggaarajaa/daddeesituu)	|___|
	560	Yeroo qorannoo Afaan kabaja hin qabnee fayyadamuu (fkn.arrabaa fi ykn jecha sii /maatii/hawaasaa/saba kee xiqqeessuu jechuu)	|___|
	561	Doctorrii muxannoo qabu/hojii ijoo isaa siif dhiyeessuu dhiisuu ykn tajaajilaa seeraan siif keennuu dhiisuu sababa ati saba/hawasaa tokko keessaa dhufteef	|___|
	562	Yeroo qorannoo sagalee hamaa baasuu ykn siittii iyyuu	|___|
	563	Yeroo qorannoo afaan hin taaneen fayyaadamuu ykn si abaaruu	|___|
	564	Si sodaachisuu fkn yoo ati kana didde garaa si baqaqsa ykn opareeshinii goona jechuun	|___|
	565	Yeroo qorannoo qophaakee si gatanii deemuu ykn si irranfachuu	|___|
	566	Yeroo qorannoo bu'aa/gabasa qorannoo faayyaa sii wajjiin hasawuu/ibsaa siif laatuu	|___|
	567	Bakka namni biro jirutti ykn haala namni biro salphaan dhaga'utti bu'aa qorannoo kee ibsuu	|___|
	568	Odeefannoon qorannoo faayyaa keetii/galmeen kee akka nama biraatti hin mul'anne waadaa sii galuu	|___|
	569	Tajaajila ati gaafatte tokko qarshii ykn maallaqa hin qabdu jechuun si dhoowachuu ykn siif kennuu dhiisuu	|___|

Yoo bu'aan ulfaa =3 (ulfa bade yoo tahe) gaafii dhaabiitii ulfi biro yoo jiraate gaafadhu.

6. Kunuunsa dahumsaa

Amma, yeroo dahumsaa (maqaa daa'imaa/lakkoofsa ulfaa) fi haalawwan dahumsaa booda turan irratti gaafilee Muraasa si gaafachuun barbaada.

600	Eessatti deesse/dhalte?	1 = Mana (gara 602 deemii)<br>2 = Keellaa Fayyaa (gara 609 deemi)<br>3 = Buufata Fayyaa (gara 609 deemi)<br>4 =Hospitaala(gara 609 deemi)<br>5 = Kan biroo	|__|
601	Kan biro yoo jiraate, haa ibsamu.	Haa ibsamu________________________________|	

Mana jireenyaa keessatti yoo deesse/dhalte, maaliif?<br><br>**Kaan deebiisan hunda filaadhuu**	Tokkon Tokkoon gaafileetiif : 1 = Eeyyen 2 = Lakki		
	602	Hoggayyu mana keessa waaniin da'uuf	|__|
	603	Abbaan manaa/haati abbaa manaa kiyyaa waan hin haayyamneef	|__|
	604	Tajaajila fayyaa waanin hin feeneef	|__|
	605	Tajaajila fayyaa argachuun mi'aa waan taheef	|__|
	606	Sababoota aadaa/amantii tahaaniif	|__|
	607	Kan biroo	|__|
	608	Haa Ibsamu_____________________________________|	
Keellaa Fayyaa keessattii yoo deessee, maaliif?<br><br>**Kaan deebisan hunda filaadhuu**	tokkon tokkoon gaafiileetiif : 1 = Eeyyen 2 = Lakki		
	609	Hoggayyu dhaabbata fayyaa keessa waaniin dahuuf/dhaluuf	|__|
	610	Sababa rakkoo hulfa ykn dahumsa waliin walqabate tureen	|__|
	611	REF/RMF tiin eergameen	|__|
	612	Walgahii dubartoota ulfoo irratti gorsa waaniin argadheef	|__|
	613	Cimimmuun waan narra tureef ykn rakkoo yeroo dahumsaa uumameen	|__|
	614	Dhaabbata fayyaatti dahuun mijja'aa waan taheef	|__|
	615	Baasiin isaa xiqqaa waan taheef	|__|
	616	Kan biroo haa ibsamu	|__|
	617	Haa ibsamu_____________________________________|	

618	Irra caalaa kan sideessise eenyu turee?		1 = Dooktara<br>2 = Narsii/deessiftuu<br>3 = REF<br>4 = RMF<br>5 = Deessiftuu aadaa<br>6 = Fira/hiriyaa<br>7 = Namu<br>8 = Kan biro	|__|
	Deesisuu irratti kan biroo eenyutu hirmaate?<br>**Kan illaallatu hunda filadhu.**	619	Dooktara	|__|
		620	Narsii/deessiftuu	|__|
		621	REF	|__|
		622	RMF	|__|
		623	Deessiftuu aadaa/gandaa	|__|
		624	Fira/hiriyaa	|__|
		625	Namuu	|__|
		626	Kan biroo	|__|

627	Namni irra caalaa si deessisan yeroo sideesisan harka isaanii saamunaan dhiqatanii turan?	1 = Eeyyen<br>2 = Lakki<br>3 = Hin Beeku	|__|
628	Namoonni si deessisan sun yeroo sii deesisan guwantii harkatti godhatani/uffatanii turee?	1 = Eeyyen<br>2 = Lakki<br>3 = Hin Beeku	|__|
629	Yeroo deesse/dhalte, dahumsii sun iddoo qulqulluu irratti gaggeeffamee? (Iddo qulqulluu jechuun afata qlqulluu)	1 = Eeyyen<br>2 = Lakki<br>3 = Hin Beeku	|__|
630	Dawaan/qorichi dhiigni akka baayyee hin dhangalaane dhoowwu kan 'misoprostol' jedhamu siif keennamee turee?	1 = Eeyyen<br>2 = Lakki<br>3 = Hin Beeku	|__|
631	**Gaafataa:- dahumsi dhaabbata Fayyaa keessatti kan godhame/adeemsiifamee yoo tahe, kan itti aanu gaafadhu. Yoo hin taane Gara 633 darbii**<br><br>Erga deessee/dhaltee booda dhaabbata fayyaa keessa guyyaa meeqaaf turte?	Baayyina guyyootaa barreessi<br><br>Guyyaa dahuumsa isaanii qofa yoo tahe 0 barreessuun darbi	|__|__|
632	(Maqaa Daa'imaa) yammu dhalatu opraasiyooniin (ykn garaa baqaqsanii baasuun) deessee/dhaltee?	1 = Eeyyen<br>2 = Lakki	|__|

	tokkon tokkoon gaafiileetiif = Eeyyen 2 = Lakki		
(Maqaa Daa'imaa) yammu deessan, kanneen tarreeffaman keessaa tokko isiin mudatee ture?<br>**Filannoowaan dubbiisii**	633	Diigini baayyee dhangala'uu	|__|
	634	Ciniinsuu sa'aatii 12 irra caalu	|__|
	635	Of walaaalu	|__|
	636	Ciniinsuu yeroo male dhufe	|__|

	637	Dhangala'aan foolii qabuu bahuu	|__|
	638	Daa'imni taa'umsa hin taaneen dhufuu	|__|

639	Yeroo da'umsaa tajaajila fooyya'aa argachu akka dandeessaniif, gara dhaabbata fayyaa ol'aanaa akka deemtan gorsii isiniif keenname turee?	1 = Eeyyen<br>2 = Lakki(**gara 646 darbii**)	|__|
640	Maaliif gara fayyaa ol'aanaa san akka deemtan goorfamtan?<br><br>(**Gaafataa:- Haalli hamaan kan galmaa'e yoo jiraatee mirkaneessi**)	1 = Sababa rakkoo/balaa cimaa tokko ykn sani ol mul'ateef<br>2 = Hanqina meeshaa/iddoo walaansaa<br>3 = Hanqina oggeessa ga'aa tahe<br>4 = Kan biroo	|__|
641	Kan biro yoo jiraate, Haa Ibsamu_________________________	Haa Ibsamu____________________________	
642	Gara dhaabbata fayyaa ol'aanaa akka deemtan yeroo isiniif himame deemtani turee?	1 = Eeyyen<br>2 = Lakki	|__|
643	Lakki Yoo tahe, Maaliif?	1 = Dhaabbatichi waan fagoo taheef<br>2 = Baasiin heddu waan taheef<br>3 = Gara dhabbilee adda addaati deemu waaniin hin jaalenneef<br>4 = Deemuuf hayyama dhabuun<br>5 = Kunuunsa dhaabbatichi kennu waan hin jaalanneef<br>6 = Kan biroo	|__|
644	Kan biro yoo jiraate, haa ibsamu_________________________	Haa ibsamu____________________________	
645	Ambulaansii argachuu dandeessee turee?	1 = Eeyyen<br>2 = Lakki	|__|
646	Tajaajila dahumsaa isinii keennametti quufte moo itti hin quufne?<br><br>**Gaafataa፡ Filannowwan Hin Dubbisin**	1 = Eeyyen itti quufeera<br><br>2 = Lakki Itti hin quufne **(gara 648 deemi)**<br>3 = Itti quufe ykn itti hin quufne jechu hin danda'u **(gara 649 deemi)**	|___|
647	Deebiin Eeyyen yoo tahe, ammam itti quufte?<br><br>**Gaafataa፡ Filannoowwan Dubbisi**	1 = Guutuu guutuutti itti quufeera (**gara 649 deemii**)<br>2 = Hanga tokko itti quufeera (**gara 649 deemii**)	|___|
648	Deebiin Lakki Yoo tahe, ammam itti hin quufne?<br><br>**Gaafataa፡ Filannoowwan Dubbisi**	1 = Guutuu guutuutti itti hin quufne<br>2 = Hanga tokko itti hin quufne	|___|

Gaafataa: Gaafiiwan kanaan gadii (649-670) kan gaafatamtu dhaabbata fayyaa keessatti kan deesse yoo tahe duwaadha

Yammuu Keellaa Fayyaa, Buufata Fayyaa ykn Hospitaalatti dahuuf dhaqxee achi jirtu sana kanneen keessaa kan si mudate jiraa?<br><br>Deebiwwan keennaman hunda Barreessi	Gaafii tokko tokkoof 1 =Eeyyen 2 = Lakki		
	649	Waa’ee haala keessa jirtu fi hojii walaansaa kana wajjiin walqabatu, namni yeroo yeroon ibsa si keennu ni jira ture?	|___|
	650	Gara kutaa dahumsaatti yaammu si galchan namni si uwwise/hagooge ni jira ture?	|___|
	651	Erga murtiin keennamee booda (ffk opraasiyoonii gochuuf) tajaajilli fayyaa harkifatee ykn ture ni jiraa?	|___|
	652	Jecha kabaja namaa tuqu sitti dubbatanii ture	
	653	Yeroo dahumsaa akka malee qophaa kee si dhiisanii bahanii ture?	|___|
	654	Ossoo sitti hin himne /osso ati hin haayyamin tajaajilli walaansaa (fkk oparaasiyoonaan dhalu, dhiiga si kennu, KKf) siif keenname turee?	|___|
	655	Yeroo dahumsaa dawaa dhukkuba salphisu yeroo gaafattu namni xiyyeeffannaa si doowwate ykn si dide ni jira turee?	|___|
	656	Yeroo dahumsaa namni sitti iyye ykn siin lole ni jira turee?	|___|
	657	Namne kabalaan si dhahe ni jira turee?	|___|
	658	Namni si qunxuuxe ni jira turee?	|___|
	659	Namni si tume ni jira turee?	|___|
	660	Yeroo sigaragalchan namni humnaan si dhiibe ni jira ture?	|___|
Keellaa Fayyaa, Buufata Fayyaa ykn Hospitaalatti erga deesse booda kanneen keessaa kan si mudate jiraa?<br><br>Deebiwwan keennaman hunda filadhu	Tokkon tokkoon gaafiiliitiif: 1 = eeyyee 2 = lakkii		
	661	Erga deesse booda gaafi qabdu akka gaafattu namni si jajjabeesse ni jiraa?	|___|
	662	Erga deessee/dhalte booda namni sirritti si uwwise/hagooge ni jiraa?	|___|
	663	Dawaa dhukkuba salphisu osso gaafattu xiyyeeffannoo/ nama deebi si keennu dhabdee ture?	|___|
	664	Deessee oggusuma kophaa kee dhiifamte turtee?	|___|
	665	Erga deessee/dhaltee booda namni sitti iyye ykn sagalee lolaa sitti fayyadame ni jiraa?	|___|
	666	Erga deessee/dhaltee booda namni jecha kabaja kee tuqu siin jedhe ni jiraa?	|___|
	667	Erga deessee/dhaltee booda siree dahumsaa akka qulqulleessitu namni si gaafate ni jiraa ?	|___|
	668	Erga deessee/dhaltee booda mana fincaanii akka qulqulleessitu namni si gaafate ni jiraa ?	|___|
	669	Kaffaltiin guutuun waan hin kaffalamneef namni dhaabbata fayyaa keessa akk turtu si godhe ni jiraa? (Fkn: Sii fi daa’ima kee hanga kaffaltiin guutuun kaffalamu isiin tursiisuu)	|___|
670	Yoo komii qabaattee bakka dhaqxee itti imattu/iyattu beektaa?	1 = Eeyyen<br>2 = Lakki	|___|

7. Kunuunsa Haadhoolee Dahumsaan Boodaa (PNC)

Amma, Waa'ee Hordoffii Dahuumsaa Boodaa erga deessanii boodaa Gootan isin gaafachuun barbaada.

700	Dahumsaa booda torban 6 keessatti, namni hordoffii fayyaa siif godhe yookin fayyaa kee ilaale jiraa?<br><br>**Qoradhu: Kan fayyaa ishaanii ilaale eenyuu akka ta'e: Hojjattu Exteenshinii Fayyaa, hojjattaa/uu buufata fayyaa, naarsii/Doktoora ta'uu.**	1 = Eeyyen<br>2 = Lakki (gara kutaa 8 deemii)	|__|
701	Hordoffiin fayyaa isa daumsa boodaa inni jalqabaa erga deesse guyaa meeqa booda siif godhame?<br><br>**Gaafataa: Gaafin kun qorannoo faayyaa haadhaa kan ilaallatu ta'u isaa ibsi.**	Baayyinni guyyaa haa ibsamu<br>**Yoo hin beeknee 99 barreessii**	|__|__|
702	Hordoffiin fayyaa inni jalqabaa eessatti siif kenname ?	1 = Mana<br>2 = Kellaa Fayyaa<br>3 = Buufata Fayyaa<br>4 =Hoospitaala<br>5 = Kan biroo	|__|
703	Eenyuun?<br>Yoo hin beekne 9 barreessi	1 = Doktara<br>2 = Narsi/Deessiftu<br>3 = REF<br>4 = Qondaala Fayyaa<br>5 = RMF	|__|
704	Hordoffiin fayyaa inni lammaffaa erga deesse booda siif godhame ture ?	1=eeyyeen<br>2=lakkii **(Gara 712 deemii**)	
705	Hordoffiin fayyaa inni lammaffaa erga deesse guyyaa meeqa booda siif godhame	Baayyinni guyyaa Haa ibsamu<br>**Yoo hin beeknee 99 barreessi**	|__|__|
706	Hordoffiin fayyaa inni lammaffaa eessatti siif kenname?	1 = Mana<br>2 = Kellaa Fayyaa<br>3 = Buufata Fayyaa<br>4 =Hoospitaala<br>5 = Kan biro	|__|
707	Eenyuun?<br>Yoo hin beekne 9 barreessi	1 = Doktara<br>2 = Narsi/Deessiftu<br>3 = REF<br>4 = Qondaala Fayyaa<br>5 = RMF	|__|
708	Hordoffiin fayyaa inni sadaffaa erga deesse booda siif godhame ture ?	1=eeyyee<br>2=Lakkii (gara 712 deemii)	|__|
709	Hordoffiin fayyaa inni sadaffaa erga deesse guyyaa meeqa booda siif godhame	Baayyinni guyyaa Haa ibsamu<br>**Yoo hin beekne 99 barreessii**	|__|__|
710		1 = Mana<br>2 = Kellaa Fayyaa	|__|

	Hordoffiin fayyaa inni lammaffaa eessatti siif kenname?	3 = Buufata Fayyaa<br>4 =Hoospitaala<br>5 = Kan biro	
711	Eenyuun?Yoo hin beekne 99 barressi	1 = Doktara<br>2 = Narsi/Deessiftu<br>3 = REF<br>4 = Qondaala Fayyaa<br>5 = RMF	|__|

Hordoffii dahumsaa boodaa yeroo gootaniin, fayyaa keessan mirkanessssuuf maal maaltuu isiniif godhamee ture?

Gaafataa: Filannowwan kanaan gadii dubbisiif. Deebiiwwaan deebiiwan ykn caqafaman hundaa irraa marsi.

Daawwiin yoo yeroo caqafameetti hin adeemsiifameennee, tarree sana bakka duwaa dhiisii

	Tokkon tokkoon gaafiileetif: 1 =Eeyyen 2 =Lakki					
	(Gaaffi #)	Guyyoota 0-2	(Gaaffi #)	3-7 Guyyoota	(Gaaffi #)	8-42 Guyyoota
Hordoffi/ilaalcha Harmaa	712	|__|	713	|__|	714	|__|
Gorsa Harrma Hoosisuu	715	|__|	716	|__|	717	|__|
Ibsa Mallattoowwan balaa fidanii	718	|__|	719	|__|	720	|__|
Gorsaa fi Tajaajila Karoora Maatii	721	|__|	722	|__|	723	|__|
Hubanno Sirna Nyaataa	724	|__|	725	|__|	726	|__|
Gara Dhaabbata fayyaatti akka deemtee ilaalamtu/rifaarii	727	|__|	728	|__|	729	|__|
Dhiibbaa Dhiigaa Saffaramtani (meeshaa kan arkatti hidhaniin)	730	|__|	731	|__|	732	|__|
Madaa Sababa dahaatiin/dhalatiin dhufe wal'aanamtanii(yoo jiraate)	733	|__|	734	|__|	735	|__|
Kan biroo	736	|__|	737	|__|	738	|__|
Kan biroo_Haa Ibsamu___________	|					

739	Tajaajila dahuumsaa Boodaa siif keennemeen Itti quufte moo itti hin quufne?<br><br>**Gaafataa፡ Kunuunsii kun kan haadhaa ta'u ibsii kan daa'imaa haaraa dhalatee akka hin taanee himiif.Filannoowwan Hin dubbisin**	1 = Eeyyen Itti quufeera<br>2 = Lakki Itti hin quufne **(Gara 741 darbi)**<br>3 = Itti Quufe ykn Itti hin quufne jachuu hin danda'u **(Gara Kutaa 8 darbi)**	|___|
740	Deebiin Eeyyen Yoo tahe, Sadarkaan itti quufta hangam ture??<br>Gaafataa፡ Filannoowwan Dubbisi	1 = Guutu Guutuutti Itti quufeera **(Gara Kutaa 8 darbi)**<br>2 = hamma tokko Itti quufeera**(Gara Kutaa 8 darbi)**	|___|
741	Deebiin Lakki Yoo tahe, Sadarkaan itti hin quufne hangam ture??	1 = Guutu Guutuutti Itti quufeera<br>2 = Hanga tokko Itti quufeera	|___|

Yoo bu’aan ulfaa =2 (du’ee dhalatee) gaafii asumatti dhaabii. Ulfaa biroo mirkaneessii

8. **Daa’imman Haaraa Dhalatan Battalumatti Kunuunsuu**			
**Amma yeroo [maqaa daa’imaa] dhalatuu fi sana booda maal akka tahe si gaafachuun barbaada.**			
800	Gaafataaf : Lakkoofsii ulfaa [Maqaa Daa’imaa] meeqa”	Lakk. Eenyummaa Gabatee irraa Guuti	|__|
801	Tokko qofaatu dhalatee?	1 =Yes<br>2 =Lakki	|__|
802	[CHILD NAME] weighed at birth?<br>[Maqaa daa’immaa] yeroo dhalatee san ulfaatni isaa safaramee turee?	1 = Eeyyeen<br>2 = Lakki – Gara 804 deemi<br>3 = Hin beeku– Gara 804 deemi	|__|
803	Eeyyeen yoo tahe, ulfatinni [Maqaa daa’imaa] yeroo dhalatu meeqa ture?	Ulfina graamaan guuti FKN. Ulfinni kg 1.9 yoo tahe 1900 barreessi; Hin beeku 9999. Yoo danda’ame kaardii irraa kan galmaa’e barreessi	|__|__|__|__|
804	[MAQAA DAA’IMAA] yeroo dhalatu Booyuu/ hafuura fudhachuu dadhabee turee?	1 = Eeyyeen<br>2 = Lakki – Gara 807 deemi	|__|

[MAQAA DAA’IMAA] yeroo dhalatu namnii kanneen keessaa tokko godheef jiraa?<br><br>Kan deebi’an qofa filadhu	Tokkon tokkoon gaafiileetiif: 1 = Eeyyeen 2 = Lakki		
	805	sukkumuu/harkaan qaqqabachuu	|__|
	806	Hafuura afaaniin keennuufii	|__|

807	[MAQAA DAA’IMAA] yeroo dhalateen booda eessa kaa’ame?	1 = Lafa irra<br>2 = Garaa/Qoma haadhaa irra<br>3 = Haadhaa cinaa/bukkee<br>4 = Nama biraa bira<br>5 = Kan biro<br>6 = Hin beeku	|__|
808	[MAQAA DAA’IMAA] dhalatee daqiiqaa meeqa booda qaamni/dhaqni isaa/ishee goggoofame/qoorfame?<br><br>*Hubadhu: Kan lakkaawamu yeroo daa’imni dhalatee booda malee, erga obbaatiin bahe boodaa miti*	Daqiiiqaawwan barreessii, Hin beeku yoo tahe. 999 barreessii	|__||__||__|
809	[MAQAA DAA’IMAA] yeroo dhalatee daqiiqaa meeqaa booda huccuun/huffataan haguugame?<br>*Hubadhu: Kan lakkaawamu yeroo daa’imni dhalatee booda*	Daqiiiqaawwan barreessii,Hin beeku yoo tahe. 999 barreessii	|__||__||__|

	malee, erga obbaatiin bahe boodaa miti		
810	Haandhuura gudunfuuf maal fayyadamtan?	1 = Haada affeelame/jirbii haaraa<br>2 = Haada/jibrii kanum argame<br>3 = Haada argame<br>4 = Klaampii<br>5 = Homaanu hin guduunfine<br>6 = Hin beeku<br>7 = Kan biroo	|__|
811	Handhuuraa murudhaaf maal fayyaadamtan?	1 = Haadduu haaraan<br>2 = Haadduu kan argamteen<br>3 = Maqasii affeelameen<br>4 = Maaqaasii kan argameen<br>5 = Hin beeku<br>6 = kan biroo	|__|
812	Haandhuurri murame erga guduunfame booda waanti dibame jiraa?	1 = Eeyyeen<br>2 = Lakki – Gare 824 deemi	|__|

Eeyyeen yoo tahe, handhuurri yeroo muramu maaltu dibame?<br><br>**Filannoowaan hin dubbisiin,kan deebii'an hundaa deebisii**		Tokkon tokkoon gaafiileetiif: 1 = Eeyyeen 2 = Lakki	
	813	Dhadhaa	|__|
	814	Daaraa	|__|
	815	Dibata (kan dawaa/qoricha hin taanee)	|__|
	816	Faaltii/dhoqqee beeladootaa/horii	|__|
	817	Zeeyita	|__|
	818	Bishaan qabanaawaa	|__|
	819	Kan biroo	|__|

820	Dawaan/qorichi jarmii ittisu handhuura irra godhameefi ture?	1 = Eeyyeen<br>2 = Lakki –Gara 824 deemi<br>3 = Hin beeku – Gara 824	|__|
821	Eeyyeen yoo tahe, dawaan/qorichi sun 'chlorhexidine' jadhamu ture?<br><br>(Gaafataa: yaabootii iddeessaa/naamunaa ykn fakkii chlorohexidine muldhiisiif)	1 = Eeyyeen<br>2 = Lakki – Gara 824<br>3 = Hin beeku – Gara 824	|__|
822	Eeyyeen yoo tahe, dawaan 'chlorhexidine' jadhamu guyyoota meeqaaf dibame?	Lakkoofsa guyyaa barreessii<br>**Yoo hin beeknee 99 barreessi**	|__||__|
823	Eeyyeen yoo tahe, guyyaatti yeroo meeqaa kilorookseyidiin 'chlorhexidine' dibameef	guyyaa tokkotti yeroo meeqa akka godhamee lakkoofsa barreessii<br>**Yoo hin beeknee 99 barreessi**	|__||__|

824	[MAQAA DAA'IMAA] akkuma dhalateen dawaan/qorichi ijaa TTC godhameefi ture?	1 = Eeyyeen<br>2 = Lakki<br>3 = Hin beeku	|__|
825	[MAQAA DAA'IMAA] yeroo dhalate, yeroo meeqa booda qaamni isaa/ishee dhiqame?	1 = Sa'aatii tokko keessatti<br>2 = Sa'aatii tokko booda – Gara 827<br>3 = Guyyaa tokko booda – Gara 828	|__|
826	Sa'aatii tokko keessa yoo tahe, daqiiqaa meeqa booda?	Lakkoofsa daqiqaa barreessi<br>Hin beeku yoo tahe, 99 barreessii	|__||__|
827	Sa'aatii tokko booda yoo tahe, sa'aatii meeqaa booda?	Lakkoofsa sa'aaatii guutuu barreessi<br>Fkn. Yoo deebiin sa'aati tokko ta'e 1,yoo deebiin sa'aatii tokkof walakkaa ta'e 1,yoo deebiin sa'aatii lama ta'e 2,yoo deebiin sa'aati lamaaf walakka ta'e 2 barreessii.<br><br>Hin beeku yoo tahe, 99 barreessii	|__||__|
828	Guyyaa tokko booda yoo tahe, guyyaa meeqaa booda?	Lakkoofsa guyyaa guutuu barreessi<br>Fkn.yoo deebiin guyyaa tokko booda ta'e 1,yoo deebiin guyyaa tokkoof walakka ta'e 1 barreessii.<br>Yoo deebiin guyyaa lamaaf walakka ta'e 2 barreessii<br>Hin beeku yoo tahe, 99 barreessii	|__||__|
829	Torbaan duraa keessatti, [MAQAA DAA'IMAA] akka qaamni/harmi kee fi qaamni isaawaltuquutti ofitti qabuudhaan ammatteettaa ?	1 = Eeyyeen, guyyatti yeroo 1-7<br>2 = Eeyyeen, guyyatti yeroo 8-12<br>3 = Eeyyeen, guyyatti yeroo 12 ol<br>4 = Lakki<br>5 = Hin beeku	|__|
830	Torbaan duraa keessatti, [MAQAA DAA'IMAA] halkan wajiin rafteeettaa, moo iddoo/siree biroo irratti kophaa raffifte?	1 = Haadhaa wajiin rafe<br>2 = Kophaa rafe<br>3 = Nama biroo wajiin rafe	|__|
831	[MAQAA DAA'IMAA] harma hoosiftee beekta?	1 = Eeyyeen<br>2 = Lakki	|__|
832	[MAQAA DAA'IMAA] idda dhalatee guyyoota 28 keessatti haarma qofa hoosiftee?	1 = Eeyyeen – Gara 837<br>2 = Lakki	|__|
833	Lakki yoo tahe, [MAQAA DAA'IMAA], aannan arma kee malee/alatti waan biraa maal kenniteef?	1 = Bishaan<br>2 = Aaannan beeladaa/loonii<br>3 = Dhadhaa<br>4 = Sukkaraa fi Bishaan<br>5 = Cuunfaa mudraa<br>6 = Shaayii<br>7 = Kan biroo	|__|
834	KAN BIRO yoo jiraate, Haa ibsamu.	Haa ibsamu. ______________________	

835	Aannan harma keetii alatti, waan dhugamu kan biraa maaliif keenniteef?	1 = Aannan harma kootii ga'aa miti<br>2 = Daa'immicha wajjiin guyyaa guutuu waliin waan hin olleef<br>3 = Hiriyoonni ykn Firoonni koo waan na gorsaniif<br>4 = Aadaa kootti ykn bakka jireenya kootti waan baratame waan taheef<br>5 = Kan biroo	|__|
836	KAN BIRO yoo tahe, Haa ibsamu.	Haa ibsamu. ______________________	
837	[MAQAA DAA'IMAA] erga dhalate yeroo meeqaa booda harma keetti qabde ykn arma keenniteef ?	1 = sa'aatii duraa keessatti<br>2 = Sa'aatii duraa booda (guyyuma dhalate keessatti)<br>3 = Guyyaa tokko booda	|__|
838	Aannan duraanii cuunfitee ykn elmiteet gattee?	1 = Eeyyeen<br>2 = Lakki	|__|

839	Erga [MAQAA DAA'IMAA] dhalatee torban 6 keessatti, ogeessi fayyaa waa'ee fayyaa isaa qoratee ykn ilaalee ture?<br><br>Qoronnoon yeroo dhalatee sanitti godhamee fi san booda kan godhame hunda gaafadhu	1 = Eeyyeen<br>2 = Lakki - Gara 892 deemii	|__|
840	Eeyyeen yoo tahe, [MAQAA DAA'IMAA] erga dhalate guyyaa meeqa booda qorannoon godhameef?	Lakkoofsa guyyootaa barreessii Yoo hin beeknee ta'e 99 barreessii.	|__|__|
841	Eeyyeen yoo tahe, [MAQAA DAA'IMAA] eessatti qoratame/mtee?	1 = Mana keenya keessa<br>2 = Keellaa Fayyaa<br>3 = Buufata Fayyaa<br>4 = Hospitaala<br>5 = Kan biroo	|__|
842	Eeyyeen yoo tahe, Fayyaa [MAQAA DAA'IMAA] Eenyutu ilaale?	1 = Dooktoota<br>2 = Narsii/Deessiftu<br>3 = HEF<br>4 = Qondaala Fayyaa<br>5 = RMF	|__|
843	[MAQAA DAA'IMAA] dhalatee torban 6 keessatti, yeroo lammaffaaf ilaalameeraa/qoratameraa?	1 = Eeyyeen<br>2 = Lakki - Gara 855	|__|
844	Eeyyeen yoo tahe, [MAQAA DAA'IMAA] yeroo lammaffaf oggu ilaalamu idda dhalate guyyoota meeqa ture?	Lakkoofsa guyyaa barreessii<br>Yoo hin beeknee 99 barreesii	|__|__|
845	Eeyyeen yoo tahe, [MAQAA DAA'IMAA] eessatti ilaalame?	1 = Mana keenya keessa<br>2 = Keellaa Fayyaa<br>3 = Buufata Fayyaa<br>4 = Hospitaala<br>5 = Kan biro	|__|
846	Eeyyeen yoo tahe, Fayyaa [MAQAA DAA'IMAA] Eenyuutu ilaale?	1 = Dooktoota<br>2 = Nursii/ Deessiftu<br>3 = HEF<br>4 = Qondaala Fayyaa<br>5 = RMF	|__|

847	[MAQAA DAA'IMAA] dhalatee torban 6 keessatti, yeroo sadaffaaf ilaalameera/qoratameraa?	1 = Eeyyeen<br>2 = Lakki - Gara 855	|__|
848	Eeyyeen yoo tahe, [MAQAA DAA'IMAA] yeroo sadaffaaf oggu ilaalamu idda dhalate guyyoota meeqa ture?	Lakkoofsa guyyaa barreessii<br>Yoo hin beekne 99 barreessii	|__|__|
849	Eeyyeen yoo tahe,[MAQAA DAA'IMAA] eessatti ilaalame?	1 = Mana keenya keessa<br>2 = Kellaa fayyaa<br>3 = Buufata Fayyaa<br>4 = Hospitaala<br>5 = Kan biro	|__|
850	EEYYEEN yoo tahe , [MAQAA DAA'IMAA] Eenyuuttu ilaale/qorate?	1 = Dooktoota<br>2 = Narsii/Deessiftu<br>3 = HEF<br>4 = Qondaala Fayyaa<br>5 = RMF	|__|
851	Qorannoon Arfaffaan gaggeefame ture?	1 = Eeyyeen<br>2 = Lakki - Gara 855	|__|
852	Eeyyeen yoo tahe, [MAQAA DAA'IMAA] yeroo ilaalamu idda dhalate guyyoota meeqa ture?	Lakkoofsa guyyaa barreessii<br>Yoo hin beekne 99 barreessii	|__|__|
853	Eeyyeen yoo tahe,[MAQAA DAA'IMAA] eessatti ilaalame?	1 = Mana keenya keessa<br>2 = Keellaa fayyaa<br>3 = Buufata fayyaa<br>4 = Hospitaala<br>5 = Kan biro	|__|
854	EEYYEEN yoo tahe, [MAQAA DAA'IMAA] Eenyuuttu yeroo arfaffaaf ilaale/qorate?	1 = Dooktoora<br>2 = Narsii/Deessiftu<br>3 = HEF<br>4 = Qondaala Fayyaa<br>5 = RMF	|__|

Yeroo qorannoowan fayyaa [MAQAA DAA'IMAA] gaggeeffaman sana maal maaltu godhameefi ture? Kan ilaallatu hunda barreessi.

Gaafataa:.Fillannoowan kanaan gadi jiran sagalee ol kaasii dubbiisiif.
Qorannoon fayyaa [MAQAA DAA'IMAA] yoo yeroo murtaa'eetti hin adeemsiifameennee, tarree saan bakka duwaa dhiisii

	Tokkon tokkoo gaafiileetiif: 1 = Eeyyeen 2 = Lakki					
	(Gaafii #)	Guyyoota 0-2	(Gaafii #)	Guyyoota 3-7	(Gaafii #)	Guyyoota 8-42
Qaama daa'imaa waligalatti ilaaluun	855	|__|	856	|__|	857	|__|
Ulfatinnaa (kiiloo) isaa maadaluun	858	|__|	859	|__|	860	|__|
Handhuura ilaaluun	861	|__|	862	|__|	863	|__|
Waa'ee harma hoosiisuu gorsuu	864	|__|	865	|__|	866	|__|
Akkaata haati harma hoosiftu ilaaluu	867	|__|	868	|__|	869	|__|
Qaamni daa'imichaa fi kan haadhaa akka wal tuqu gorsuu	870	|__|	871	|__|	872	|__|

Mallattoowwan balaa sakata'uu ykn qorachuu (mallattoowan dhibee hamaa dabalatee)	873	|__|	874	|__|	875	|__|
Waa'ee mallattoowwan balaa barnoota kennuu	876	|__|	877	|__|	878	|__|
Gara dhaabbata fayyaatti erguu (riifarii gochuu)	879	|__|	880	|__|	881	|__|
Daa'ima tuqu duratti haarka dhiqachuun akka barbaachisu barumsa kennuu	882	|__|	883	|__|	884	|__|
Qulqullinni handuuriadaa'imaa akka eegamu gorsuu	885	|__|	886	|__|	887	|__|
Daa'imaa dhalatee sa'aatii 24 keessatti qaamni akka hin dhiqamne gorsu	888	|__|				

889	Kunuunsa (tajaajila) daa'ima kee kan haaraa dhalateef keennametti akka itti quufte fi itti hin quufune nuutti himuu dandeessaa?<br><br>Filannowwan hin dubbisin	1 = Eeyyeen itti quufeera<br><br>2 = Lakki itti hin quufne (Gara 891)<br>3 = Itti quufeeras itti hin quufnes jechuu hin danda'u (Gara 892)	|___|
890	EEYYEEN yoo tahe, ammam itti quufte?<br><br>Filannowwan Lachuu Dubbisi	1 = Guutuu guutuutti itti quufeera(Gara 892)<br>2 = Hanga tokko itti quufeera (Gara 892)	|___|
891	LAKKI yoo tahe, ammam itti hin quufne?<br>Read both options	1 = Guutumatti hin quufnee<br>2 = hammaa tokko itti hin quufnee	|___|
892	Daa'ima kee kan diwoo deesse, gara alaatti osoo hin baasiin guyyoota/torban muraasaaf mana keessatti teessifteettaa?	1 = Eeyyeen<br>2 = Lakki – Gara 894	|___|
893	EEYYEEN YOO TAHE, Guyyoota meeqaaf?	Lakkoofsa guyyootaa barreesii<br>Yoo hin beekamnee 99 barreessii	|___||___|
894	Daa'ima kee isa diwoo deesse, erga dhalate guyyoota meeqaa boodatti namoonni isa ilaaluuf dhufan? Fkn: firoota kee, HEF, hiriyoota.	Lakkoofsa guyyootaa barreessii<br>Yoo hin beekamnee 99 barreessii	|___||___|
895	Daa'ima kee isa diwoo deesse, daa'imich erga dhalate guyyoota meeqaa boodatti (si male) namoonni biraa harkaan tuqan?	Lakkoofsa guyyootaa barreessii<br>Yoo hin beekamnee 99 barreessii	|___||___|

9. Waa'ee Kunuunsaaa Daa'imman Haaraa dhalatanii Dhukkubsatanii

900	**Gaafataa – hubadhu: Daa'imni Fulbaana bara 2004 booda dhalate? Akkaa lakkoofsa Itiyoopiyaatti**<br><br>**Eeyyeen yoo tahe, Gaafii kee itti fufi.**<br>**Lakki yoo thae, Gaafii asitti dhaabiti dubartii umriin 13-49 kan biroo maatii kana keessatti argamtutti itti fufi.**	1 = Eeyyeen<br>2 = Lakki-**Gaafii asitti dhaabi**	|__|
**Gaafataa*: Amma, [Maqaa daa'imaa] dhalate guyyoota 28 keessatti dhukkubni isa mudate (yoo jiraate) waa'ee issaa si***			

gaafachuun barbaada.			
901	[MAQAA DAA’IMAA] dhalatee guyyoota 28 keessatti dhukkubsatee beekaa?	1 = Eeyyeen<br>2 = Lakki	|__|

[MAQAA DAA’IMAA] dhalatee guyyoota 28 keessatti, mallattoowwan armaan gadii irratti mul’atanii ni beeku?
Tarreewwan keessaa mallattowwan sagalee ol kaasii dubbisi.

Eeyyeen yoo tahe 1 barreessi; Roga (row) kana hunda itti deemi<br><br>Lakki yoo tahe 2 barreessii Gara roga(row) itti aanutti darbi<br><br>Mallattowwan kessaa tokkoyyuu yoo hin jiraanne , Gara Kutaa 10 darbi			Umrii (yeroo mallattoon duraa mul’ate)<br><br>Umrii lakkkoofsa guyyaatiin galmeessi		Wal’aansa barbaaddeefii ykn mana yaalaa geessitee ture?<br><br>1 = Eeyyeen<br>2 = Lakki		Eeyyeen yoo tahe, eenyu birraa?<br><br>1 = Keellaa fayyaa<br>2 = Buufata fayyaa<br>3 = Hospitaala<br>4 = RMF<br>5 = Walaansa Aadaa<br>6 =Mana /dukkaana orichaa		Yoo wal’aansiabarbaad eefii ykn mana yaalaa geessitee ture, Eenyyuutu (maaqa daa’imaa) wal’aane?<br><br>1 = HEF<br>2 = Nursi<br>3 = Qond. Fayyaa<br>4 = farmaasisti<br>5 = Dooktoora<br>6 = Ogeessa Aadaa<br>6 = Kan biro		Yoo gara wal’aansaa ykn mana yaalaa hin geessine, maaliif?<br><br>1 = Ni wayaawwa jannee waan yaanneef<br>2 = Dhabbannii fayyaa waan fagaatuuf<br>3 = Gatiin bayyee ykn mi’aa waan ta’eef<br>4 = Dhaabbata fayyaa waan hin amanneef<br>5 = Miseensii maatii waan na dhorkeef<br>6 = Gorsaan hawaasaa ykn jaarsi biyyaa akkaan hin goone waan na gorseef<br>7 = Kan biroo	
Harma hodhuu dadhabuu ykn xiqqoo hodhuu	902	|__|	903	|__||__|	904	|__|	905	|__|	906	|__|	907	|__|
Ulfatina barbaachisuu gad tahu	908	|__|	909	|__||__	910	|__|	911	|__|	912	|__|	913	|__|
Hafuurri ciccituu ykn daddafee hafuura baasuu	914	|__|	915	|__||__|	916	|__|	917	|__|	918	|__|	919	|__|
Qomni/laphen bu’uu ykn keessaa senu	920	|__|	921	|__||__|	922	|__|	923	|__|	924	|__|	925	|__|
Baayyee oo’uu ykn qorruu	926	|__|	927	|__||__|	928	|__|	929	|__|	930	|__|	931	|__|
Dhaqni isaa laafuu	932	|__|	933	|__||__|	934	|__|	935	|__|	936	|__|	937	|__|
Harki/miilli /ijji keelloo ta’uu	938	|__|	939	|__||__|	940	|__|	941	|__|	942	|__|	943	|__|
Garaa kaasuu ykn albaasuu	944	|__|	945	|__||__|	946	|__|	947	|__|	948	|__|	949	|__|
Hollachuu	950	|__|	951	|__||__|	952	|__|	953	|__|	954	|__|	955	|__|

Qaamatti waa itti ya'uu ykn madaa'u	956	|__|	957	|__||__|	958	|__|	959	|__|	960	|__|	961	|__|
Handhuurri malaa'u ykn jiidhuu	962	|__|	963	|__||__|	964	|__|	965	|__|	966	|__|	967	|__|
Kan biroo (Haa ibsamu.)	968	|__|	969	|__||__|	970	|__|	971	|__|	972	|__|	973	|__|
Haa ibsamu. __________												

Gaafataa:Gaafiiwwaan 974-983 jiran kan gaafataman yoo mallatto/dhibeewan armaan oliif waldhaansi barbaadame ykn mana yaalaa dhaqame ture duwwaa dha.

974	Mallattowwan armaan olii tokkolleen yoo mul'atan guyyaa meeqa booda gara wal'aansaatti yeroo jalqabaaf geessite?	**Guyyaa dhukkubbiin jalqabee irraa kaasee lakk. guyyoota galmeesi; Guyyaa mallattoon mul'ate yoo tahe 0 galmeessi, Yoo jiraate kardi ilaaluun mirkaneessi**<br><br>**Yoo wal'aansaaf hin geeffamne 99 barreessii**	|__|__|
975	[MAQAA DAA'IMAA] dhukkubni cimaan oggeessa fayyaa tiin irratti argamee ture?	**1 = Eeyyeen**<br>**2 = Lakki**	|__|
976	[MAQAA DAA'IMAA] dhukkuba issaaf/isheef dawaan ajajameefii ture?	**1 = Eeyyeen**<br>**2 = Lakki**	|__|
977	[MAQAA DAA'IMAA] guyyoota 7 walitaansee qoricha jeentaamaaysin jedhamu waraaname ture?<br>**Gaafataa: kola ykn fakkii jeentaamaaysin agarsiisi**	**1 = Eeyyeen**<br>**2 = Lakki**	|__|
978	[MAQAA DAA'IMAA] guyyoota 7 walitaansee qoricha Amoxaasiliin jedhamu fudhate ture?<br>**Gaafataa : Amooxaaciliin kiniinii bishaaniin xammisiisuun hunsuusan akka tahe ibsiif**	**1 = Eeyyeen**<br>**2 = Lakki**	|__|
979	[MAQAA DAA'IMAA] yeroo dhukkubsate/tte, qorichoota (dawaalee) fudhatee ture?	**1 = Eeyyeen**<br>**2 = Lakki**	|__|
980	[MAQAA DAA'IMAA] aannan harmaa kan elmameen waldhaanamee ture?	**1 = Eeyyeen**<br>**2 = Lakki**	|__|
981	Wal'aansa ykn yaalii daa'ima kee haaraa dhalatee dukkuubsateef godhameetti akka itti quufte fi itti hin quufune natti himu dandeessaa?<br>Filannowwan hin dubbisin	**1 = Eeyyeen Itti quufeera**<br>**2 = Lakki itti hin quufne (Gara 983)**<br>**3 = itti quufeeras itti hin quufnes jachu hin danda'u (Gara kutaa 10)**	|___|
982	EEYYEEN YOO TAHE, ammam itti quufte?<br><br>Filannoowwan lachuu dubbisi	**1 = guutuu guututti itti quufeera (Gara kutaa 10)**<br>**2 = Hanga tokko itti quufeera (Gara kutaa 10)**	|___|
983	LAKKI yoo tahe, ammam itti hin quufne?<br><br>Filannoowwan lachuu dubbisi	**1 = guutuu guutuutti itti hinquufne**<br>**2 = Hanga tokko itti hin quufne**	|___|

Kutaa 10. Waa’ee Daa’imman Lubbuun Hin Jiraannee (kan guyyoota 28 osoo hin geenyiin du’an)

Gaafataa: Yoo daa’immni sun dhalatee guyyaa 28 osoo hin gayiin du’e, gaaffiwan armaan gadii (Waa’ee mallattowwanii, wal’aansa keennameef, fi sababa du’a isaa) gaafadhu.

Waa’ee daa’immaan lubbuun isaani darbee hasawuun baay’ee akka ulfaatu nan beeka. Kanaaf yoo takka turuu/boqochuu barbaaddan sinin eega. Deebiiwan isin nuuf kennitan ykn odeefannoon muuxanno keessanirra argannu mootummaan akka faayyaa daa’immaani fooyyessuuf sagantaa baafatuu kaaraa saaqa.

Du’a (MAQAA DAA’IMAA) dura mallattoowwan isaan kamii irratti agarte ture?<br><br>Deebiwwan keennaman hunda filadhu	Tokkon tokkon gaafiileetiif: 1 = Eeyyeen 2 = Lakki		
	1000	Hafuurri ciccituu/ hafuura baasuu dadhabuu	|___|
	1001	Qomni gama keessaa seenuu (laphee bu’uu)	|___|
	1002	Qaamni baayyee o’uu ykn qorruu	|___|
	1003	Dhaqni isaa laafuu	|___|
	1004	Harki/miilli/ijji keelloo ta’uu	|___|
	1005	Garaa kaasuu ykn albaasuu	|___|
	1006	Hollachu	|___|
	1007	Harki/miilli/ijji keelloo ta’uu (repeated)	|___|
	1008	Qaamatti waa itti ya’uu ykn madaa’u	|___|
	1009	Handhuurri diimachuu, malaa’u ykn jiidhuu	|___|
	1010	Kan biroo (Haa ibsamu.)	|___|

1011	KAN BIROO yoo tahe, Haa ibsamu.	**Haa ibsamu. ________________________**	
1012	MAQAA DAA’IMAA dhukkubsatee yoo du’e ta’ee, dhukkuba saniif wal’aaname ykn gargaaramee ture?	**1 = Eeyyeen**<br>**2 = Lakki – Gara dhuma Gaafii**	
1013	Eeyyeen yoo tahe, eenyutu wal’aansa keenneefii ture?	**1 = RMF**<br>**2 = HEF**<br>**3 = Nursii**<br>**4 = Dooktoora**<br>**5 = Qondaala Fayyaa**<br>**5 = Faarmaasistii/Ogeessaa dawaa**<br>**6 = Kan biro**	|__|
1014	[MAQAA DAA’IMAA] du’a isaa dura kan wal’aaname yoo ta’ee, oggeessi fayyaa isa ilaale Sababnii du’a isaa maali jedhe?	**1 = Dhukkuba qaama haafuraa/ Nimooniyaa**<br>**2 = Garaa kaasaa (albaatii)**<br>**3 = Infeekishinii cimaa/dhibee baay’ee cimaa ta’ee**<br>**4 = Kan biro (Haa ibsamu.)___**	

1015	KAN BIRO yoo tahe Haa ibsamu	**(Haa ibsamu.)______________________**

Gaafataa: Qulqulleessi adda baasi:
1. Mana (Maatii) kana keessatti dubartiin biroo umriin 13-49 taate fi hin gaafatamin jirti?
2. Mana (Maatii) kana keessatti dubartiin Fulbaana 1, 2004 as ulfoofte ykn garaatti baatte kan biroo fi hin gaafatamin jirti?
3. Mana (Maatii) kana keessatti dubartiin guyyaa Fulbaana 1, 2004 as deesse fi hin gaafatamin jirti?
4. Mana (Maatii) kana keessatti dubartiin daa'ima haaraa dhalate qabdu fi hin gaafatamin jirti?

Gaafiwwan armaan olii kessa Tokkolleen Eeyyeen yoo tahe, isaan gaafachuu kee mirkaneessi.
Deebiin gaaffiwwan hundaafuu Lakki yoo ta'e, Dhuma Gaafii – yeroo isaanii waan siif keennaniif Hirmaattoota Galatoomfadhu.
