# Supplementary material for: Coverage of antenatal, intrapartum, and newborn care in 104 districts of Ethiopia: A before and after study four years after the launch of the national Community-Based Newborn Care programme
Source: PLoS One. 2021 Aug 5;16(8):e0251706. doi: 10.1371/journal.pone.0251706 (PMC8341496; doi:10.1371/journal.pone.0251706)
Supplement: S4 File — (PDF) [file pone.0251706.s004.pdf]

ማህበረ ሰብ አቀፍ የጨቅላ ሕጻናት ክብክቤ በኢትዮጵያ
የቤት ለቤት ዳሰሳዊ መጠይቅ

COMMUNITY BASED NEWBORN CARE IN ETHIOPIA
HOUSEHOLD SURVEY QUESTIONNAIRE V 2.1

ሞድዩል 1

<table>
<tr><td colspan="4">ክፍል1 ፡ የቤተሰብ መለያ መግለጫ</td></tr>
<tr><td>100</td><td>ቀን/ወር/ዓ/ም</td><td colspan="2">|__|__| / |__|__| / |__|__|__|__|</td></tr>
<tr><td>101</td><td>ክልል</td><td colspan="2">|__|__|__|__|__|__|__|__|__|__|__|__|__|__|__||__|__|__|__|__|__|__|</td></tr>
<tr><td>102</td><td>ዞን</td><td colspan="2">|__|__|__|__|__|__|__|__|__|__|__|__|__|__|__||__|__|__|__|__|__|__|</td></tr>
<tr><td>103</td><td>የወረዳዉ ስም</td><td colspan="2">|__|__|__|__|__|__|__|__|__|__|__|__|__|__|__||__|__|__|__|__|__|__|</td></tr>
<tr><td>104</td><td>የመሠረታዊ የጤና እንክብካቤ ዓሀድ ጥምርት/ጉድኝት (Cluster) /ጤና ጣቢያ ስም</td><td colspan="2">|__|__|__|__|__|__|__|__|__|__|__|__|__|__|__||__|__|__|__|__|__|__|</td></tr>
<tr><td>105</td><td>የቀበሌዉ ስም</td><td colspan="2">|__|__|__|__|__|__|__|__|__|__|__|__|__|__|__||__|__|__|__|__|__|__|</td></tr>
<tr><td>106</td><td>የጎጥ ስም</td><td colspan="2">|__|__|__|__|__|__|__|__|__|__|__|__|__|__|__||__|__|__|__|__|__|__|</td></tr>
<tr><td>107</td><td>ጥምርት/ጉድኝት (Cluster) ቁጥር ይመዝገብ</td><td colspan="2">|__|__|__|</td></tr>
<tr><td>108</td><td>የቤት ተራ ቁጥር
የቤተሰብ ቁጥር ይመዝገብ</td><td colspan="2">|__|__|</td></tr>
<tr><td>109</td><td>የተለየ የቤት ተራ መለያ ቁጥር
በሁሉም ሰነዶች ላይ ይስፈር
ለምሳሌ፡ የተሳታፊ መረጃ ቅጽ</td><td colspan="2">|__|__|__| / |__|__|

ጥምርት/ጉድኝት / ቤት ተራ ቁጥር</td></tr>
<tr><td>110</td><td>ጂፕኤስ የምድር ሰቅ (የቤቱ)</td><td colspan="2">|__|__| : |__|__|__|__|__|</td></tr>
<tr><td>111</td><td>ጂፕኤስ የምድር አካፋይ ዋልታ (የቤቱ)</td><td colspan="2">|__|__| : |__|__|__|__|__|</td></tr>
<tr><td>112</td><td>የአባወራ/እማወራ ሥም</td><td colspan="2">|__|__|__|__|__|__|__|__|__|__|__|__|__|__|__|__|__|</td></tr>
<tr><td>113</td><td>የመጀመረያ ጉብኝት ቀን</td><td colspan="2">ቀን |__|__| ወር |__|__| ዓመተ ምህረት |__|__|__|__|</td></tr>
<tr><td>114</td><td>የጠያቂ ው/ዋ ሥም</td><td colspan="2">______________________________</td></tr>
<tr><td>115</td><td>ጠያቂ፡ የተሳትፎ ስምምነት መረጃ ቅጽ ተነቦላታል/ቦለታል?</td><td>1 = አዎ
2 = ኤይ</td><td>|__|</td></tr>
</table>

116	**ጠያቂ፡ ተጠያቂው ተስማምተዋል?**	1 = አዎ<br>2 = ኤይ *መጠይቁን በማቋረጥ ወደ ሚቀጥለው ቤት ይሂዱ::*	|__|
	ይገለጹ	________________________________	
117	የቤተሰብ ሀላፊው/ ሀላፊዋ ብሄር ምንድነው?	1 = አገው<br>2 = አማራ<br>3 = ቤንች<br>4 = ቡርጂ<br>5 = ዲዚ<br>6 = ጌድኦ<br>7 = ጉራጌ<br>8 = ሃድያ<br>9 = ከፊቾ<br>10= ከምባታ<br>11 = ኮንታ<br>12 = ሜኒት<br>13 = ኦሮሞ<br>14 = ሲልጤ<br>15 = ትግራይ<br>16= ዎላይታ<br>17 = የተቀሩት ኢትዮጵያዊ የብሄረሰብ አካላት<br>18 = ከሁለት ወይንም በላይ ብሄር	|__|__|

ጉብኝት	1ኛ	2ኛ	3ኛ	የመጨረሻ ጉብኝት
ቀን (ቀን/ወር/ዓመት)	|__|__| / |__|__| / |__|__|__|__|	|__|__| / |__|__| / |__|__|__|__|	|__|__| / |__|__| / |__|__|__|__|	ቀን |__|__| ወር |__|__| ዓመት

<table>
<tr><td colspan="2">የጠያቂ ው/ዋ ሥም</td><td></td><td></td><td></td><td rowspan="4">|__|__|__|__|
ውጤት |__|</td></tr>
<tr><td colspan="2">ውጤት(አስፈላጊውን መለያ ከዚህ በታች ያስገቡ)</td><td>|__|</td><td>|__|</td><td>|__|</td></tr>
<tr><td rowspan="2">መጠይቅ በዕለቱ ካልተጠናቀቀ ቀጣዩ ጊዜ መቼ ይሆናል (ቀጠሮ ይያዝ)</td><td>ቀን (ቀን/ወር/ዓመት)</td><td></td><td>|__|__| /
|__|__| /
|__|__|__|__|</td><td>|__|__| /
|__|__| /
|__|__|__|__|</td></tr>
<tr><td>ሰዓት</td><td></td><td></td><td></td></tr>
<tr><td colspan="6">የውጤት ኮድ፡
1. የተሟላ
2. በግማሽ የተሟላ
3. የተላለፈ
4. ቤት ውስጥ ያልተገኙ
5. መጠይቁን ለመመለስ የማይችሉ
6. ፈቃደኛ አየደሉም</td></tr>
</table>

ፍቃደኛ ካልሆኑ ይቆማል።

<table>
<tr><td rowspan="2">118</td><td colspan="8">ቤተሰብ ዝርዝር
እዚህ ቤት ስለሚኖሩ ነዋሪዎች መጠየቅ እፈልጋለው:: ነዋሪች ስል ባለፉት 3 ወራት እዚህ የነበሩና ምግብ አብራቹ የሚመገቡ ማለት ነው::
እባክዎ እርስዎንም ጨምሮ የሌሎችንም የዚህ ቤት ነዋሪዎችን ሥም መጠየቅ እችላለሁ (ከቤተሰብ 🕒ላፊው ጀምረው እንደ ዕድሜ ቅደም ተከተላቸው ከትልቅ ወደ ትንሹ ዕድሜ ይዘርዝሩልኝ::)</td></tr>
<tr><td>የግለሰቡ/ባ ሥም</td><td>ፆታ</td><td>የትውልድ ቀን
ቀን/ወር/ዓመት</td><td>ዕድሜ</td><td>ከ13-49 ዓመት እድሜ ውስጥ የሚካተቱ ሴቶች</td><td>የጋብቻ ሁኔታ</td><td>ያጠናቀቁት መደበኛ የትምሕርት ደረጃ በቁጥር ይግባ</td><td>ሃይማኖት</td></tr>
<tr><td>ተራ ቁጥር</td><td>የመጀመሪያ ሥም ብቻ ይግባ
(ተመሳሳይ ስም ካለ በቤት ውስጥ የአባትየውን የመጀመሬያ የስም ፊደል ይጠቀሙ)</td><td>1 = ወንድ
2 = ሴት</td><td>(ቀን/ወር/ዓመት)
ቀን የማይታወቅ ከሆነ 99 ይፃፍ
ወር የማይታወቅ ከሆነ 99 ይፃፍ
ዓመት የማይታወቅ ከሆነ 9999 ይፃፍ</td><td>ዕድሜያቸው በዓመት ይፃፍ

እድሜ ከአንድ አመት ያነሰ ከሆነ 0 ይፃፍ</td><td>1 = አዎ
2 = አይ</td><td>1 =ያገቡ
2 =አብረው የሚኖሩ
3 = ያላገቡ
4 = የተፋቱ
5 = በሞት የተለዩ
6 = አይመለከትም</td><td>መደበኛ ት/ት የተጠናቀቀበትን ዓመታት ብዛት ይገለጽ</td><td>1 = ኦርቶዶክስ
2 = ካቶሊክ
3 = ፕሮቴስታንት
4 = እስላም
5 = ሌሎች
6 = አይመለከትም</td></tr>
<tr><td>1</td><td>|_|_|_|_|_|_|_|_|_|_|_|_|_|_|_|_|_|_|_|_|</td><td>|__|</td><td>|__|__|/|__|__|/|__|__|__|__|</td><td>|__|__|</td><td>|__|</td><td>|__|</td><td>|__|__|</td><td>|__|</td></tr>
<tr><td>2</td><td>|_|_|_|_|_|_|_|_|_|_|_|_|_|_|_|_|_|_|_|_|</td><td>|__|</td><td>|__|__|/|__|__|/|__|__|__|__|</td><td>|__|__|</td><td>|__|</td><td>|__|</td><td>|__|__|</td><td>|__|</td></tr>
<tr><td>3</td><td>|_|_|_|_|_|_|_|_|_|_|_|_|_|_|_|_|_|_|_|_|</td><td>|__|</td><td>|__|__|/|__|__|/|__|__|__|__|</td><td>|__|__|</td><td>|__|</td><td>|__|</td><td>|__|__|</td><td>|__|</td></tr>
<tr><td>4</td><td>|_|_|_|_|_|_|_|_|_|_|_|_|_|_|_|_|_|_|_|_|</td><td>|__|</td><td>|__|__|/|__|__|/|__|__|__|__|</td><td>|__|__|</td><td>|__|</td><td>|__|</td><td>|__|__|</td><td>|__|</td></tr>
<tr><td>5</td><td>|_|_|_|_|_|_|_|_|_|_|_|_|_|_|_|_|_|_|_|_|</td><td>|__|</td><td>|__|__|/|__|__|/|__|__|__|__|</td><td>|__|__|</td><td>|__|</td><td>|__|</td><td>|__|__|</td><td>|__|</td></tr>
<tr><td>6</td><td>|_|_|_|_|_|_|_|_|_|_|_|_|_|_|_|_|_|_|_|_|</td><td>|__|</td><td>|__|__|/|__|__|/|__|__|__|__|</td><td>|__|__|</td><td>|__|</td><td>|__|</td><td>|__|__|</td><td>|__|</td></tr>
<tr><td>7</td><td>|_|_|_|_|_|_|_|_|_|_|_|_|_|_|_|_|_|_|_|_|</td><td>|__|</td><td>|__|__|/|__|__|/|__|__|__|__|</td><td>|__|__|</td><td>|__|</td><td>|__|</td><td>|__|__|</td><td>|__|</td></tr>
<tr><td>8</td><td>|_|_|_|_|_|_|_|_|_|_|_|_|_|_|_|_|_|_|_|_|</td><td>|__|</td><td>|__|__|/|__|__|/|__|__|__|__|</td><td>|__|__|</td><td>|__|</td><td>|__|</td><td>|__|__|</td><td>|__|</td></tr>
<tr><td>9</td><td>|_|_|_|_|_|_|_|_|_|_|_|_|_|_|_|_|_|_|_|_|</td><td>|__|</td><td>|__|__|/|__|__|/|__|__|__|__|</td><td>|__|__|</td><td>|__|</td><td>|__|</td><td>|__|__|</td><td>|__|</td></tr>
<tr><td>10</td><td>|_|_|_|_|_|_|_|_|_|_|_|_|_|_|_|_|_|_|_|_|</td><td>|__|</td><td>|__|__|/|__|__|/|__|__|__|__|</td><td>|__|__|</td><td>|__|</td><td>|__|</td><td>|__|__|</td><td>|__|</td></tr>
</table>

አሁን የዚህን ቤተሰብ ያኗኗር ሁኔታ ለመጠየቅ እፈልጋለው፡፡<br>**ጠያቂ: በዚህ ቃለ መጠይቅ ውስጥ በያንዳንዱ ክፍል ያሉትን ጥያቄዎች ሲጠየቁ <u>አይነበብ</u> የሚል ትእዛዝ ከሌለ የምርጫ ዝርዝሮቹ ለተጠያቂው/ዋ ይነበቡ።**			
119	ግድግዳው በዋናነት የተሰራው ከምንድን ነው?	1 = ግድግዳ የሌለው<br>2 = ተፈጥሮዋዊ ዕቃ(አገዳ፣እንጨት፣ጭቃ)<br>3 = ድንጋይና ጭቃ<br>4 = ድንጋይ/ብሎኬት በሲሚንቶ<br>5 = ሌላ	|__|
120	ወለሉ በዋናናት የተሰራው ከምንድነው?	1 =<br>ተፈጥሮዋዊ ወለል (መሬት/አሸዋ/ እበት<br>2 = ቀላል ወለል (እንጨት/ዘንባባ/ ቀርቀሃ)<br>3 = ያለቀ ወለል (የጣውላዕንጨት/ላስቲክ ንጣፍ/ ጡብ ሸክላ /ንጣፍ ሲሚንቶ/ ምንጣፍ)<br>4 = ሌላ	|__|
121	ጣሪያው በዋናናት የተሰራው ከምንድነው?	1 = ከሳር ከዳን/ቅጠል<br>2 = ከቆርቆሮ ወይንም ጡብ<br>3 =ሌላ	|__|
122	ቤተሰቡ የሚጠቀምበት መጸዳጃ ምን ዓይነት ነው?	1 = የላቸውም/ጫካ/ሜዳ<br>2 = የጉድጓድ መቀመጫ ያለው<br>3 = ውሃ መልቀቂያ ያለው	|__|
123	ይህ ቤተሰብ በዋናናት የሚጠቀምበትን የመጠጥ ውሃ ከምን ያገኛል?<br><br>**አይነበብም**	1. ፓይፕ ቀጥታ መስመር ወደ ቤት የገባ<br>2. ፓይፕ ቀጥታ መስመር ወደ ጊቢው የገባ<br>3. የአካባቢው (የመሀበር) ፓይፕ<br>4. የጉደጓድ ውሃ<br>5. የተጠበቀ የተቆፈረ ጉድጓድ ውሃ<br>6. የተጠበቀ ምንጭ ውሃ<br>7. ከዝናብ ውሃ የተጠራቀመ<br>8. የመሬት ውሃ (የወንዝ፣የሐይቅ፣ ኩሬ)<br>9. ክፈት የሆነ ጉድጓድ ውሃ<br>10. ያልተጠበቀ ምንጭውሃ<br>11. ከሱቅ ወይንም ከአከፋፋይ የሚገዛ<br>12. እሸግ ውሃ<br>13. ታንከር	|__||__|
124	ይሄን ውሃ ለመጠጣት ንፅህ እንዲሆን የምታደርጉት ጥረት አለ?	1 = አዎ<br>2 = አይ (ወደ 127)<br>3 = አላውቅም (ወደ 127)	|__|
125	አዎ ከሆነ ዋናው የምታደርጉ ነገር ምንድን ነው?<br><br>**አይነበብም**	1 = ዝቃጩ እስኪረጋ በመጠበቅ<br>2 = በጨርቅ በማጥለል<br>3 = የውሃ ማጥለያ በመጠቀም (አፈር/አሸዋ/በስባሽ/ወዘተ)<br>4 = ማፍላት<br>5 = በፀሀይ ጨረር ማፍላት<br>6 = በዘመናዊ የውሃ ማከሚያ/ክሎሪን መሀኒት በመጨመር<br>7 = ሌላ ካለ ይገለጽ________________<br>8 = አላውቅም	|__|
126	ሌላ ካለ ይገለጽ	ይገለጽ____	
127	ምን ዓይነት ሃይል ነው ለምግብ ማብሰያነት ቤተሰቡ በዋናንት የሚጠቀመው?<br>**አይነበብም**	1 = ኩበት<br>2 = የማገዶ እንጨት/አገዳ<br>3 = ከሰል	|__|

<table>
<tr><td></td><td></td><td>4 = ቡታ ጋዝ
5 = ላምባ
6 = ኮሬንቲ
7 = ሌላ</td><td></td></tr>
<tr><td>128</td><td>ቤተሰቡ የኤሌክትሪክ አገልግሎት ያገኛል</td><td>1 = አዎ
2 = አይ</td><td>|__|</td></tr>
</table>

<table>
<tr><td rowspan="12">ቤተሰቡ ከተገለጡት የትኛውን እቃዎች አሉዋቸው?</td><td></td><td colspan="2">ብዛት ቁጥር ይግባ (ምንም ከሌለ 0 የጻፍ)</td></tr>
<tr><td>129</td><td>የእጅ ሰዓት</td><td>|__||__|</td></tr>
<tr><td>130</td><td>ወርቅ በግራም</td><td>|__||__|</td></tr>
<tr><td>131</td><td>የላምባ መብራትወይም ፋኖስ በእምቃህይል በሚገኝ መብራት</td><td>|__||__|</td></tr>
<tr><td>132</td><td>አልጋ</td><td>|__||__|</td></tr>
<tr><td>133</td><td>ተንቀሳቃሽ ያልሆነ (የቤት) ስልክ</td><td>|__||__|</td></tr>
<tr><td>134</td><td>ተንቀሳቃሽ (ሞባይል) ስልክ</td><td>|__||__|</td></tr>
<tr><td>135</td><td>ብስኪሌት</td><td>|__||__|</td></tr>
<tr><td>136</td><td>መኪና</td><td>|__||__|</td></tr>
<tr><td>137</td><td>ራዲዮን</td><td>|__||__|</td></tr>
<tr><td>138</td><td>ቴሌቪዥን</td><td>|__||__|</td></tr>
<tr><td>139</td><td>ማቀዝቀዣ (ፍሪጅ)</td><td>|__||__|</td></tr>
<tr><td>140</td><td colspan="2">የሚኖሩበት ቤት የራሶ ነው?</td><td>1 = አዎ
2 = አይ</td><td>|__|</td></tr>
<tr><td>141</td><td colspan="2">ከቤተሰቡ ውስጥ የእርሻ መሬት ይዞታ ያለው ሰው አለ?</td><td>1 = አዎ
2 = አይ (ወደ 143)</td><td>|__|</td></tr>
<tr><td>142</td><td colspan="2">የዚህ ቤተሰብ አባላት ስንት ሄ/ር የእርሻ መሬት አላውው?</td><td>ጠቅላላ ስፋቱን በሄክታር አመልክት (ከአንድ በታች ከሆነ በክፍልፋይ ይጻፍ ፤ለምሳሌ 0.5)

ስፋቱ የማይታወቅ ከሆነ 9999 ይፃፍ</td><td>|__||__|.|__||__|</td></tr>
<tr><td>143</td><td colspan="2">ይህ ቤተሰብ የሚያረባው እንስሳት፤የዶሮ እርባታ ወይንም የእርሻ አገልግሎት የሚሰጡ እንስሳት አለው?</td><td>1 = አዎ
2 = አይ ወደ ክፍል 2</td><td>|__|</td></tr>
<tr><td rowspan="10">ከሚከተሉት ውስጥ ይህ ቤተሰብ የትኛውን የእንስሳት ዓይነት አለው?</td><td colspan="3">ለእያንዳንዱ: ቁጥር ይጻፍ ፤ምንም ከሌለ 0 ይጻፍ</td></tr>
<tr><td>144</td><td>ዶሮ</td><td>|__|__|__|</td></tr>
<tr><td>145</td><td>ፊየል</td><td>|__|__|__|</td></tr>
<tr><td>146</td><td>በግ</td><td>|__|__|__|</td></tr>
<tr><td>147</td><td>አህያ</td><td>|__|__|__|</td></tr>
<tr><td>148</td><td>ፈረስ</td><td>|__|__|__|</td></tr>
<tr><td>149</td><td>በቅሎ</td><td>|__|__|__|</td></tr>
<tr><td>150</td><td>ግመል</td><td>|__|__|__|</td></tr>
<tr><td>151</td><td>የወተት ላም</td><td>|__|__|__|</td></tr>
<tr><td>152</td><td>በሬ</td><td>|__|__|__|</td></tr>
</table>

ለጥናቱ (እድሜያቸው ከ13-49) የማያሟሉ ሴቶች የቤተሰብ ዝርዝር ውስጥ ከሌሉ መጠይቁ እዚህ ጋር ያበቃል።

ክፊል 2. ጥናቱ ውስጥ ሊካተቱ የሚችሉ ሴቶች

ጠያቂው፡ ይህ መጠየቅ የሚጠየቀው ዕድሜአቸው ከ13-49 *ለሆኑ ተመዝጋቢ ሴቶች ነው፡፡* የቤተሰብ **ዝርዝር** ውስጥ ከተያቼ ቁጥር 118 የተመዘገቡትን ሁሉ ሴቶች እነዳሉ ጠይቁና በእድሜ ትልቅ ከሆነችው ይጀምሩ

ለተመዘገቡት ሴቶች ከመጠየቃቸው በፊት ለአያንዳንዳቸው የተሳትፎ ስምምነት መረጃ ቅፅ ጥያቄዎችን (እስከ 204) በቅደም ተከተል ይሞላ፡፡ መጠይቁን መጨረስ ካልተቻለ ምክናያቱ ይገለጻና ቀጠሮ ይያዝ

የጉብኝት ጊዜ	1ኛ	2ኛ	3ኛ
ቀን/ወር/ዓመት	|__|__| / |__|__| / |__|__|__|__|	|__|__| / |__|__| / |__|__|__|__|	|__|__| / |__|__| / |__|__|__|__|
የጠያቂው ሥም			
ውጤት(ተገቢው መለያ ይግባ)	|__|	|__|	|__|
መጠይቅ በዕለቱ ካልተጠናቀቀ ቀጣዩ ጊዜ ቀጠሮ ቀን ይያዝ *ቀን/ወር/ዓመት*	|__|__| / |__|__| / |__|__|__|__|	|__|__| / |__|__| / |__|__|__|__|	
ጠያቂው፤ መጠይቁ ካላለቀ ቀጣዩ የቀጠሮ ሰአት መች ይሆናል(ቀጠሮ ሰአት ይውሰዱ)			

የውጤት መለያ
1.. የተማላ
2. በግማሽ የተማላ
3. የተላለፈ
4. ቤት ውስጥ ያልተገኙ
5. መጠይቁን ለመመለስ የማይችሉ
6. ፈቃደኛ አየደሉም

በሦስተኛው ዙር መጠይቁ ማጠናቀቅ ካልተቻለ ወደ ምትቀጥለዋ ሴት መጠይቁን ይቀጠሉ ወይንም ወደ ሚቀጥለው ቤተሰብ ይሂዱ

መጠይቁን ሲያካሂዱ ከተቻለ ለብቻ ለማነጋገር ይሞክሩ

200	**ጠያቂ ው/ዋ፡ የመጀምሪዋን ሴት ስም መዝገብ/ቢ**	|__|__|__|__|__|__|__|__|__|__|__|__||__|__|__|	
201	**ጠያቂ ው/ዋ፡ የሴተዋን መለያ ቁጥር መዝገብ/ቢ (ጥምርት/ጉድኝት(cluster) መለያ ቁጥር / ቤተሰብ መለያ ቁጥር /ሴት መለያ ቁጥር)**	|__|__|__| |__|__| |__|__| ጥምርት/ጉድኝት(cluster) ቤተሰብ ሴት	
202	የጠያቂ ው/ዋ ስም	________________________________	
203	**ጠያቂው፤ የተሳትፎ መረጃ ቅጽ ተነቦላታል?**	1 = አዎ<br>2 = አይ	|__|
204	***ጠያቂው፤ ሴትዮዋ ተስማምታለች?***<br>**አይ ከሆነመጠይቁ እዚህ ጋር ያበቃ፡፡ካሉ ሌሎች ዕድሜያቸው ከ13-49 የሆኑ ሴቶች ከክፈል 2 ጀምሮ በአዲስ መጠይቅ ይጠይቁ፡፡፡**	1 = አዎ<br>2 = አይ	|__|
ለታሳትፎሽ አናመሰግናለን፡ አሁን ስለ ጤና ልማት ሠራዊት ልጠይቅሽ አፈልጋለው።			
205	ጤና ልማት ሠራዊት ውስጥ (1ለ 5 መሪ) ነሽ?	1 = አዎ<br>2 = አይ (ወደ 207)	|__|
206	ጤና ልማት ሠራዊት ቡድን (1ለ 30 መሪ) መሪ ነሽ?	1 = አዎ<br>2 = አይ	|__|

አሁን ልጠይቆት የፈለኩት ስላለፈው ጊዜያት እርግዝናዎ ነው፡፡			
207	ባሁኑ ሰዓት ነፍሰ ጡር ነዎት?	1 = አዎ<br>2 = አይ	|__|
208	ከዚህ ቀደም ነፈሰ ጡር ሁነው ያውቃሉ?	1 = አዎ<br>2 = አይ (መጠይቁን ያበቁ)	|__|
209	ከዚህ ቀደም ልጅ ወልደሽ ታውቂያለሽ?	1 = አዎ<br>2 = አይ	|__|
210	ባጠቃላይ ስንት ልጆች ወልደሻል? (ማደጎ ልጆችን አያካትትም)	ቁጥሩን አስገባ/ቢ	|__||__|
211	*ስለ ተውለዱው የሞቱ ህጻናት ወይንም ስለተቋረጠ እርገዝናውች ማውራት በጣም ከባድ እነደሆን እረዳለው ነገር ግን ስለሁሉም ያጋጠመዎት እክሎች ብትነገሪን መንግስት ለህፃናት ጤንነት ላማሻሻል የሚያረገውን ጥረት ይረዳል/ያግዛል።*<br><br>የተቋረጡ ጽንሶች አጋጠሞት ያውቃል?	1 = አዎ<br>2 = አይ (ወደ 213)	
212	ከእርግዝናዎችሽ ስንቶቹ ጽንሶች በህይወት አልተወለዱም?	ቁጥሩን አስገባ/ቢ	|__||__|
213	***አሁን ከ መስከረም 2004 ጀምሮ ስለነበሩ እርግዝናዎች ልጠይቅሽ አፈልጋለው፤፤***<br><br>በመስከረም 2004 ወይንም ከዛ በሁዋላ ነፈሰ ጡር ነበርሽ?	1 = አዎ<br>2 = አይ (መጠይቁን ያበቁ)	|__|

አሁን ከ2004 ጀምሮ ስለነበሩ እርግዝናዎች በሙሉ ልጠይቅሽ አፈልጋለው፤ እሄን ስል ሁልንም እርገዝናውችን ያጠቃልላል፤፤ በህወትም ያሉትን ፤ የሌሉትንም ጨምሮ፤ በሙሉ ዘጠኝ ወረ ድረስ ያጋጠመሽንም ጨምሮ፤ በህወትም ያሉ የሌሉትንም ጨምሮ፤ አሁን ህጻኑ ካንቺም ጋር ወይንም ከሌላም ሱው ጋር ቢኖሩም ንገሪኝ፤፤

ጠያቂው፤ መጠይቁን ከመጀመራቹ በፊት

ሀ) ሴቲቱን ያላትን ሁሉ የውልደት ካረድ ካላት እነድታመጣ ይጠይቁ

ለ) በቅርብ ጊዜ ከነበሩው የዕርግዝና ውት ይጀምሩና ወደ ሁዋላ እስከ መስከረም 2004 የቀጥሉ ፤፤መጠይቁን በምታካሂዱበት ጊዜ እርጉዝ ከሆነች ሴትየዋ ዝርዝሩ ውስጥ አትገባም- እረገዝናው ያበቃ ብቻ ነው የሚዘረዘረው

መንታ ውልድት ወይንም ሁለት ውልደት ካለ፤ በተለያይ ሰንጠረዝ ውስጥ ይመዝገብ (ማለትም የተለያዩ መስመር ውስጥና በተለያይ መለያ ቁጥር)

ጠያቂው፤ ከዘጠኝ ወር በፊት የጠፋ እርገዝና ሲባል መወለድ የነበረበት ጽነስ ከጊዜው በፌት ሲወጣ ነው፡ እናትይው ሳታምጥ የወጣ ማለት ነው፤፤

የእርግዝና መለያ ቁጥር	የእርግዝናው ውጤት	*የሕጻኑ ሥም*	የትውልድ ጊዜ/እርግዝናው ያበቃበት ጊዜ	*መንታ ሆነው የተወለዱ*	*ፆታ*	*እስከ አሁን በሕይወት አለ/ች*	በሕይወት ካሉ እስከ አለፈው ወር ድረስ ዕድሜው/ዋ ስንት ይሆናል	ሕጻና በሕየወት ካለፈ/ች፤ መቼ ነበር
ከቅርቡ ከነበረው ነፍሰጡርነት ይጀምሩ	1 = በሕይወት የተወለደ/ች<br>2 =በሕይወት ያልተወለደ<br>3=ከዘጠኝ ወር በፌት የጠፋ እርገዝና	*ሥም* ከልወጣ አልወጣለት ም ተብሎ ይመዝገብ	ቀኑ ካልታወቀ 01 ያስገቡ (ለማይታወቅ ወር/ዓመት እንደምንም ለማውጣጣት ይሞክሩ)	1 = አዎ<br>2 = አይ<br>3 = አይታወ ቅም	1=ወንድ<br>2 =ሴት<br>3 = አይታወ ቅም	1 = *አዎ*<br>2 = *ላይ*	ከ 28 ቀናት ከሁን በቀናት ይፃፍ	*ላሊታወቀ ጊዜ/ቀን 01 ያስገቡ* (ለማይታወቅ ወር/ዓመት እንደምንም ለማውጣጣት ይሞክሩ)
1	|__|		*ቀን* |__|__| *ወር* |__|__| *ዓመት*|__|__|__|__|	|__|	|__|	|__|	*ቀን* |__|__| *ወር*|__|__|	*ቀን* |__|__| *ወር* |__|__| *ዓመት*|__|__|_ _|__|
2	|__|		*ቀን* |__|__| *ወር* |__|__| *ዓመት*|__|__|__|__|	|__|	|__|	|__|	*ቀን* |__|__| *ወር*|__|__|	*ቀን* |__|__| *ወር* |__|__| *ዓመት*|__|__|_ _|__|
3	|__|		*ቀን* |__|__| *ወር* |__|__| *ዓመት*|__|__|__|__|	|__|	|__|	|__|	*ቀን* |__|__| *ወር*|__|__|	*ቀን* |__|__| *ወር* |__|__| *ዓመት*|__|__|_ _|__|
4	|__|		*ቀን* |__|__| *ወር* |__|__| *ዓመት*|__|__|__|__|	|__|	|__|	|__|	*ቀን* |__|__| *ወር*|__|__|	*ቀን* |__|__| *ወር* |__|__| *ዓመት*|__|__|_ _|__|
5	|__|		*ቀን* |__|__| *ወር* |__|__|	|__|	|__|	|__|	*ቀን* |__|__| *ወር*|__|__|	*ቀን* |__|__| *ወር* |__|__| *ዓመት*|__|__|_

			ዓመት|__|__|__|__|					_|__|
6	|__|		ቀን |__|__| ወር |__|__| ዓመት|__|__|__|__|	|__|	|__|	|__|	ቀን |__|__| ወር|__|__|	ቀን |__|__| ወር |__|__| ዓመት|__|__|_<br>_|__|

**አሁን በነገርሽኝ መሰረት ከመስከረም 2004 ጀምሮ የነበሩ የእርገዝና ጊዜ ማረጋጋጥ አፈልጋለው፤፤**			
214	በህወት የተወለዱ ህጻናት ድምር ቁጥር= |__|	1 = አዎ<br>2 = አይ	|__|
215	ሞተው የተወለዱ ድምር ቁጥር = |__|	1 = አዎ<br>2 = ሏይ	|__|
216	የጠፉ እርግዛናዎች ድምር ቁጥር= |__|	1 = አዎ<br>2 = ሏይ	|__|

ማስታወሻ፡-

መረጃ ላይ ድመሩ ትክክል ካልመጣ ጠያቂው የእርግዝናውን ታረክ አነዲስተካከል እናትይውን ጠይቆ ማጣራት አለበት

ከ መስከረም 2004 ጀምሮ ለነበረ ሁሉ እረግዝና እንደነበረ ከተመዘገበ መጠይቁን ይቀጥሉ፡-

<u>ሞድዩል 3</u>

ክፈል 3› የእርግዝና መለያ ቁጥር

ጠያቂ: ስለሚጠየቀው ህጻን መረጃ ከላይ ከሚገኘዉ ከእርግዝና ውጤት ዝርዝር ሰንጠረዥ ላይ ይውሰዱ፡፡

300	**የህፃኑ ስም (በህይወት የተወለደ)**	|__|__|__|__|__|__|__|__|__|__|__|__| በሕይወት ያልተወለደ ከሆነ/ስም ካልተሰጠ 99 ይፃፍ
301	**የእርግዝና መለያ ቁጥር**<br><br>*የጥምርት/ጉድኝት (cluster) መለይ ቁጥር ከጥያቄ107፡፤*<br>*የቤት መለያ ቁጥር ከጥያቄ 108፤፤*<br>*የሴትየዋን መለያ ቁጥር ከ ከጥያቄ 118 እንዲሁም*<br>*የእርግዝናውን መለያ ቁጥር ከእርግዝና ሰንጠረዝ ይሙሉ*	|__|__|__| |__|__| |__|__| |__|__| <br>ጥምርት/ጉድኝት ቤተሰብ የሴትየዋ የእርግዝናዉ<br>(cluster)

ክፈል 4. ቅድመ ወሊድ እንክብካቤ (ANC)

ጠያቂው፡ የእርግዝናው ውጤት ሞቶ ከሆነ ወይንም ፅንሱ ያለጊዜው ከጠፋ በስም ፋንታ የእርግዝና መለያ ቁጥር ይጠቀሙ፡፡አሁን ስለ (የህጻኑ ስም/እርግዝና ቁጥር) እርግዝናሽ ልጠይቅሽ እፈልጋለወየፃኑን ስም ለማግኘት ክፍል ሁለት ላይ ለተዘረዘረው እርግዝና ውጤት ዝርዝር ይመልከቱ

<u>መፍቻ:-</u>
ጤልሠ- ጤና ልማት ሠራዊት
ጤኤሠ-ጤና ኤክስቴንሽን ሠራዊት

400	(በህጻኑ ስም/እርግዝና ቁጥር) በእርግዝናዎ ወቅት ስለማርገዝዎ ከቤተሰብዎ ውጪ ለሌላ ሰው ተናግረው ነበር ?	1 = አዎ<br>2 = አይ (ውደ404)	|__|
401	ለጤና ባለሙያ (ሠራተኞች) ከተናገሩ ለመጀመሪያ ጊዜ የገለጹትለማን ነው ?	1 = ጤልሠ<br>2 = ጤኤሠ<br>3 = ለሌላ የጤና ባለሙያ(...ነርስ)<br>4 = ሌላ	|__|
402	ሌላ ካለ (ይገለጽ)	ይገለጽ ________________________________	
403	ማርገዝዎን ለጤልሠ'/ጤኤሠ ወይንም ለሌላ የጤና ባለሙያ ሰራተኛ ሲናገሩ የስንት ወር እርጉዝ ነበሩ?	የሰምንታት ቁጥር ያስገቡ<br>፤የማይታወቅ ከሆነ 99 ይፃፍ	|__|__|

404	ስለእርግዝና ክትትልና ስለ ወሊድ የሚገልጽ የእናትና የጨቅላ ህጻን የጤና ካርድ አለዎት ?	1 = አዎ<br>2 = አይ (ወደ 406)	|__|
405	**አዎ ከሆነ** ፡ የእናትና የጨቅላ ህጻን የጤና ካርድ ማየት እችላለሁ?<br>**ጠያቂው፡ የእናትና የጨቅላ ህጻን የጤና ካረድ አለ?**	1 = አዎ<br>2 = አይ	|__|
406	(የህጻኑ ስም/የእርግዝና ቁጥር) ባረገዙበት ወቅት የእርግዝና ክትትል አድረገዋል?<br><br>**ያውጣጡ፤በጤና ኬላ ፣ጤና ጣቢያ፣ ወይንም በጤኤሠ/ጤልሠ በኩል ቤታቸው ተጎብኝተዉ ከሆነ**<br><br>**አይ ከሆነ ቅድመ ወሊድ እንክብካቤ (ANC) ዘለው ወደ ክፈሉ 6 ይለፉ**	1 = አዎ<br>2 = አይ (ወደ ክፈል 6)	|__|

አዎ ከሆነ፤ የቅድመ ወሊድ እንክብካቤን (ANC) ጉብኝት የት የት ነው ያገኙት?<br>የሚመልሱትን ሁሉ መልሶች የመዝገብ<br>**የተባለውን ሁሉ ይሙሉ**	ለእያንዳንዱ: 1 = አዎ 2 = አይ		
	407	ቤት	|__|
	408	ጤና ኬላ	|__|
	409	ጤና ጣቢያ	|__|
	410	ሆስፒታል	|__|
	411	ሌላ(ይገለጽ)	|__|
	412	ይገለጽ_____________________	

ቅድመ ወሊድ እንክብካቤ ከጤና ኬላ

413	በጤና ኬላ ቅድመ ወሊድ / የእርግዝና ክትትል አድርገዋል?	1= አዎ<br>2 =አይ (ወደ 422)	|__|
414		ስንት ጊዜ እንደሄዱ ይመዝግቡ	|__|__|

	በዚያ እርግዝና ወቅት ስንት ጊዜ ለቅድመ ወሊድ/እርግዝና ክትትል ጤና ኬላ ሄዱ?	የማይታወቅ ከሆነ 99 ይፃፍ	
415	በዚያ አርግዝና ወቅት ለመጀመሪያዉ የቅድመ ወሊድ ክትትል ወደ ጤና ኬላ የሄድሽውመቼ ነበር ?<br><br>**የእናትና የጨቅላ ህጻን የጤና ካርድ ካለ መረጃው ከዚያ ይወሰድ**	ቀን |__|__| ወር |__|__| ዓመት |__|__|__|__| ቀን አመት ወር ካልታወቀ 99/99/9999	
416	**ጠያቂው፤ ለጥያቄ 415 ከእናትየው ነው ወይንስ ከካርዱ ነው የተገኘው?**	1 = ከእናትየው<br>2 = ከካርዱ	|__|
417	በዚያ አርግዝና ወቅት ለመጀመሪያ ጊዜ ጤና ኬላ ሲጎበኙ በግምት የስንት ወር ነፍሰጡር ነበሩ?	የሳምንታ ቁጥር ይመዝገብ<br>የማይታወቅ ከሆነ 99 ይፃፍ	|__||__|
418	**ጠያቂው፤ የመጀመሪያ ቅድመ ወሊድ የተጎበኙት ጊዜ ላይ ስለጽንሱ ጊዜ የተጻፈ ቁጥር ካለ ይፃፍ**	ቁጥር በሳምንታት ይመዝገብ<br>ከሌለ 99 የሞላ	|__||__|
419	ከጤና ኬላ ባገኙት የቅድመ ወሊድ እንክብካቤ እረክተዋል ወይንስ አልረኩም?<br>**ምርጫውን አያንብቡላቸዉ**	1 = አዎ እረክቻለው<br><br>2 = አይ አልረካሁም (ወደ 421)<br>3 = እረክቻላሁም አልረካሁምም ማለት አልችልም (ውደ 422 ይሂዱ)	|___|
420	**አዎ ከሆነ፤** የእርካታዎ መጠን ምን ያህል ነው?<br>**ሁለቱንም ምረጫ ያንብቡ**	1 = ሙሉ በሙሉ እረክቻለው (ውደ 422 ይሂዱ)<br>2 = በከፊል እረክቻለው (ውደ 422 ይሂዱ)	|___|
421	**አይ ከሆነ፤** ያልረኩበት መጠን ምን ያህል ነው?<br>**ሁለቱንም ምረጫ ያንብቡ**	1 = ሙሉ በሙሉ እልረካሁም<br>2 = በከፊል እልረካሁም	|___|

**ቅድመ ወሊድ እንክብካቤ በጤና ጣቢያ**			
422	በጤና ጣቢያ የቅድመ ወሊድ / የእርግዝና ክትትል አድርገዋል?	1 = አዎ<br>2 = አይ (ወደ 434)	|__|
423	የመጀመሪያዉን የቅድመ ወሊድ ክትትል ያደረጉት በጤና ጣቢያ ነበር?	1 = አዎ<br>2 = አይ	|__|
424	በዚያ እርግዝና ወቅት ስንት ጊዜ ለቅድመ ወሊድ /የእርግዝና ክትትል ወደ ጤና ጣቢያ ሄዱ?	**የሄዱበትን ጊዜ ብዛት ያመልክቱ**<br>የማይታወቅ ከሆነ 99 ይፃፍ	|__||__|

425	በዚያ እርግዝና ወቅት ለመጀመሪያዉ ለቅድመ ወሊድ ክትትል ወደ ጤና ጣቢያ የሄዱት መቼ ነው?<br><br>**ጠያቂው፤ ቀን፤ ወር ና ዓመት ለማውጣጣት ሞክር**	ቀን |__|__| ወር |__|__| ዓመት |__|__|__|__| ቀን አመት ወር ካልታወቀ 99/99/9999	
426	**ጠያቂው፤ የእናትና የጨቅላ ህጻን የጤና ካርድ መከታተያ ካለ ዝርዝሩን ከዚያ ይውሰዱ**	ቀን |__|__| ወር |__|__| ዓመት |__|__|__|__| ካርድ ላይ ከልተገኘ 99/99/9999 የሞላ	
427	በመጀመሪያዉ ቅድመ ወሊድ ክትትል ጉብኝትዎ ወቅት የስንት ጊዜ እርጉዝ ነበሩ?<br><br>**ጠያቂ፤ የእናትና የጨቅላ ህጻን የጤና ካርድ መከታተያ ካለ ዝርዝሩን ከዚያ ይውሰዱ**	**የሰምነታቱ ብዛት ይመዝገብ**<br>ካልተሰጠ 99 የሞላ	|__||__|
428	**ጠያቂ፤ መረጃው የተገኘው ከእናትየው ነው ወይንስ ከካርዱ ነው?**	1 = ከእናትየው<br>2 = ከካርዱ	|__|
429	ለመጀመሪያ ጊዜ ያየዎት ማነው?	1 = ነርስ<br>2 = አዋላጅ ነርስ<br>3 = የጤና መኮንን<br>4 = ሌላ (ከለ ይገለጽ)<br>5 = አላውቅም	|__|
430	ሌላ ካለ (ይገለጽ)	ይገለጽ________________________	
431	ጤና ጣቢያ ባገኙት ቅድመ ወሊድ እንክብካቤ ለይ እረክተዋል ወይንሰ አልረኩም?<br><br>**ምርጫውን አያንቡት**	1 = አዎ እረከቻለው(ወደ 432)<br>2 =አይ አልረካሁም(ወደ 433)<br>3 = እረክቻላሁም አልረካሁምም ማለት አልችልም (ውደ 434)	|___|
432	**አዎ ከሆነ፤** የእርካታቹ መጠን ምን ያህል ነው?<br>ሁለቱንም ምረጫ ያንብቡ	1 =ሙሉ በሙሉ እረከቻለው (ውደ 434 ይሂዱ)<br>2 =በከፊል እረከቻለው (ውደ 434 ይሂዱ)	|___|
433	**አይ ከሆነ፤** ያልረኩበት መጠን ምን ያህል ነው?<br>**ሁለቱንም ምረጫ ያንብቡ**	1 = ሙሉ በሙሉ አልረካሁም<br>2 = በከፊል አልረካሁም	|___|

**ቅድመ ወሊድ እንክብካቤ በቤት**			
434	በቤትዎ ዉስጥ ከጤና ባለሙያ የቅድመ ወሊድ/ የእርግዝና ክትትል ተድረጎሎታል?	1 = አዎ<br>2 = አይ (ወደ 445)	|__|
435	በእርግዝናዎ ወቅት ለስንት ጊዜ ያህል በቤትዎ ዉስጥ በጤና ባለሙያ ተጎብኝተዋል ?	የጊዜውን ብዛት ያስገቡ	|__||__|
436	በእርግዝናዎ ወቅት ለመጀመሪያ ጊዜ በቤት ዉስጥ የተጎበኙት መቼ ነበር?<br><br>**ጠያቂ፤ የእናትና የጨቅላ ህጻን የጤና ካርድ ካለ ዝርዝሩን ከዚያ ይውሰዱ**	ቀን |__|__| ወር |__|__| ዓመት |__|__|__|__| **ቀን ካልተሰጠ 99የሞላ**	

437	**ጠያቂ፤ ለጥያቄ 436 መረጃው ከእናትየው ነው ወይንስ ከካርዱ ነው የተገኘው?**	1 = ከእናትየው<br>2 = ከካረዱ	|__|
438	ለመጀመሪያ ጊዜ በቤት ዉስጥ ሲጎበኙ እርግዝናዎ የስንት ጊዜ ነበር**?**	የስምንታቱን ቁጥር ይመዝግቡ<br>ቀን የማይታወቅ ከሆነ 99 ይፃፍ	|__||__|
439	**ጠያቂ፤ የእናትና የጨቅላ ህጻን የጤና ካርድ ካለ ዝርዝሩን ከዚያ ይውሰዱ**	የሳምንታ ቁጥር ይመዝግቡ<br>**ካልተሰጠ 99 የሞላ**	|__||__|
440	ለመጀመሪያ ጊዜ በቤት ዉስጥ ሲጎበኙ ሊያዮት ወደ ርስዎ የመጣው ማን ነበር**?**	1 = ጤኤሠ<br>2 = ጤልሠ<br>3 = ሌላ	|__|
441	ሌላ ካለ (ይገለጽ)	ይገለጽ___________________________________	
442	በቤትዎ ባገኙት ቅድመ ወሊድ እንክብካቤ ለይ እረከተዋል ወይንሰ አልረኩም?<br>**ምርጫውን አያንቡት**	1 = አዎ እረከቻለው (ወደ 443)<br>2 = አይ አልረካሁም (ወደ 444)<br>3 = እረከቻላሁም አልረካሁምም ማለት አልችልም (ውደ 445 ይሂዱ)	|___|
443	**አዎ ከሆነ፤** የእርካታዎ መጠን ምን ያህል ነው?<br>**ሁለቱንም ምረጫ ያንብቡ**	1 = ሙሉ በሙሉ እረከቻለው (ውደ 445)<br>2 = በከፊል እረከቻለው(ውደ 445)	|___|
444	**አይ ከሆነ፤** ያልረኩበት መጠን ምን ያህል ነው?<br>**ሁለቱንም ምረጫ ያንብቡ**	1 = ሙሉ በሙሉ አልረካሁም<br>2 = በከፊል አልረካሁም	|___|

ጠያቂ፤ አሁን ቅድመ ወሊድ እንክብካቤ ሲደረግሎት ማን አገልግሎቱን እነደሰጦት እጠይቃለው፡፡

የእርግዝናው ውጤት ሞቶ የተወለደ ከሆነ ወይንም ፅንሱ ያለጊዜው ከጠፋ በስም ፋንታ የእርግዝና መለያ ቁጥር ይጠቀሙ፡፡

ይህን ልጅ (የህጻኑ ስም/የእርግዝና ቁጥር) አርግዘው ሳለ ቀጥሎ የተመለከቱትን አግኝተዋቸው የውቃሉ?
(ጠያቂ፡- ይህ በቤት ዉስጥ ፣ በጤና ኬላ ወይም በጤና ጣቢያ የተሰጠ እንክብካቤ ሊሆን ያችላል)

445	ክብደትዎ ተለክቶ ነበር	1 = አዎ<br>2 = ኤይ (ወደ 448)	|__|
446	ክብደትዎን የለካዎት ጤና ባለሞያ ማን ነበር?<br><br>**ካልታወቀ 9 ይሞላ**	1 = ጤልሠ<br>2 = ጤኤሠ<br>3 = ነርስ/አዋላጅ<br>4 = የጤና መኮንን<br>5 = ዳክተር<br>6 = ሌላ	|__|
447	የት ቦታ ነበር አግልግሎቱን ያገኙት?	1 = ቤት<br>2 = ጤና ኬላ<br>3 = ጤና ጣቢያ	|__|

		4 = ሆስፒታል<br>5 = ሌላ	
448	ቁመትዎ ተለክቶ ነበር?	1 = አዎ<br>2 = ኤይ(ወደ 451)	|__|
449	አዎ ከሆነ፤ ለመጀመሪያ ጊዜ አገልግሎቱን የሰጠዎት ማነው?<br><br>**ካልታወቀ 9 ይሞላ**	1 = ጤልሠ<br>2 = ጤኤሠ<br>3 = ነርስ/አዋላጅ<br>4 = ጤና መኮንን<br>5 = ሐኪም<br>6 = ሌላ	|__|
450	የት ቦታ ነበር አግልግሎቱን ያገኙት?	1 = ቤት<br>2 = ጤና ኬላ<br>3 = ጤና ጣቢያ<br>4 = ሆስፒታል<br>5 = ሌላ	|__|
451	ስለ ጡት ማጥባት መረጃ አግኝተው ነበር?	1 = አዎ<br>2 = አይ (ወደ 454)	|__|
452	አዎ ከሆነ፤ ለመጀመሪያ ጊዜ አገልግሎቱን የሰጠዎት ማነው?<br><br>**ካልታወቀ 9 ይሞላ**	1 = ጤልሠ<br>2 = ጤኤሠ<br>3 = ነርስ/አዋላጅ<br>4 = ጤና መኮንን<br>5 = ሐኪም<br>6 = ሌላ	|__|
453	የት ቦታ ነበር አግልግሎቱን ያገኙት?	1 = ቤት<br>2 = ጤና ኬላ<br>3 = ጤና ጣቢያ<br>4 = ሆስፒታል<br>5 = ሌላ	|__|
454	የደም ግፊትዎ ተለክቶ ነበር;<br>**(ይገለጽ፤መለኪያ በላይኛው ክንድዎ ዙሪያ እንደተደረገ)**	1 = አዎ<br>2 = አይ (ወደ 457)	|__|
455	አዎ ከሆነ፤ለመጀመሪያ ጊዜ አገልግሎቱን የሰጠዎት ማነው<br><br>**ካልታወቀ 9 ይሞላ**	1 = ጤልሠ<br>2 = ጤኤሠ<br>3 = ነርስ/አዋላጅ<br>4 = ጤና መኮንን<br>5 = ሐኪም<br>6 = ሌላ	|__|
456	የት ቦታ ነበር አግልግሎት ያገኙት?	1 = ቤት<br>2 = ጤና ኬላ<br>3 = ጤና ጣቢያ<br>4 = ሆስፒታል<br>5 = ሌላ	|__|
457	የሽንት ናሙና ምርመራ አድርገው ነበር?	1 = አዎ<br>2 = አይ(ወደ 460)	|__|
458	ለመጀመሪያ ጊዜ አገልግሎቱን የሰጥዎት ማነው?	1 = ጤልሠ<br>2 = ጤኤሠ	|__|

	**ካልታወቀ 9 ይሞላ**	3 = ነርስ/አዋላጅ<br>4 = ጤና መኮንን<br>5 = ሐኪም<br>6 = ሌላ	
459	የት ቦታ ነበር አግልግሎቱን ያገኙት?	1 = ቤት<br>2 = ጤና ኬላ<br>3 = ጤና ጣቢያ<br>4 = ሆስፒታል<br>5 = ሌላ	|__|
460	የደም ናሙና ለቂጢኝ ምርመራ አድርገው ነበር?	1 = አዎ<br>2 = አይ (ወደ 463)<br>3 = አላውቅም (ወደ 463)	|__|
461	ለመጀመሪያ ጊዜ አገልግሎቱን የሰጠዎት ማነው?<br><br>**ካልታወቀ 9 ይሞላ**	1 = ጤልሠ<br>2 = ጤኤሠ<br>3 = ነርስ/አዋላጅ<br>4 = ጤና መኮንን<br>5 = ሐኪም (ዶክተር)<br>6 = ሌላ	|__|
462	የት ቦታ ነበር አግልግሎቱን ያገኙት?	1 = ቤት<br>2 = ጤና ኬላ<br>3 = ጤና ጣቢያ<br>4 = ሆስፒታል<br>5 = ሌላ	|__|
463	ሰውነት ውስጥ የሚገኘን የብረት ኜጥረ ነገር አጎልባች ክኒን/ሽሮፕ ና ፎሌት ወስደዋል<br><br>**ጠያቂ፡ የብረት ኜጥረ ነገር አጎልባች ክኒን/ሽሮፕ ና ፎሌት ፎቶ ወይም ናሙና አሳይ**	1 = አዎ<br>2 = አይ(ወደ 467)	|__|
464	ማነው ለመጀመሪያ ጊዜ አገልግሎቱን የሰጠዎት<br><br>**ካልታወቀ 9 ይሞላ**	1 = ጤልሠ<br>2 = ጤኤሠ<br>3 = ነርስ/አዋላጅ<br>4 = ጤና መኮንን<br>5 = ሐኪም (ዶክተር)<br>6 = ሌላ	|__|
465	የት ቦታ ነበር አግልግሎቱን ያገኙት?	1 = ቤት<br>2 = ጤና ኬላ<br>3 = ጤና ጣቢያ<br>4 = ሆስፒታል<br>5 = ሌላ	|__|
466	**አዎ ከሆነ፤** ለስንት ጊዜ ነዉ ኪኒኑን/ሽሮፑን የወሰዱት?	የቀናቱትን ብዛት ይመዝግቡ፤<br>**ከላስታወሱት999ን ይመዝግቡ**	|__||__||__|
467	ህጻኑ ከተወለደ በሁዋላ የመንጋጋ ቆልፍ እንዳይይዘዉ ክንድዎ ላይ ተገቢውን መርፌ ተወግተው ነበር?<br><br>(ለቴታነስ ክትባት ማለት)	1 = አዎ<br>2 = አይ (ወደ 473)	|__|
468	**አዎ ከሆነ፤** ስንት ጊዜ መርፌውን ወሰዱ?	ጊዘውን ብዛት ይመዝግቡ	|__||__||__|

469	**ከሁለት ጊዜ በታች ከሆነ፤** ከዚህ እርግዝና ጊዜ በፊት የቴታነስ መርፌ ተወግተው ያውቃሉ?	1 = አዎ<br>2 = አይ (ወደ 473)	|__|
470	**ጥያቄ 469 አዎ ከሆነ ፡**ከዚህ እርግዝና በፊት ስንት ጊዜ የቴታነስ መርፌ ወስደዋል?	የጊዜውን ድግግሞሽ ይመዝግቡ ቁጥሩ የማይታወቅ ከሆነ 99 ይፃፍ	|__||__|
471	**ጥያቄ 469 አዎ ከሆነ** ከዚህ እርግዝና በፊት ከስንት ዓመት በፊት ነው ለመጨረሻ ጊዜ ይህን የቲታነስ መርፌ የወሰዱት?	ጊዜውን በዓመታት ይመዝግቡ	|__||__|
472	በየትኛው ጤና ተቋም ነበር አግልግሎቱን ያገኙት?	1 = ቤት<br>2 = ጤና ኬላ<br>3 = ጤና ጣቢያ<br>4 = ሆስፒታል<br>5 = ሌላ	|__|
473`	ስለ ኤች አይ ቪ መረጃ አግኝተዋል?	1 = አዎ<br>2 = አይ (ወደ 476)	|__|
474	ከማነው ለመጀመሪያ ጊዜ አገልግሎቱን ያገኙት?<br>**ካልታወቀ 9 ይሞላ**	1 = ጤልሠ<br>2 = ጤኤሠ<br>3 = ነርስ/አዋላጅ<br>4 = ጤና መኮንን<br>5 = ሐኪም (ዶክተር)<br>6 = ሌላ	|__|
475	የት ቦታ ነበር ይህን አግልግሎት ያገኙት?	1 = ቤት<br>2 = ጤና ኬላ<br>3 = ጤና ጣቢያ<br>4 = ሆስፒታል<br>5 = ሌላ	|__|
476	ኤች አይ ቪ ምርመራ አግኝተዋል?	1 = አዎ<br>2 = አይ (ወደ 479)	|__|
477	ከማነው ለመጀመሪያ ጊዜ አገልግሎቱን ያገኙት?<br><br>**ካልታወቀ 9 ይሞላ**	1 = ጤልሠ<br>2 = ጤኤሠ<br>3 = ነርስ/አዋላጅ<br>4 = ጤና መኮንን<br>5 = ሐኪም (ዶክተር)<br>6 = ሌላ	|__|
478	የት ቦታ ነበር አግልግሎቱን ያገኙት?	1 = ቤት<br>2 = ጤና ኬላ<br>3 = ጤና ጣቢያ<br>4 = ሆስፒታል<br>5 = ሌላ	|__|
479	የአባላዘር በሽታ ምርመራ አድረገዋል?	1 = አዎ<br>2 = አይ (ወደ 482)	|__|
480	ከማነው ለመጀመሪያ ጊዜ አገልግሎቱን ያገኙት?<br><br>**ካልታወቀ 9 ይሞላ**	1 = ጤልሠ<br>2 = ጤኤሠ<br>3 = ነርስ/አዋላጅ<br>4 = ጤና መኮንን<br>5 = ሐኪም (ዶክተር)<br>6 = ሌላ	|__|

481	የት ቦታ ነበር አግልግሎቱን ያገኙት?	1 = ቤት<br>2 = ጤና ኬላ<br>3 = ጤና ጣቢያ<br>4 = ሆስፒታል<br>5 = ሌላ	|__|
482	የአባላዘር ሕክምና አግኝተዋል?	1 = አዎ<br>2 = አይ (ወደ 485)	|__|
483	ከማነው ለመጀመሪያ ጊዜ አገልግሎቱን ያገኙት?<br><br>**ካልታወቀ 9 ይሞላ**	1 = ጤልሠ<br>2 = ጤኤሠ<br>3 = ነርስ/አዋላጅ<br>4 = ጤና መኮንን<br>5 = ሐኪም (ዶክተር)<br>6 = ሌላ	|__|
484	የት ቦታ ነበር አግልግሎቱን ያገኙት?	1 = ቤት<br>2 = ጤና ኬላ<br>3 = ጤና ጣቢያ<br>4 = ሆስፒታል<br>5 = ሌላ	|__|
485	ስለ ሥርዓተ ምግብ መረጃ አግኝተዋል?	1 = አዎ<br>2 = አይ (ወደ 488)<br>3 = አይመለከትም (ወደ 488)	|__|
486	ከማነው ለመጀመሪያ ጊዜ አገልግሎቱን ያገኙት?<br><br>**ካልታወቀ 9 ይሞላ**	1 = ጤልሠ<br>2 = ጤኤሠ<br>3 = ነርስ/አዋላጅ<br>4 = ጤና መኮንን<br>5 = ሐኪም (ዶክተር)<br>6 = ሌላ	|__|
487	የት ቦታ ነበር አግልግሎቱን ያገኙት?	1 = ቤት<br>2 = ጤና ኬላ<br>3 = ጤና ጣቢያ<br>4 = ሆስፒታል<br>5 = ሌላ	|__|
488	ሊከሰቱ ስለሚችሉ አደገኛ ምልክቶች መረጃ አግኝተዋል?	1 = አዎ<br>2 = አይ (ወደ 491)	|__|
489	ከማነው ለመጀመሪያ ጊዜ አገልግሎቱን ያገኙት?<br><br>**ካልታወቀ 9 ይሞላ**	1 = ጤልሠ<br>2 = ጤኤሠ<br>3 = ነርስ/አዋላጅ<br>4 = ጤና መኮንን<br>5 = ሐኪም (ዶክተር)<br>6 = ሌላ	|__|
490	የት ቦታ ነበር አግልግሎቱን ያገኙት?	1 = ቤት<br>2 = ጤና ኬላ<br>3 = ጤና ጣቢያ<br>4 = ሆስፒታል<br>5 = ሌላ	|__|

491	ስለ ቅደመ ወሊድ ዝግጅትና ከወሊድ ጋር ተያያዥ ሆነዉ ሊከሰቱ ስለሚችሉ ችግሮች ምክር አግኝተው ነበር?<br><br>***ይገለጽ፣ገንዘብ፤ ማን እነደሚረዳት ፤ መጓጓዣ፤ ላይ ጠይቅ በወሊድ ጊዜ ድጋፍ ለመጓጓዣ በአደጋ ጊዜ ድጋፍ***	1 = አዎ<br>2 = አይ (ወደ 501)	|__|
492	ከማነው ለመጀመሪያ ጊዜ አገልግሎቱን ያገኙት?<br><br>**ካልታወቀ 9 ይሞላ**	1 = ጤልሠ<br>2 = ጤኤሠ<br>3 = ነርስ/አዋላጅ<br>4 = ጤና መኮንን<br>5 = ሐኪም (ዶክተር)<br>6 = ሌላ	|__|
493	የት ቦታ ነበር አግልግሎቱን ያገኙት?	1 = ቤት<br>2 = ጤና ኬላ<br>3 = ጤና ጣቢያ<br>4 = ሆስፒታል<br>5 = ሌላ	|__|
494	የቅደመ ወሊድ ዘግጅትና ከወሊድ ጋር ተያያዥ ሆነዉ ሊከሰቱ ስለሚችሉ ችግሮች አስመልከቶ የተደረገ ዕቅድና ዝግጂት ካለ ዝግጂቱ ተመዝግቦ ይገኛል?	1 = አዎ<br>2 = አይ (ወደ ከ 501)	|__|
495	ከማነው ለመጀመሪያ ጊዜ አገልግሎቱን ያገኙት?<br><br>**ካልታወቀ 9 ይሞላ**	1 = ጤልሠ<br>2 = ጤኤሠ<br>3 = ነርስ/አዋላጅ<br>4 = ጤና መኮንን<br>5 = ሐኪም (ዶክተር)<br>6 = ሌላ	|__|
496	የት ቦታ ነበር አግልግሎቱን ያገኙት?	1 = ቤት<br>2 = ጤና ኬላ<br>3 = ጤና ጣቢያ<br>4 = ሆስፒታል<br>5 = ሌላ	|__|

ክፈል 5. የቅድመ ወሊድ ክትትል ቀጣይ ክፍል

500	የእናትና የጨቅላ ህጻን የጤና ካርድ ላይ የቅደመ ወሊድ ዘግጅትና ከወሊድ ጋር ተያያዥ ሆነዉ ሊከሰቱ የሚችሉ ችግሮችን አስመልከቶ የተደረገ ዕቅድና ዝግጂት ካለ ዝግጂቱ ተመዝግቦ ይገኛል?<br><br>**ጠያቂ የእናትና የጨቅላ ህጻን የጤና ካረድ ካለ ዝርዝሩመኖሩን ተመልከት/ተመልከቺ**	1 = አዎ<br>2 = አይ<br>3 = የእናትና የጨቅላ ህጻን የጤና ካርድ የለም	|__|

የእርግዝና ወቅት አደገኛ ምልክቶችን በዝርዝር ሊነግሩኝ ይችላሉ?<br>**ጠያቂ፤አይነበብ ፤ የተገለጹትን ብቻ አመልከት** ?<br><br>**ይህ የእናትየውን ዕውቀት ለመመዝን ነው እንጂ ያጋጠማትን ለማወቅ አይደለም።**	ለእያንዳንዱ: 1 = አዎ 2 = አይ		
	501	ከብልት የደም መፍሰስ	|__|
	502	ከባድ የሆድ ቁርጠት	|__|
	503	ከማሕጸን የሚወጣ ፈሳሽ	|__|
	504	ትኩሳት	|__|
	505	ራስ ምታት መደበት ወይንም የአይን መቦዝ	|__|

	506	አስተውሎት ማጣት/ ማቀጥቀጥ	|__|
	507	የእጅና ፊት እብጠት	|__|
	ለእያንዳንዱ: 1 = አዎ 2 = አይ		
የቅድመ ወሊድ ዕቅድና ዝግጁነት ወስጥ ተካቶ የተያዙትን ነገሮች በዝርዝር ሊነግሩኝ ይችላሉ?<br><br>**ጠያቂ፤አይነበብ ፤ የተገለጹትን ብቻ አመልክት**<br>ይህ የእናትየውን ዕውቀት ለመመዝን ነው እንጂ ያጋጠማትን ለማወቅ አይደለም።	508	ገንዘብ	|__|
	509	መጓጓዛ	|__|
	510	በቂ ምግብ	|__|
	511	በወሊድ ጊዜ ተንከባከቢ/አዋላጅን ማወቅ	|__|
	512	የት እንደሚወልዱ ቦታ ማውቅ	|__|
	513	የደም ለጋሽ ስለመዘጋጀቱ/አሰፈላጊ ከሆነ	|__|
	514	ንጹሕ ልብስ	|__|
	515	የወሊድ ጊዜ መከናነቢያ (yemioldebet netsu cherk/mentaf)	|__|
	516	የእጅ መሸፈኛ	|__|
	517	የጥጥ ፋሻ	|__|
	518	ሳሙና	|__|
	519	አዲስ ምላጭ	|__|
	520	የተቀቀለ መቀስ	|__|
	521	የተቀቀለ ክር	|__|
522	የቅድመ ወሊድ ዝግጅት አድርገዋል?<br>***ይገለጽ፦ገንዘብ፤ ማን እነደሚረዳት ፤ መጓጓዣ፤ የአደጋ ግዜ ተጠሪ ማዘጋጀት***	1 =አዎ<br>2 =አይ (ወደ 539)	|__|
	ለእያንዳንዱ: 1 = አዎ 2 = አይ		
**አዎ ከሆነ፡** ለወሊዶዎ ወይም ከመዉልለድዎ በፊት ምን ምን ዝግጅት አድርገው ነበር ?<br><br>**ምርጫዎቹን አያንብቡላቸዉ የሚነገረዎትን ብቻ ይመዝግቡ፡፡**	523	ገንዘብ	|__|
	524	መጓጓዛ	|__|
	525	በቂ ምግብ ማዘጋጀት	|__|
	526	በወሊድ ጊዜ ተንከባከቢ/አዋላጅን ማወቅ	|__|
	527	የት እንደሚወልዱ ቦታ ማውቅ	|__|
	528	ደም ለጋሽ ስለመዘጋጀቱ/አሰፈላጊ ከሆነ	|__|
	529	ንጹሕ ልብስ	|__|
	530	የወሊድ ጊዜ መከናነቢያ	|__|
	531	የእጅ መሸፈኛ	|__|

532	የጥጥ ጥቅል		|__|
533	ሳሙና		|__|
534	አዲስ ምላጭ		|__|
535	የተቀቀለ መቀስ		|__|
536	የተቀቀለ ክር (እትብት ለመቐጠር)		|__|
537	ሌላ (ይገለጽ)		|__|
538	ይገለጽ ______________________________		
539	በመጨረሻ እርግዝናዎ ወቅት በነፍሰጡር ሴቶች ውይይት/ስብሰባ ላይ ተካፍለው ያውቃሉ?	1 = አዎ<br>2 = አይ (ወደ 552)	|__|
540	አዎ ከሆነ፤ ስንት ጊዜ በዚህ የነፍሰጡር ሴቶች ስብሰባ ውስጥ ተሳትፈዋል?	ስንት ጊዜ እነደሆን ይመዝገቡ ቁጥሩ የማይታወቅ ከሆነ 99 ይፃፍ	|__||__|
	የነፍሰ ጡር ሴቶች ስብሰባ ላይ ምን ምን ነገሮች ላይ ነበር ውይይት የተደረገው?<br><br>**አይነበብ ግን በተቻለ መጠን ያውጣጡ**<br>**Indicate all that applay**	ለእያንዳንዱ: 1 = አዎ 2 = አይ	
541	የወሊድ ዝግጅት		|__|
542	የቅደመ ወሊድ እነክብካቤ አስፈላጊነት		|__|
543	በጤና ተቋም ስለ መውለድ		|__|
544	የድህረ ወሊድ ክትትል አስፈላጊነት		|__|
545	የጨቅላ ሕጻናት እንክብካቤ		|__|
546	ሌላ (ይገለጽ)		|__|
547	ይገለጽ______________________________|		
548	ስለ ነፍሰ ጡር ሴቶች ስብሰባ ማን ነገረዎት?	1 = ጤልሠ<br>2 = ጤኤሠ<br>3= ሌላ	|__|
549	ሌላ ካለ እባክዎን ይግለጹ	ይገለጽ________________________	
550	የነፍሰ ጡር ሴቶች ስብሰባ የት ነበረ የተከናወነው?	1 = የጤልሠ ቤት<br>2 = በጎጥ/ቀበሌ ውስጥ<br>3 = ጤና ኬላ<br>4 = ጤና ጣቢያ<br>5 = ሌላ (ይገለጻ)	
551	ሌላ ካለ (ይግለጹ)	ይገለጽ________________________	
552	ነፍሰ ጡር በነበሩበት ወቅት ባገኙት የጤና እንክብካቤ እረክተዋል ወይንስ አልረኩም?<br><br>**ምርጫውን አያንቡት**	1 = አዎ እረክቻለው<br>2 = አይ አልረካሁም (ወደ 554)<br>3 = እረክቻላሁም አልረካሁምም ማለት አልችልም (ውደ 555)	|___|

553	አዎ ከሆነ፤ የእርካታቹ መጠን ምን ያህል ነው?<br><br>**ሁለቱንም ምረጫ ያንብቡ**	1 = ሙሉ በሙሉ እረክቻለው (ውደ 555)<br><br>2 = በከፊል እረክቻለው (ውደ 555)	|___|
554	አይ ከሆነ፤ ያልረኩበት መጠን ምን ያህል ነው?<br><br>**ሁለቱንም ምረጫ ያንብቡ**	1 = ሙሉ በሙሉ እልረካሁም<br>2 = በከፊል እልረካሁም	|___|

**ጠያቂ<br>ተጠያቂዋ ቅድመ-ወሊድ ክትትል በጤና ተቋም ዉስጥ አድርጋ ከነበረ የሚከተሉትን ጥያቄዎች ጠይቅ/ቂ (552-566)**			
ጤና ተቅዋም ውስጥ (ጤና ጣቢያ፤ ጤና ኬላ ወይንም ሆስፒታል) ምርመራ በምታደርጊበት ጊዜ የሚከተሉት አጋጥሞሽ ያውቃሉ?<br><br>**የሚመልሱትን መልሶች ሁሉ መዝግቡ**	ለያንዳንዱ ጥያቄ 1 =አዎ 2 = አይ		
	555	ስለ ወሊድ ያለሽን ጥያቄ እንድትጠይቂ ያበረታታሽ ሰው ነበረ ?	|___|
	556	የምትፈልጊውን የህክምና ምርጫ እንድትመርጪ እድል የሰጠሽ ሰው ነበረ?(በኦፔሬሽን ወይም በተፈጥራዊ አወላለድ)	|___|
	557	ያለአስፈላጊ በ ቀዶ ጥገና (ሲ ሴክሽን) እድትወልጂ የገፋፋሽ ሰው ነበረ?	|___|
	558	የሚደረገውን የህክምና ክንውን ያብራራልሽ ሰው ነበረ? (ለምሳሌ ስለተፈጥሮያዊ አወላለድ ወይንም በቀዶ ጥገና መውለድ ወይንም ነገሮች ምን ያኀል ሰአት እንደሚወስዱ ያብራራልሽ ሰው ነበረ?)	|___|
	559	በምርመራ ጊዜ በተገለለ ቦታ እንድትታይ ያደረገ ሰው ነበረ? (ለምሳሌ በር መዘጋተ ወይንም መጋረጃ መዝጋት)	|___|
	560	በምርመራ ጊዜ አክብሮት በሌለው ቋንቋ ያተናገረሽ ሰው ነበረ?<br>(ለምሳሌ መስደብ ወይንም አንቺን/ ቤተሰብሽን/ ማህበረሰብሽን/ብሄረሰብሽን ማንቋሸሽ)	|___|
	561	ማህበረሰብሽን/ብሄረሰብሽን ምክኒያት በማድረግ የህክምና አገልግሎት የከለከለሽ ህክምና አገልጋይ ነበረ?	|___|
	562	በምርመራ ጊዜ የጮሀብሽ ወይንም የቁጣ ድምፅ የተጠቀሙብሽ ሰው ነበረ?	|___|
	563	በምርራ ጊዜ ትንኮሳ/አላስፈላጊ ቃለት በመናገር የተጠቀሙብሽ ሰው ነበረ?	|___|
	564	ያስፈራራሽ ሰው ነበረ?<br>(ምሳሌ *የምልሽን ካላደረግሽ በቀዶ ጥገና ነው የማዋልድሽ* እያለ ያስፈራረሽ ሰው ነበረ?)	|___|
	565	በምርመራ ጊዜ ያለአግባብ ለብቻሽ ተትተሽ ነበረ?	|___|
	566	የምርመራ ውጤትሽን (Diagnosis) የነገረሽ ሰው ነበረ?	|___|
	567	ሌላ ሰው ሊሰማ በሚችልበት ጊዜ የምርምራሽ ውጤትሽ ተነግሮ ነበር?	|___|
	568	የኀክምና ውጤትሽ/ሬኮርድሽ በድብቅ እንደሚያዝ ያረጋገጠልሽ ሰው ነበረ?	|___|
	569	ገንዘብ ባለሞሟላቱ የጠየቅሽውን የህክምና አገልግሎት የከለከለሽ ሰው ነበረ?	|___|

የእርግዝናው ውጤት (ውጤት ኮድ 3=ከዘጠኝ ወር በፊት የጠፋ እርግዝና) ከሆነ መጠይቁን እዚህ ያብቁና ሌላ እርግዝና እንዳለ ያረጋግጡ።

ክፍል 6፡ ስለ ወሊድ እንክብካቤ

አሁን (የህጻኑ ስም/የእርግዝና ቁጥር) ስትወልጂ ስለነበሩ ሁኔታዎች አንዳንድ ጥያቄዎች ልጠይቅሽ እፈልጋለው።

600	የት ወለድሽ?	1 = ቤት (ወደ 602)<br>2 = የጤና ኬላ (ወደ 609)<br>3 = የጤና ጣቢያ(ወደ 609)<br>4 =ሆስፐታል(ወደ 609)<br>5 = ሌላ	|__|
601	ሌላ ካለ እባክዎን ይግለጹ፣	ሌላ________________________________|	

በመኖሪያ ቤት ውስጥ ከተገላገሉ ለምን?<br><br>**ይሚመልሱትን መልሶች ሁሉ መዝግቡ**		ለእያንዳንዱ: 1 = አዎ 2 = አይ	
	602	ሁሌም የሚወልዱት በቤት ውስጥ ስለሆነ	|__|
	603	ባል/እናት/የባል እናት ወደ ጤና ተቋም እንድሄድ አይፈቅዱም	|__|
	604	የጤና አገልግሎት ሰጪ ተቋማትን አይወዱም	|__|
	605	የጤና አገልግሎት ማግኘት ውድ ነው	|__|
	606	በሕላዊ/ሐይማኖታዊ በሆኑ ምክንያቶች	|__|
	607	ሌላ	|__|
	608	ይገለጽ____________________________________|	
በጤና ኬላ፣ በጤና ጣቢያ፡ ሆስፒታል ውስጥ ከተገላገሉ ለምን?<br><br>**ይሚመልሱትን መልሶች ሁሉ መዝግቡ**		ለእያንዳንዱ: 1 = አዎ 2 = አይ	
	609	ሁሌም የሚወልዱት በጤና ተቋም ነው	|__|
	610	በእረግዝና ችግር ምክንያት	|__|
	611	በጤልወ/ጤኤሠ ተልከው	|__|
	612	በነፍሰጡር ሴቶች ስብሰባ አማካይነት ተመክረው ነው	|__|
	613	በምጥ መዘግየት ወይንም በወሊድ ወቅት በተከሰተ ችግር ምክንያት	|__|
	614	ምቹነቱ	|__|
	615	ወጪው አነስተኛ ስለሆነ ወይም ነፃ ስለሆነ	|__|
	616	ሌላ ይገለጽ	|__|
	617	ይገለጽ____________________________________|	

618	በዋናናት በማዋለድ የረዳዎት ሰው ማነው?	1 = ሐኪም (ዶክትር)<br>2 = ነርስ/አዋላጅ<br>3 = ጤኤሠ<br>4 = ጤልሠ<br>5 = የባሕል አዋላጅ<br>6 = ዘመድ/ጓደኛ<br>7 = ማንም<br>8 = ሌላ	|__|

በማገላገሉ ሌላ ማን ተሳተፈ?<br><br>**ይሚመልሱትን መልሶች ሁሉ መዝግቡ**	ለእያንዳንዱ: 1 = አዎ 2 = አይ		
	619	ሐኪም (ዶክትር)	|__|
	620	ነርስ/አዋላጅ	|__|
	621	ጤኤሠ	|__|
	622	ጤልሠ	|__|
	623	የባሕል አዋላጅ	|__|
	624	ዘመድ/ጓደኛ	|__|
	625	ማንም	|__|
	626	ሌላ	|__|

627	በዋናናት በማዋለድ የረዳዎት ሰው ከማወለዳቸው በፊት እጃቸውን በሳሙና ታጥበው ነው?	1 = አዎ<br>2 = አይ<br>3 = አላውቅም	|__|
628	በዋናናት በማዋለድ የረዳዎት ሰው የእጅ ጓንት አድርገው ነበር?	1 = አዎ<br>2 = አይ<br>3 = አላውቅም	|__|
629	ሲወልዱ፣ የተገላገሉበት ቦታ ንጹሕ ነበር? (ንጹሕ ቦታ ማለት፡ ንጹሕ ምንጠፍ ወይንም ንጹሕ ቦታ፤ ጨርቅ፡ ቅጠል)	1 = አዎ<br>2 = አይ<br>3 = አላውቅም	|__|
630	የደም መፍሰስን ለመከላከል የሚረዳ መድሀኒት(ሚሶፕሮስቶል የሚባል ክኒን) ተሰጥቶት ነበር?	1 = አዎ<br>2 = አይ<br>3 = አላውቅም	|__|
631	ጠያቂ፡- በጤና ተቀዋም ውስጥ ከወለደች የሚከተሉት ጠይቃት/ቂያት ፤ ካልሆነ ወደ 633 ዝለል፡<br><br>ከወለዱ በሁዋላ በድምር ለስንት ቀናት በጤና ተቋም ውስጥ ቆዩ?	የቀናቱ ብዛት ያስገቡ<br><br>የወሊደችበት ዕለት ብቻ ከሆነ 0 በማስገባት ይለፉት	|__|__|
632	(የህጻኑ ስም/የእርግዝና ቁጥር) ሲወለድ/ስትወለድ በኦፕራስዮን ነው የተገላገሉት - ማለትም ሆድዎን ቀደው ነው ልጁን ያወጡት?	1 = አዎ<br>2 = አይ	|__|

(የሕጻኑ ሥም/እርግዝና ቁጥር) ሲወለድ ከሚከተሉት ውስጥ አንዱ አጋጥሞት ነበር?<br><br>ዝርዝሩን ያንብቡ፡	ለእያንዳንዱ = አዎ 2 = አይ		
	633	ከፍተኛ የደም መፍሰስ	|__|
	634	ከ12 ሰዓት የረዘመ ምጥ	|__|
	635	አዕምሮ መሳት	|__|
	636	ያለሰአቱ የመጣ ምጥ	|__|
	637	ያልተገባ (ሽታ ያለው) ፍሳሽ	|__|
	638	የሕጻኑ ባልተለመደ መንገድ መምጣት (በጭንቅላቱ ሳይሆን)	|__|

639	በወሊድ ጊዜ የተሻለ አገልግሎት ለማግኘት እንዲችሉ ወደ ከፍተኛ የጤና ተቋም እንዲሄዱ ተመክረው ነበር?	1 = አዎ<br>2 = አይ (ወደ 646)	|__|
640	ለምን ነበር ወደዚያ እንዲሄዱ የተመከሩት?<br><br>**(ጠያቂ፤- እባክዎ የመላኪያ ካርዱን ተመልከተው አደገኛ ሁኔታ ተመዝግቦ የነበረ ከሆነ ያረጋግጡ)**	1 = በአንዱ ወይንም ከዛ በላይ አደገኛ ሁኔታ ምክንያት<br>2 = በሕክምና መሳሪያ እጥረት/ቦታ<br>3 = በሰለጠነ የሰው ሃይል እጥረት<br>4 = ሌላ(ይገለጽ)	|__|
641	ሌላም ካለ ይገለጽ	ይገለጽ______________________________	
642	ወደ ከፍተኛ የጤና ማዕከል/ተቋም እንዲሔዱ በተነገረዎት ጊዜ ወደዚያ ሄደው ነበር ?	1 = አዎ (ወደ645)<br>2 = አይ	|__|
643	አይ ከሆነ ለምን	1 = ማእከሉ ሩቅ ስለነበረ<br>2 = ወጪው ብዙ በመሆኑ<br>3 = ወደተለያየ ጤና መእከል መሄዱን ስላልመረጥኩ<br>4 = ለመሄድ ፍቃድ በማጣት<br>5 = ተቋሙ የሚሰጠውን ክብክባ ባለመውደድ<br>6 = ሌላ	|__|
644	ሌላም ካለ ይገለጽ	ይገለጽ______________________________	
645	ለሪፈራል አምቡላንስ ለማግኘት ችለው ነበር ?	1 = አዎ<br>2 = አይ	|__|
646	በተሰጥዎት የማዋለድ አገልግሎት ረክተዋል ወይስ አልረኩም?<br><br>ጠያቂ: ምርጫዎቹ እንዳይነበቡ።	1 = አዎ እረክቻለው<br>2 = አይ አልረካሁም(ወደ 648)<br>3 = አረክቻለውሁም አልረካሁምም ማለት አልችልም (ወደ649)	|___|
647	መለሱ አዎ ከሆነ የእርካታዎ መጠን ምን ያህል ነበር?<br>ጠያቂ: ምርጫዎቹ ይነበቡ።	1 = ሙሉ በሙሉ እረክቻለው (ወደ649)<br>2 = በከፊል እረክቻለው (ወደ649)	|___|
648	መልሱ አይ ከሆነ፤ ያልረኩበት መጠን ምን ያህል ነበር?	1 = ሙሉ በሙሉ አረካሁም<br>2 = በከፊል አረካሁም	|___|

ጠያቂ፡-
ተጠያቂዋ በጤና ተቋም ውልዳ ከነበረ ብቻ ነው የሚከተሉት ጥያቄዎች የሚጠየቁት (649 - 670)

ጤና ተቅዋም ውስጥ (ጤና ጣቢያ፤ ጤና ኬላ ወይንም ሆስፒታል) ስትወልጂ ከ'ሚከተሉት ያጋጠመሽ ነገር ነበር?<br><br>**ይሚመልሱትን መልሶች ሁሉ መዝግቡ**	ለያንዳንዷ ጥያቄ 1 =አዎ 2 = አይ		
	649	ያለሽበትን ሀኔታና የሚደረግልሽን የህክምና ሂደት በየጊዜው ያብራራና የነገረሽ ሰው ነበር?	|___|
	650	ወደ ወሊድ ክፍል በምትወሰጂበት ጊዜ በደንብ የሸፈነሽ ሰው ነበር?	|___|
	651	ውሳኔ ከተሰጠ በኃላ የህክምና አገልግሎት ዘግይቶብሽ (ምሳሌ ሆድን ቀዶ ልጅን ማውጣት) ነበር?	|___|
	652	ሃይለ ቃል የተጠቀምብሽ ነበር? (በወሊድ ግዜ)	
	653	በወሊድ ጊዜ ያለአግባብ ለብቻሽ ተትተሽ ነበር?	|___|
	654	ሳይነገርሽ ወይንም ሳትፈቅጂ የህክምና እገልግሎት (በቀዶ	|___|

		ጥገና መውለድ ፤ ደም መሰፍሰስ፣ ማህፀን መቋጠር ወዘተ) ተሰጥቶሽ ነበር?	
	655	በወሊድ ጊዜ የህመም ማስታገሻ መድሀኒት ስትጠይቂ ችላ ያለሽ ሰው ነበር?	|___|
	656	በወሊድ ጊዜ የጮሁብሽ ወይንም የቁጣ ድምፅ የተጠቀሙብሽ ሰው ነበር?	|___|
	657	በጥፊ የመታሽ ሰው ነበር?	|___|
	658	በሚያም ሁኔታ የቆነጠጠሽ ሰው ነበር?	|___|
	659	የደበደበሽ ሰው ነበር?	|___|
	660	እድትጠጊ ያላግባብ በሃይል የገፋሽ ሰው ነበር?	|___|
ጣና ኬላ ፤ ጣቢያ ወይንም ሆስፒታል ከወለድሽ በኋላ ከሚከተሉት ያጋጠመሽ ነገር ነበር?<br><br>**የሚመልሱትን መልሶች ሁሉ መዝግቡ**	ለያንዳንዷ ጥያቄ 1 =አዎ 2 = አይ		
	661	ከወለድሽ በኀላ ያለሽን ጥያቄ እንድትጠይቂ ያበረታታሽ ሰው ነበር?	|___|
	662	ከወለድሽ በኃላ በደንብ የሸፈነሽሰው ነበር?	|___|
	663	ወልደሽ ወዲያውኑ ለብቻሽ ተትተሽ ነበረ?	|___|
	664	ልክ እንደወለድሽ ጥለውሽ ሄዴው ነበር ?	
	665	ከወለድሽ በኃላ የጮሁብሽ ወይንም የቁጣ በድምፅ የተጠቀሙብሽ ሰው ነበር?	|___|
	666	ከወለድሽ በኃላ የማንቋሸሽ የሚበድል ቋንቋ የተጠቀሙብሽ ሰው ነበር?	|___|
	667	ከወለድሽ በኃላ የማዋለጃ አልጋውን እንደታፀጂ የጠየቀሽ ሰው ነበር?	|___|
	668	ከወለድሽ በኃላ መፀዳጃ /ሽንት ቤት እንድታፀጂ የጠየቀሽ ሰው ነበር?	|___|
	669	ክፍያ ሙሉ በሙሉ ሰላለተከፈለ ጤና ተቋም ውስጥ ያቆየሽ ሰው ነበር?<br>(ምሳሌ አንቺንና ልጅሽን ሙሉ ክፍያ እስኪከፈል ይዞ ማቆየት)	|___|

670	አስተያየትና ቅሬታ ቢኖርሽ የት ሄዳሽ ማቅረብ እንደምትችይ ታውቂያለሽ?	1 = አዎ<br>2 = አይ	|___|

ክፈል 7. ስለ ድ[illegible]ረ ወሊድ ክብክቤ (PNC)

አሁን በድህረ ወሊድ ስላደረጉት ክትትል ልጠይቅዎ እፈልጋለው፡፡

700	ከወለድሽ ቦኃላ ባሉት 6 ሳምንታት ውስጥ የጤና ክትትል ተደርጎሎሽ ነበር?<br><br>**ጠያቂ: የድህረ ወሊድ አገልግሎት ጤኤሠ/ጤና ጣቢያ ሰራተኛ/ ጤልሠ/ነርስ/ዶክተር አግኝታ እንደነበር ለማውጣጣት ሞክር/ሪ**	1 = አዎ<br>2 = አይ (ወደ ክፈል 8)	|__|

701	ከወለድሽ ከስንት ቀናት በኋላ ነበር የመጀመሪያዉ የጤና ክትትል የተደረገልሽ?<br><br>**ጠያቂ፡ ይህ ጥያቄ ለእናትየው የጤና ክትትል መሆኑን ግልፅ ይሁን።**	የቀኑ ብዛት ይጠቀስ<br>የማይታወቅ ከሆነ 99 ይፃፍ	|__|__|
702	የመጀመሪያው የጤና ክትትል የት ነበር የተካሄደው?	1 = ቤት<br>2 = የጤና ኬላ<br>3 = የጤና ጣቢያ<br>4 =ሆስፒታል<br>5 = ሌላ	|__|
703	በማን?<br><br>**ካልታወቀ 9 ይሞላ**	1 =ሐኪም (ዶክተር)<br>2 =ነርስ/አዋላጅ<br>3 =ጤኤሠ<br>4 = ጤና መኮንን/ረደት<br>5 = ጤልሠ	|__|
704	ከወለድሽ ቦኃላ ሁለተኛ ክትትል ተደርጎሎሽ ነበር?	1 = አዎ<br>2 = አይ (ወደ 712)	|__|
705	ከወለድሽ ከስንት ቀናት በኋላ ነው የሁለተኛውን የጤና ክትትል የተደረገልሽ?<br><br>**ጠያቂ፡ ይህ ጥያቄ ለእናትየው የጤና ክትትል መሆኑን ግልፅ ይሁን።**	የቀኑ ብዛት ይጠቀስ<br>የማይታወቅ ከሆነ 99 ይፃፍ	|__|__|
706	የት ነበር የሁለተኛዉ ጤና ክትትል የተካሄደው?	1 = ቤት<br>2 = የጤና ኬላ<br>3 = የጤና ጣቢያ<br>4 =ሆስፒታል<br>5 = ሌላ	|__|
707	በማን?<br><br>**ካልታወቀ 9 ይሞላ**	1 = ሐኪም (ዶክተር)<br>2 = ነርስ/አዋላጅ<br>3 = ጤኤሠ<br>4 = ጤና መኮንን/ረደት<br>5 = ጤልሠ	|__|
708	ከወለድሽ በኋላ ሶስተኛ ክትትል ተደርጎሎሽ ነበር?	1 = አዎ<br>2 = አይ (ወደ 712)	|__|
709	ከወለድሽ ከስንት ቀናት በኋላ ነው ሶስተኛው የጤና ክትትል የተደረገልሽ?<br>**ጠያቂ፡ ይህ ጥያቄ ለእናትየው የጤና ክትትል መሆኑን ግልፅ ይሁን።**	የቀኑ ብዛት ይጠቀስ<br>የማይታወቅ ከሆነ 99 ይፃፍ	|__|
710	የት ነበር ሦስተኛዉ የጤና ክትትል የተካሄደው?	1 = ቤት<br>2 = የጤና ኬላ<br>3 = የጤና ጣቢያ<br>4 =ሆስፒታል<br>5 = ሌላ	|__|
711	በማን?<br><br>**ካልታወቀ 9 ይሞላ**	1 = ሐኪም (ዶክተር)<br>2 = ነርስ/አዋላጅ<br>3 = ጤኤሠ<br>4 = ጤና መኮንን/ረደት<br>5 = ጤልሠ	|__|

ከወሊድ በኋላ በተደረገልዎ ክትትል ወቅት ምን ምን አገልግሎት ነበር ያገኙት?<br>**ጠያቂ፤ ምርጫዎቹን ይነበቡ።**<br>**የተጠቀሱትን ሁሉ አመልክት፡፡**<br>**በተጠቀሰው ጊዜ ከወሊድ በኋላ ክትትል ካልተደረገ ሰንጠረዡ ውስጥ ምንም አይሞላ**	
	ለእያንዳንዱ: 1 =አዎ 2 =አይ

	(ጥያቄ #)	0-2ቀናት	(ጥያቄ #)	3-7 ቀናት	(ጥያቄ #)	8-42 ቀናት
የጡት ክትትል	712	|__|	713	|__|	714	|__|
ስለ ጡት አመጋገብ ምክር	715	|__|	716	|__|	717	|__|
ስለአደገኛ ምልክቶች ገለጻ	718	|__|	719	|__|	720	|__|
የቤተሰብ እቅድ ምክር አገልግሎት	721	|__|	722	|__|	723	|__|
በስርዓተ ምግብ ላይ የተሰጠ ግንዛቤ	724	|__|	725	|__|	726	|__|
ወደ ጤና ተቋም ተመርተዋል	727	|__|	728	|__|	729	|__|
የደም ግፊት ተለከተዋል	730	|__|	731	|__|	732	|__|
በወሊድ ምክንያት የተከሰተ ቁስለት (ከነበረ) ታይተዋል/ታክመዋል	733	|__|	734	|__|	735	|__|
ሌላ	736	|__|	737	|__|	738	|__|
ሌላ ካለ ይገለጽ___________						

739	በተሰጥዎት የድህረ ወሊድ አገልግሎት ረክተዋል ወይስ አልረኩም?<br><br>**ምርጫዎቹ እንዳይነበቡ።**	1 = አዎ እረክቻለው<br>2 = አይ አልረካሁም(ወደ 741)<br>3 = አረክቻለውሁም አልረካሁምም ማለት አልችልም *(ወደ ክፈል 8)*	|___|
740	**አዎ ከሆነ፡** የእርካታዎ መጠን ምን ያህል ነበር?<br>**ምርጫዎቹ ይነበቡ።**	1 = ሙሉ በሙሉ እረክቻለው *(ወደ ክፍል 8)*<br>2 = በከፊል እረክቻለው *(ወደ ክፈል 8)*	|___|
741	**አይ ከሆነ፤** ያልረኩበት መጠን ምን ያህል ነበር?	1 = ሙሉ በሙሉ አረካሁም<br>2 = በከፌል አረካሁም	|___|

ጠያቂ፡ የእርግዝናው ውጤት ከእርግዝና ሰንጠረዥ (2 =በሕይወት ያልተወለደ(ውይንም ሞቶ የተወለደ) ከሆነ ቃለ መጠይቁን እዚህ አብቃ/ቂና ሌላ እርግዝና መኖሩን አረጋግጥ/ጪ

**ክፍል 8. eK ›Ç=e ¾}¨KÆ Qí"ƒ እን¡w"u?**			
**ከዚህ ቀጥሎ (ስም) በተወለደበት/ችበትና ከዝያ በኋላ ስለነበሩ አንዳንድ ሁኔታዎች ልጠይቅሽ እፈልጋለሁ፤፤**			
800	**ÖÃm& ¾(eU) የእርግዝና ¾SKÁ lØ` ሙላ/ይ**	¾Qí'<"/"" የእርግዝና SKÁ lØ` በ¡ðM 2 ŸK¨< ´[´[›eÑv	|__|
801	(eU) S"ታ ነበር የተወለደዉ/ችዉ ;	1. አዎ<br>2. አይ	|__|
802	(ስም) ሲወለድ/ስትወለድ ኪሎው/ዋ }S´•/" 'u` ¨Ã;	1 = አዎ<br>2 = አይ(ወደ 804)<br>3 = አለውቅም (ወደ 804)	|__|
803	አዎ ከሆነ: (eU) ሲወለድ/ስትወለድ Ÿ=KA¨</ª Uን ያህል ነበር?	ክብደ~"/ª" uÓ^U ይመዝግቡ፡፤<br>ለምሳሌ ክብደቱ/ƒª 1.9 ኪግ ከነበረ 1900 ይመዝግቡ<br>ካሌለ 9999 የፃፍ (ከካርድ ካለ ይመዝገቡ)	|__|__|__|__|
804	(ስም) እንደተወለደ/ች የማልቀስ/የመተንፈስ ችግር ነበረበት/ባት	1 = አዎ<br>2 = አይ(ወደ 807)	|__|

		3-= አላውቅም	

ልክ (ስም) እንደተወለደ/ች የሚከተሉት }Å`Ôለት/ላት ነበር ; **የሚመልሱትን መልሶች ሁሉ ይመዝግቡ**	ለእያንዳንዱ፡ 1 =አዎ 2 =አይ		
	805	Tgf ¨ÃU T'nnf	|__|
	806	ከአፍ ወደ አፍ f"ói SeÖf	|__|

807	(eU) ወዲያውኑ እንደተወለደ የት ነበር ¾}kSÖ¨</ቸ¨<;	1 = ለብቻ/ወለል ላይ<br>2 = በእናት አቅፍ /ደረት<br>3 = ከእናት አጠገብ<br>4 = ከሌላ ሰው ጋር<br>5 = ሌላ<br>6 = አላውቅም	|__|
808	(ስም) ከተወለደ/ች ከምን ደቂቃ በ🕒ላ ነበር c¨<'~"/…" ¾Ö^[Ñ<f/ÁÅ^[lf ;<br><br>**ጊዜው የእንግዴ ልጅ ከመጣ ሳይሆን ሕጻኑ Ÿተወለደ ጀ<ኋL መሆኑን ያረጋግጡ፡፡**	Ñ>²?¨<" uÅmn ÃS<K< ካልታወቀ 999 ይመዝግቡ	|__| |__| |__|
809	(eU) Ÿ}¨KÅ ከምን ያlል ደቂቃ በ🕒ላ ነበር በጨርቅ ¾}ÖkKK¨</ችው ?<br><br>**ጊዜው እንግደ ልጅ ከመጣ ሳይሆን ሕጻኑ Ÿተወለደ ጀ<ኋL መሆኑን ያረጋግጡ፡፡**	Ñ>²?¨<" uÅmn ÃS<K< ካልታወቀ 999 ይመዝግቡ	|__| |__| |__|
810	እትብ~"/…" ለመsጠር U" 'u` የተጠቀሙት;	1 = አዲስ ገመድ/ክር<br>2 = የተቀቀለ ገመድ/ክር<br>3 = ሌላ T"—¨<"U ገመድ/ክር<br>4 = K?L SsÖ]Á (Clamp)<br>5 = ምንም<br>6 = አላውቅም<br>7 = ሌላ	|__|
811	ዕትብ~"/…" ለመቁረጥ U" 'u` የተጠቀሙት?	1 = ›ዲስ ምላጭ<br>2 = ማንኛውንም ምላጭ<br>3 = የተቀቀለ መቀስ<br>4. መንኛውንም መቀስ<br>5 = አላውቅም<br>6 = ሌላ	|__|
812	እትብቱ ከተsጠረና ከተቆረጠ በ🕒ላ ¾}Å[Ñuf 'Ñ` 'u`;	1 = አዎ<br>2 = አይ (ወደ 824)	|__|

**አዎ ከሆነ** ፡ ምን ነበር እትብቱ ከተቆረጠ በ🕒ላ ¾}Å[Ñuf ? ›ታ"wwLf ፤ ¾T>SKŸ}¨< G<K< LÃ UMịf ›É`		ለያንዳንዲ ጥያቄ 1 =አዎ 2 = አይ	
	813	ቅቤ	|__|
	814	አመድ	|__|
	815	ቅባት(SÉN'>f'f ¾K?K¨<)	|__|

<table>
<tr><td></td><td>816</td><td>የእንሰሳት እበት/ኩበት</td><td>|__|</td></tr>
<tr><td></td><td>817</td><td>ዘይት</td><td>|__|</td></tr>
<tr><td></td><td>818</td><td>ቀ´ቃዛ ውሃ</td><td>|__|</td></tr>
<tr><td></td><td>819</td><td>ሌላ</td><td>|__|</td></tr>
<tr><td>820</td><td>እትብቱ LÃ ì[Iªdf (antiseptic) SÉP’>f }Å`Ô ’u`?</td><td>1 = አዎ
2 = አይ (ወደ 824)
3 = አላውቅም(ወደ 824)</td><td>|__|</td></tr>
<tr><td>821</td><td>›- ŸJ’: •እንw`~ LÃ የተደረገዉ ክሎሮሃክሲዲን (chlorohexidine) ’u`?</td><td>1 = አዎ
2 = አይ(ወደ 824)
3 = አላውቅም(ወደ 824)</td><td>|__|</td></tr>
<tr><td>822</td><td>›- ŸJ’: አ”w`~ LÃ ክሎሮሃክሲዲን (chlorohexidine) ¾}Å[Ñዉ Ke”f k” ’u` <?</td><td>የቀኑ ብዛት ይጠቀስ
የማይታወቅ ከሆነ 99 ይፃፍ</td><td>|__| |__|</td></tr>
<tr><td>823</td><td>›- ŸJ’: uk” Ke”f Ñ>²? ’u` ክሎሮሃክሲዲን (chlorohexidine) ¾}Å[Ñ¨?</td><td>በቀን የተÅ[Ñuf ጊዜ ብዛት ይጠቀስ
የማይታወቅ ከሆነ 99 ይፃፍ</td><td>|__| |__|</td></tr>
<tr><td>824</td><td>(ሥም) ወዲያ¨< እንደተወለደ የአይን ጠብታ TTC ወስደዋል/ዳለች</td><td>1 = አዎ
2 = አይ
3 = አላውቅም</td><td>|__|</td></tr>
<tr><td>825</td><td>(ሥም) Ÿ}¨KÅ/‹ በምን ያህል ጊዜ ውሰጥ ነበር ገላውን/ዋ” የታጠበው/ችው;</td><td>1 = በመጀመሪያው ሰዓት
2 = ከአንድ ሰዓት በ🕒ላ (ወደ 827)
3 = ከአንድ ቀን በ🕒ላ (ወደ 828)</td><td>|__|</td></tr>
<tr><td>826</td><td>በመጀመሪያው ሰዓት ¨<eØ ከሆነ፤ ከምን ÁIM ደቂቃ በ🕒ላ ነበር;</td><td>ደቂቃውን ይጻፉ
የማ Ã.ታወቅ ከሆነ 99 ይመዝግቡ</td><td>|__| |__|</td></tr>
<tr><td>827</td><td>አንድ ሰዓት በ🕒ላ፡ ከሆነ ከምን ያህል ሰዓት በ🕒ላ ነው ይላሉ?</td><td>የሙሉ ሳአቱን ቁጥር ይመዝግቡ
መልሱ ከአንድ ሰዓት በ🕒L የሚል ከሆነ 1ን ይመዝግቡ
ከአንድ ሰዓት ተኩል በ🕒ላ የሚል ከሆነ 1ን ይመዝግቡ ፤ መልሱ ከሁለት ሰዓት ተኩል በ🕒ላ የሚል ከሆነ 2 ን ይመዝግቡ
ካልታወቀ 99ን ይመዝግቡ</td><td>|__| |__|</td></tr>
<tr><td>828</td><td>ከአንድ ቀን በ🕒ላ፡ ከሆነ፤ ከምን ያህል ቀን በ🕒ላ ነበር ይላሉ?</td><td>የሙሉ ቀን ቁጥር ይመዝግቡ
መልሱ ከአንድ ቀን በ🕒ላ የሚል ከሆነ 1ን ይመዝግቡ ከአንድ ቀን ተኩል በ🕒ላ የሚል ከሆነ 1 ን ይመዝግቡ
ካልታወቀ 99 ይመዝግቡ</td><td>|__| |__|</td></tr>
<tr><td>829</td><td>(ሥም) Ÿ}¨KÅ/‹ uኋL vK¨< በመጀመሪያው ሳምንት ¨<eØ ŸÖ<fi Ò` የአካል ንኪኪ እንዲኖረው/ራት አድርገi •ኦpòሺ¨</Áf ነበር
(k”“ KK=f” ÃÚU^M)</td><td>1 = አዎ; በቀን ውስጥ 1-7 ጊዜ
2 = አዎ; በቀን ውስጥ ከ8-12 ጊዜ
3 = አዎ; በቀን ውስጥ ከ12 ጊዜ በላይ
4 = በጭራሽ/አንድም Ñ>²?
5 = አላውቅም</td><td>|__|</td></tr>
<tr><td>830</td><td>(ሥም) በመጀመሪያው ሳምንት Ÿእ“~ ጋር ¨Ãe w‰¨<” ’u` ¾T>}—</td><td>1 = ከናት ጋር ’u` ¾T>}—¨<</td><td>|__|</td></tr>
</table>

	¨<?	2 = ሰብቻ ’u` ¾T>}—¨<<br>3 = ከሌላ ሰው ጋር ’u` ¾T>}—¨<	
831	(ሥም”) ጡት ›Øw}i¨</hf •ታ¨<mÁKi ¨Ã?	1 = አዎ<br>2 = አይ	|__|
832	(YU) Ÿ}¨KÅ/‹ K28 k“f ¨<eØ Ö<f w‰ ’u` ¾}SÑu¨</‹?	1 = አዎ (ወደ 837)<br>2 = አይ	|__|
833	›Ã ŸJ’ ŸÖ<f K?L U” ’u` ¾}cÖ¨</×f	1 = ውሃ<br>2 = ¨}f (¾እ”edf)<br>3 = pu?<br>4 = ስካር/ጉሉኮስ/Úው ÁK¨< ውሃ<br>5=ጭማቂ<br>6= ሻይ<br>7 = ሌላ	|__|
834	ሌላ (ይገለጽ)	ይገለጽ__________________________	
835	አይ ከሆነ ŸÖ<fi ¨}f K?L }ÚT] ðdi ’Ñ` SeÖf ለምን ›eðKÑ?	1 = የጡቴ ወተት በቂ አይደለም<br>2 = ቀኑን ሙሉ ከ(ሥም)ጋር ስለማልውል<br>3 = በዘመድ/ገደኛ ስለተመከርኩ<br>4 = በልምድ/ባህል eKT>Å[Ó<br>5 = ሌላ	|__|
836	ሌላ (ይገለጽ)	ይገለጽ________________________________	
837	(ሥም) Ÿ}¨KÅ/‹ uሁዋላ uU” ÁlM Ñ>²? ¨<eØ Ö<f Öv/‹;	1 = በመጀመሪያው ሰዓት<br>2 = ከአንድ ሰዓት በ🕒ላግን በምጀመሪያዉ ቀን ዉስጥ<br>3 = ከመጀመሪው ቀን በ🕒ላ	|__|
838	መጀመሪያ ŸÖ<f ¾T>¨×¨<” ¨}f (እ”Ñ`) ›Mui ›e¨ÑÉi¨<;	1 = አዎ<br>2 = አይ	|__|

839	(ሥም)እንደተወለደ በመጀመሪያው 6 ሳምንታት ውስጥ ከልምድ ›ªLÏ ውጪ በሌላ የጤና ባለሙያ ታይቶ ነበር;<br><br>ÖÁm: (YU) u}¨KÅuf/‹uf xታና Ñ>²? ¾}Å[Ñ< ¡w"u?-‹ "K ›[ÒÓØ	1 = አዎ<br>2 = አይ (¨Å 892)<br>የማይታወቅ ከሆነ 99 ይፃፍ	|__|
840	አዎ ከሆነ፤ (YU) u}¨KÅ/‹ ue”}—¨< k” ¾SËS]Á¨< ¾U`S^ ¡ffM }Å[ÑKf/Lf;	¾U`S^ ¡ffM w³f ›eÑv	|__|__|

<table>
<tr><td>841</td><td>አዎ ከሆነ፤ (YU) Ÿ}¨KÅ/‹ uሀዋL ¾SËS]Á¨< ¾U`S^ ¡ffM }Å[ÑKƒ/Lƒ ¾ƒ ’u`;</td><td>1 = u?ƒ ¨<eØ
2 = Ö?" Ÿ?L
3 = Ö?" ×u=Á
4 = Jeú•M
5 = K?L</td><td>|__|</td></tr>
<tr><td>842</td><td>አዎ ከሆነ፤ (YU) Ÿ}¨KÅ/‹ uሀዋL ¾SËS]Á¨<" ¾U`S^ ¡ffM ÁÅ[ÑKƒ/Lƒ T" ’u`;
Probe for most qualified person</td><td>1 = Êi}`
2 = ነርስ/አዋላጅ
3 = Hጤኤሠ
4 = ጤና መኮንን/ረዳት
5 = ጤልሠ
6 = ›L¨<pU/SK¾ƒ</td><td>|__|</td></tr>
<tr><td>843</td><td>(YU) Ÿ}¨KÅ/‹ uሀዋL ሁለተኛ የምርመራ ክትትል ተደርጎለት/ላት ነበር ;</td><td>1 = አዎ
2 = አይ (¨Å 855)</td><td>|__|</td></tr>
<tr><td>844</td><td>አዎ ከሆነ፤ (YU) u}¨KÅ/‹ ue"}—¨< k" ¾G<K}—¨< ¾U`S^ ¡ffM }Å[ÑKƒ/Lƒ;
.</td><td>Ÿ¨K=É uሀዋL እስከ G<K}—¨< ¾Ö?" ¡ffM ÁK<ƒ" k"ƒ ›eÑv የማይታወቅ ከሆነ 99 ይፃፍ</td><td>|__|__|</td></tr>
<tr><td>845</td><td>አዎ ከሆነ፤ (YU) Ÿ}¨KÅ/‹ uኃL ¾G<K}—¨< ¾U`S^ ¡ffM የ}Å[ÑKƒ/Lƒ ¾ƒ ’u`;</td><td>1 = u?ƒ ¨<eØ
2 = Ö?" Ÿ?L
3 = Ö?" ×u=Á
4 = Jeúታ M
5 = K?L</td><td>|__|</td></tr>
<tr><td>846</td><td>አዎ ከሆነ፤ (YU) Ÿ}¨KÅ/‹ uኃL ¾G<K}—¨<" ¾U`S^ ¡ffM ÁÅ[ÑKƒ/Lƒ T" ’u`;
Probe for most qualified person</td><td>1 = Êi}`
2 = ነርስ/አዋላጅ
3 = ጤኤሠ
4 = ጤና መኮንን/ረዳት
5 = ጤልሠ
6 = ›L¨<pU/SK¾ƒ</td><td>|__|</td></tr>
<tr><td>847</td><td>(YU) Ÿ}¨KÅ/‹ uኃL ሦስተኛ ¾U`S^ ¡ffM ተደርጎለት/ላት ነበር ;</td><td>1 = አዎ
2 = አይ (¨Å 855)</td><td>|__|</td></tr>
<tr><td>848</td><td>አዎ ከሆነ፤ (YU) u}¨KÅ/‹ ue"}—¨< k" ¾Ze}—¨< ¾U`S^ ¡ffM }Å[ÑKƒ/Lƒ;</td><td>Ÿ¨K=É uሀዋL እስከ Ze}—¨< ¾Ö?" ¡ffM ÁK<ƒ" k"ƒ ›eÑv የማይታወቅ ከሆነ 99 ይፃፍ</td><td>|__|__|</td></tr>
<tr><td>849</td><td>አዎ ከሆነ፤ (YU) Ÿ}¨KÅ/‹ uኃL Ze}—¨<" ¾U`S^ ¡ffM የ}Å[ÑKƒ/Lƒ ¾ƒ ’u`;</td><td>1 = u?ƒ ¨<eØ
2 = Ö?" Ÿ?L
3 = Ö?" ×u=Á
4 = Jeú•M
5 = K?L</td><td>|__|</td></tr>
<tr><td>850</td><td>አዎ ከሆነ፤ (YU) Ÿ}¨KÅ/‹ uኃL Ze}—¨<" ¾U`S^ ¡ffM ÁÅ[ÑKƒ/Lƒ T" ’u`;
(ባዋናነት የሚመለከተዉን ሰው አውጣጣ)</td><td>1 = Êi}`
2 = ነርስ/አዋላጅ
3 = Hጤኤሠ
4 = ጤና መኮንን/ረዳት
5 = ጤልሠ
6 = ›L¨<pU/SK¾ƒ</td><td>|__|</td></tr>
</table>

851	(YU) Ÿ}¨KÅ/‹ u¬L Ze}— ¾U`S^ ¡ffM ተደርጎለት/ላት ነበር ;	1 = አዎ<br>2 = አይ (¨Å 855)	|__|
852	(YU) u}¨KÅ/‹ ue”}—¨< k” ¾›^}—¨< ¾U`S^ ¡ffM }Å[ÑKƒ/Lƒ;	Ÿ¨K=É uሀዋL ¾›^}—¨< ¾Ö?“ ¡ffM ÁK<ƒ” k“ƒ ›eÑv<br>የማይታወቅ ከሆነ 99 ይፃፍ	|__|__|
853	(YU) Ÿ}¨KÅ/‹ u%EL ›^}—¨< ¾U`S^ ¡ffM }Å[ÑKƒ/Lƒ ¾ƒ ’u`;	1 = u?ƒ ¨<eØ<br>2 = Ö?“ Ÿ?L<br>3 = Ö?“ ×u=Á<br>4 = Jeúታ M<br>5 = K?L	|__|
854	(YU) Ÿ}¨KÅ/‹ u%EL ›^}—¨<” ¾U`S^ ¡ffM ÁÅ[ÑKƒ/Lƒ T” ’u`;	1 = Ê¡}`<br>2 = ነርስ/አዋላጅ<br>3 = ጤኤሠ<br>4 = ጤና መኮንን/ረደት<br>5 = ጤልሠ<br>6 = ›L¨<pU/SK¾ƒ አልችልም	|__|

(Kሥም) የጤና ምርመራ-ቱ u}Å[Ñ<uƒ ወቅት Ÿ²=I kØKA ¾U²[´^ተ¨< ’Ña‹ }Ÿ“¨<’¨< ’u` ¨Ã;

ጠያቂ፤ ምርጫዎቹ አንብብላት።
¾Ökc‹¨< G<K< LÃ UM¡ƒ ›É`Ó
በተጠቀሰው ጊዜ የጤና ክትትል ካልተደረገ ሰንተረዡ ውስጥ ምንም አይሞላ

	ለያንዳንዷ ጥያቄ 1 =አዎ 2 = አይ					
	(ጥይቄ #)	0-2 ቀን	(ጥያቄ #)	3-7 ቀን	(ጥየቄ #)	8-42 ቀን
ጠቅላላ ምርመራ በሕጻኑ/“E አካል ላÃ	855	|__|	856	|__|	857	|__|
ክብደት SS²”	858	|__|	859	|__|	860	|__|
የእትብት ምርመራ	861	|__|	862	|__|	863	|__|
eKጡት TØvƒ ምክር	864	|__|	865	|__|	866	|__|
Qí’</ª ጡት efÖv ማየት	867	|__|	868	|__|	869	|__|
eK •እ“ƒ“ MÏ የገላ ለጋለ ”ክኪ ጠቃሚነት መምከር	870	|__|	871	|__|	872	|__|
አደገኛ የሆኑ ምልክቶች S•` ›KT•^ተ¨<” ማየት(including sepsis)	873	|__|	874	|__|	875	|__|
eKአደገኛ መልክቶች ƒUI`ƒ መስጠት	876	|__|	877	|__|	878	|__|
ወደ (K?L) ጤና ተsም መላክ (Refer ማድረግ)	879	|__|	880	|__|	881	|__|
ሕጻን ከመንካት በፊት ስለ እጅ መታጠብ አስፈላጊነት ምክር መስጠት;	882	|__|	883	|__|	884	|__|
እትብት በንሕጽና ስለመያዝ ምክር መስጠት	885	|__|	886	|__|	887	|__|
ሕጻኑ ከተወለደ አስከ 24 ሰዓት ውሰጥ ገላውን/ዋን መታÖብ •እ”ÅK?Kuƒ/ባት ምክር መስጠት	888	|__|				

889	(KYU) u}Å[ÑKƒ/Lƒ ¾U`S^ ¡ƒƒM/¡w"u? [¡}hM ¨Ãe ›["iU;<br>**ምርጫውን አያንብቡት**	1 = ›- •[¡‰KG<<br>2 = ›Ã ›["G<U (¨Å 891)<br>3 = [¡‰KG<U ›["<G<Uም TKƒ ›M‹MU (¨Å 892)	|___|
890	›- [¡‰KG< ŸJ' ¾["iuƒ SÖ" U" ÁlM 'u`;<br><br>**U`Ý-‡" ›"wwLƒ**	1 = S<K< uS<K< [¡‰KG<<br>2 = uŸòM [¡‰KG<	|___|
891	›Ã ›["G<U ŸJ' U" ÁlM;<br><br>**U`Ý-‡" ›"wwLƒ**	1 = S<K< KS<K< ›["G<U<br>2 = uŸòM ›["G<U	|___|
892	KSÚ[h Ñ>²? u¨KÉiuƒ ¨pƒ MÏi" Kk"ƒ ¨ÃU KdU"ƒ ¨Å ¨<Ü dÃ¨×/dƒ¨× u?ƒ ¨<eØ ›qÃ}i 'u`	1 = ›-<br>2 = ›Ã (¨Å 894)	|___|
893	**›- ŸJ'** : lí'<"/"E" Ke"ƒ k"ƒ ነበር ¨Å ¨<Ü dታ¨Ü u?ƒ ¨<eØ Áq¾i¨</hƒ;	¾k•‡" w³ƒ ›eÑv<br>የማይታወቅ ከሆነ 999ይፃፍ	|___| |___| |___|
894	¾SÚ[h MÏi" u¨KÉiuƒ Ñ>²? Ÿe"ƒ k"ƒ u%EL 'u` c-‹ MÏ"/…" •ኦ"Ç=Á¿ ¾ðkÉiL†¨< (¾Ö?" vKS<Á-‹" ÚUa)	¾k•‡" w³ƒ ›eÑv<br>የማይታወቅ ከሆነ 999 ይፃፍ	|___| |___| |___|
895	¾SÚ[h MÏi" u¨KÉiuƒ Ñ>²? Ÿe"ƒ k"ƒ u%EL 'u` ""^ ue}k` K?KA‹ c-‹ MÏ"/…" ኦ"Ç='Ÿ< ¾ðkÉiL†¨< (¾Ö?" vKS<Á-‹" ÚUa):: S""ƒ c=vM T"—¨<"U ›Ã'ƒ S""ƒ	¾k•‡" w³ƒ ›eÑv<br>የማይታወቅ ከሆነ 999 ይፃፍ	|___| |___| |___|

ክፈል 9. KÚpL Qí“ƒ ስለT>Å[Ó ¡w"u?

<table>
<tr><td>900</td><td>ጠያቂ፤ ሕጻኑ/“E የተወለደው/‹¨< በኢትዮጵያ ቀንና ዘመን አቆጣ ÖC hSeŸ[U 01/2004 ¯.U ¨Ç=l SJ’<” ›[ÒÓØ

አዎ ከሆነ፤ ቃለመÖÃ ቁን ይቀጥሉ

ኣይ ከሆነ& nKSÖÃl” ሕኔርጠው በዚህ ቤተሰብ ዕድሜኣቸው Ÿ13-49 የሆኑ K?KA‹ ሴቶች "K< nKSÖÃp ÃÅ[ÓLt¨< ፡፡</td><td>1 = አዎ
2 = ኣይ(መጠይቁ ያብቃ)</td><td>|__|</td></tr>
</table>

Ÿ²=l kØKA (YU) Ÿ}¨KÅ/‹ u%EL vK<ƒ 28 k“ƒ ¨<eØ ÁÑÖS¨</Tƒ ISU "K እንዲነÓ\˜ እÖÃnKG<::

<table>
<tr><td>901</td><td>(ሥም) h}¨KÅ/‹ uG<ªL vK<ƒ መጀመሪያዎቹ 28 ቀናት ¨<eØ ታሞ/T ያውnል/ታውnለች;</td><td>1 = አዎ
2 = ኣይ</td><td>|__|</td></tr>
</table>

ለማርጋገጠ እፈልጋለሁ፡- (YU) Ÿ}¨KÅ/‹ uኃL vK<ƒ 28 k“ƒ ¨<eØ Ÿ²=l kØKA ŸU²[´^t¨< ¾ISU UM¡„‹ ¨<eØ ÁÑÖS¨</Tƒ ›K;
ምርጫዎቹን ይነበቡ።

<table>
<tr><td colspan="3">ጠያቂ፤ አዎ ለሆነው 1 ን በጠሰገባት ¨Å Ô” ይቀጥሉ፡፡

ኣይ ከሆነ 2 ብለው ወደT>kØK¨< ØÁo ÃH>Æ

U”U ¾በሽታ UM¡ƒ ŸK?K• ¨Å T>kØK¨< ¡õM 10 H>É::</td><td colspan="2">¾SÈS]Á¨< UM¡ƒ ¾ታ¾uƒ/vƒ •óÉT@ (uk” ÃÖke)</td><td colspan="2">የጤና ክትትል አድርገሽ ’u`<br>1 = ›-<br>2 = ›Ã</td><td colspan="2">›- ŸJ’ ¾ƒ ’u` ¾¨cÉi¨/hƒ;

1 = Ö?“ Ÿ?L
2 = Ö?“ ×wÁ
3 = Jeú/ታM
4 = ጤልሠ u?ƒ
5 = ¾vIM NŸ=U u?ƒ
6 = SÉP’>ƒ u?ƒ</td><td colspan="2">የጤና ክትትል አደረግሽ ŸJ’ T”” ’u` ÁTŸ`i¨<;

1 = ጤኤሠ
2 = ነርስ
3 = ጤና መኮንን
4 = ó`Tc=eƒ
5 =ሐኪም(ዳክተር)
6 = ¾vIM NŸ=U
7 = K?L</td><td colspan="2">የጤና ክትትል ካልተደረገ ለU” ;
1 = qÃ„ ÃhKªM/Lታል wÂ uTcw
2 =¾Ö?“ }sS< •\p eKJ’
3=¾I¡U“ ¨Ü Ÿõ}— uSJ’<
4=¾Ö?“ }sTƒ ን eKTLU“t¨<
5 = u?}cu? eLMðkÆ
6 = uTIu[cu< ›ªm c-‹ NŸ=U u?ƒ •እ”ÇM¨eÉ eKSŸ\˜
7 = K?L</td></tr>
<tr><td>¾Ö<ƒ õLÔƒ መቀነስ</td><td>902</td><td>|__|</td><td>903</td><td>|__| |__|</td><td>904</td><td>|__|</td><td>905</td><td>|__|</td><td>906</td><td>|__|</td><td>907</td><td>|__|</td></tr>
<tr><td>(YU) ¡wÅ~/… ´p}— ’u`;</td><td>908</td><td>|__|</td><td>909</td><td>|__| |__|</td><td>910</td><td>|__|</td><td>911</td><td>|__|</td><td>912</td><td>|__|</td><td>913</td><td>|__|</td></tr>
<tr><td>ቶሎ ቶሎ መተንፈስ ¨ÃU KS}”ðe መቸገር</td><td>914</td><td>|__|</td><td>915</td><td>|__| |__|</td><td>916</td><td>|__|</td><td>917</td><td>|__|</td><td>918</td><td>|__|</td><td>919</td><td>|__|</td></tr>
</table>

<table>
<tr><td></td><td></td><td></td><td></td><td></td><td></td><td></td><td></td><td></td><td></td><td></td><td></td><td></td></tr>
<tr><td>¾Å[ƒ ¨Å ¨<eØ Sc`ÔÉ
(Chest in-drawing)</td><td>920</td><td>|__|</td><td>921</td><td>|__||__|</td><td>922</td><td>|__|</td><td>923</td><td>|__|</td><td>924</td><td>|__|</td><td>925</td><td>|__|</td></tr>
<tr><td>ባልተለመደ ሁኔታ መቀዝቀዝ ወይንም መሞቅ</td><td>926</td><td>|__|</td><td>927</td><td>|__||__|</td><td>928</td><td>|__|</td><td>929</td><td>|__|</td><td>930</td><td>|__|</td><td>931</td><td>|__|</td></tr>
<tr><td>ባልተለመደ ሁኔታ ንቁ አለመሆን</td><td>932</td><td>|__|</td><td>933</td><td>|__||__|</td><td>934</td><td>|__|</td><td>935</td><td>|__|</td><td>936</td><td>|__|</td><td>937</td><td>|__|</td></tr>
<tr><td>¾SÇö‹ u=Ý SJ”
(Yellow palms/soles/eyes)</td><td>938</td><td>|__|</td><td>939</td><td>|__||__|</td><td>940</td><td>|__|</td><td>941</td><td>|__|</td><td>942</td><td>|__|</td><td>943</td><td>|__|</td></tr>
<tr><td>ተቅማጥ ነበረው/ት</td><td>944</td><td>|__|</td><td>945</td><td>|__||__|</td><td>946</td><td>|__|</td><td>947</td><td>|__|</td><td>948</td><td>|__|</td><td>949</td><td>|__|</td></tr>
<tr><td>Convulsions/ማቀጥቀጥ</td><td>950</td><td>|__|</td><td>951</td><td>|__||__|</td><td>952</td><td>|__|</td><td>953</td><td>|__|</td><td>954</td><td>|__|</td><td>955</td><td>|__|</td></tr>
<tr><td>Skin pustules ¾qÇLÃ iõ ’u` ¨Ã</td><td>956</td><td>|__|</td><td>957</td><td>|__||__|</td><td>958</td><td>|__|</td><td>959</td><td>|__|</td><td>960</td><td>|__|</td><td>961</td><td>|__|</td></tr>
<tr><td>እንብርት አካባቢ ያለው ቆዳ መቅላት/የእንብርት አካባቢ ፈሳሽ</td><td>962</td><td>|__|</td><td>963</td><td>|__||__|</td><td>964</td><td>|__|</td><td>965</td><td>|__|</td><td>966</td><td>|__|</td><td>967</td><td>|__|</td></tr>
<tr><td>() K?L</td><td>968</td><td>|__|</td><td>969</td><td>|__||__|</td><td>970</td><td>|__|</td><td>971</td><td>|__|</td><td>972</td><td>|__|</td><td>973</td><td>|__|</td></tr>
<tr><td>Specify
__________</td><td colspan="12"></td></tr>
</table>

<table>
<tr><td colspan="4">ጠያቂ፡ ከ974-983 ያሉት ጥያቄዎች የሚሞሉት ከዚህ በላይ ባለዉ ሰንጠረዥ ዉስጥ እናትየዋ ለአንድ ወይም ከዚ በላይ የህመም ምልክት የጤና ክትትል አድርጋ ከነበረ ብቻ ነው</td></tr>
<tr><td>974</td><td>ከላይ የተዘረዘሩት ማንኛውም ከተዘረዘሩት ምልክቶች ከታየባት/ከታየበት የሚቀጥሉት ጥያቄ ወች የጠይቁ

የመጀመሪያዉ የህመም ምልክት ከታየ Ÿስንት ቀን በ🕒ላ ነው NŸ=U ÁTŸ`i¨<?</td><td>ሕመሙ ከጀመረበት ቀን ጅምሮ NŸ=U እe"TŸ[‹uƒ ያለውን የቀን w³ƒ ይመዝግቡ; ¾ሕመሙ UM¡ƒ uታ¾uƒ የመጀመሪያ ቀን ከሆነ 0 ብለው ይጻፉ; የሕክምና S´Ñw ("`É) "K S[Í¨<” Á[ÒÓÖ<
ምንም አየነት ህክምና ካልተደረገ 99 ይጻፍ</td><td>|__|__|</td></tr>
<tr><td>975</td><td>(YU) ŸvÉ ISU }Ñ˜„uƒ/vƒ Á¨<nM ?

ÖÁm:- ¾ŸvÉ ISU UMi„‹ Ã²`´\Lƒ</td><td>1 = አዎ
2 = አይ</td><td>|__|</td></tr>
<tr><td>976</td><td>(YU) KISS< SÉP’>ƒ ታµKƒ/Lƒ ’u` ¨Ã?</td><td>1 = አዎ
2 = አይ</td><td>|__|</td></tr>
<tr><td>977</td><td>(YU) KISS< K7 }Ÿታታይ k“ƒ uS`ô ¾T>¨cÉ Ë”ታTÃc=” (Gentamycin) ¾}vK SÉP’>ƒ ታµLƒ/Lƒ ’u` ¨Ã?

ጠያቂ፡- Ë”ታTÃc=” (Gentamycin) ¾}vKዉን SÉP’>ƒ ናሙና ወይም ፎቶ አሳይ</td><td>1 = አዎ
2 = አይ</td><td>|__|</td></tr>
<tr><td>978</td><td>(YU) KISS< K7 }Ÿታታይ k“ƒ ¾T>¨cÉ ›V¡c=K=” (Amoxicillin) ¾}vK SÉP’>ƒ ታµለት/Lት ’u` ¨Ã;</td><td>1 = አዎ
2 = አይ</td><td>|__|</td></tr>
</table>

	**ÖÁm:- ›V¡c=K=” (Amoxicillin) u¨<H }uØwÙ ¾T>cØ Ÿ=’>” SJ’<” ›e[Çƒ::**		
979	(ሥም) ¾ሕመም UM¡~ u’u[uf/vf በማንኛውም ወቅት መድ🕒ኒት ወስዶ/ወስዳ ነበር;	1 = አዎ<br>2 = አይ	|__|
980	(ሥም) ¾ሕመም UM¡~ u’u[uf/vf በማንኛውም ወቅት expressed breast milk? (በእቃ የተቀመጠ ታልቦ የጡት ወተት) ወስዶ/ወስዳ ነበር;	1 = አዎ<br>2 = አይ	|__|
981	(YU) uታSSuf/‹uf ¨pf u}cÖ¨</×f l¡U“ እ[¡}hM ¨Ãe ›M["iU;<br><br>**U`Ý-‡” ›ታ”wwLƒ**	1 = ›- እ[¡‰KG<<br>2 = ›Ã ›["G<U (¨Å 983)<br>3 = [¡‰KG<U ›["G<UU TKƒ ›M‹MU (¨Å ¡õM 10)	|___|
982	**›- ከሆነ ፡** እ[¡‰KG< ŸJ’ ¾እ[Ÿታሽ SÖ” U” ÁlM ’u`;<br><br>U`Ý-‡” ›”wwLƒ	1 = S<K< uS<K< [¡‰KG<<br>2 = uŸòM [¡‰KG<	|___|
983	**›Ã ŸJ’:** ›M["G<U ŸJ’ U” ÁlM;<br>**U`Ý-‡” ›”wwLƒ**	1 = S<K< KS<K< ›["G<U (¨Å ¡ðM 10)<br>2 = uŸòM ›["G<U (¨Å ¡ðM 10)	|___|

ክፍል 10. ulÃ¨ƒ eKK?K< ÚpL Qí“ƒ (0-28 k“ƒ ¨<cØ ¾V~)

ÖÁm:- ከ28 ከናት በፊት የህጻኑ ህይወት ካለፈ ወይም (YU) uህይ>¨ƒ ŸK?K/‹ Ÿ²=l uታ‹ uT>Ñ–<ƒን ØÁo-‹ eKISS</TE &eK}Å[ÑKƒ/Lƒ l¡U“ ⊡⊡እ”Ç=G<U KVƒ eLun¨</ƒ Ui”Áƒ ÖÃp::

ስለ ሞቱ ህጻናት ማዉራት በጣም ከባድ እንደሆነ እረዳለው ፡፡ ስለዚህ ጥያቄዎቹን ለመመለስ ጭማሪ ጊዜ ከስፈለገ አሳውቂኝ፡፡ ይሄ መረጃ በማም አስፈላጊ ነው ፤ ምክንያቱም መረጃዉ መንግስት የህፃናት ጤንነትን ለማሻሻል የሚያረገውን ጥረት ይረዳል/ያግዛል፡፡

((YU) M¡ ŸSV~/… uòƒ ŸT>Ÿ}KK<ƒ ¨<eØ ¾ƒ™‡ ¾lSU UM¡„‹ •ይታዩuf/vf ’u`;	1 =አዎ 2 = አይ		
	1000		|___|

**¾T>SKŸ}¨< G<K< LÃ UM¡ƒ ›É`Ó::**		S}”ðe ›KS‰M ¨ÃU „KA „KA S}”ðe	
	1001	¾Å[ƒ Sc`ÔÉ	|___|
	1002	vM}KSÅ G<’@ታ SVp ¨ÃU Sk´k´	|___|
	1003	vM}KSÅ G<’@ታ ”l ›KSJ”	|___|
	1004	¾SÇõ ¨ÃU ¾›Ã” u=Ý SJ”	|___|
	1005	}pTØ	|___|
	1006	ማንቀጥቀጥ (Convulsions)	|___|
	1007	እጅ ፤ እገር ቢጫ መሆን	|___|
	1008	¾qÇLÃ iõታ• (Skin pustules)	|___|
	1009	¾›”w`ƒ SpLƒ ¨ÃU ðdi ’Ñ` S•`	|___|
	1010	K?L (ÃÑKî)	|___|

1011	K?L (ÃÑKî)	ÃÑKî	|___|
1012	(ሥም) በህመም ምክንÁት ሕይወቱ/... ካለፈ በዚያ ጊዜ ¾l¡U“ ክትትል ተደርጎለት/ላት ነበር;	1 = አዎ<br>2 = አይ(መጠይቀን ጨርስ)	
1013	አዎ ከሆነ ያንን ክትትል ያደረገለት ማን ነበር ;<br><br>ካልታወቀ 9 ይሞላ	1 = ጤልሠ<br>2 = ጤኤሠ<br>3 = ነርስ<br>4 = ሐኪም<br>5 = ጤና መኮንን<br>5 = የፋርማሲ ባለሙያ<br>6 = ሌላ	|__|
1014	(YU) ሀየ>¨~/... ŸTKñ uòƒ ¾l¡U“ ¡ffM }Å`ÔKƒ/Lƒ ŸJ’ Kሀይ>¨~/... TKõ U¡”Áƒ J• ¾}Ñ–¨< U”É’¨< (uÖ?“ vKS<Á ¾}’Ñ^ƒ”) ;	1 = ¾d”v U‹<br>2 = ተቅማጥ<br>3 = ŸvÉ ISU (Sepsis)<br>4 = ¾S}”ðe ‹Ó`<br>5= Ÿ¨p~ uòƒ uS¨KÉ (Pre erm)<br>6 = K?L (ÃÑKî)	
1015	K?L ካK (ÃÑKî)	(ÃÑKî)______________________	

ÖÁm:- ለዚከ ቤተሰብ የሚደረገዉን መጠይቅ ከመጨረስህ በፊት ¾T>Ÿ}K<ƒ” G<’@ታ-‹ uT>Ñv ›[ÒÓØ&

1. በዚህ ቤተሰብ ውስጥ ዕድሜያቸው ከ13-49 J’¨< መጠይቅ ያልተደረገላቸው ሴቶች አሉ;
2. በዚህ ቤተሰብ ውስጥ ከSeŸ[U 01/2004 ¨Ç=l ነፍሰ ጡር ሆነው መጠይቅ ያልተደረገላቸው c?„‹ አሉ;
(3) በዚህ ቤተሰብ ውስጥ ከSeŸ[U 01/2004 ¨Ç=l ወልደው መጥይቅ ያልተደረገላቸው c?„‹አሉ;
(4) በቅርብ ወልደው ÚpL lí“ƒ ÁL†¨<“ መጠይቁ ÁM}Å[ÑL†¨< c?„‹ አሉ;

ŸLÃ K}²[²\ƒ ØÁo-‹ SMc< አዎ ከሆነ፤ ተገቢዉ nKSÖÃp SÅ[Ñን< ›[ÒÓØ::

ŸLÃ K}²[²\ƒ ØÁo-‹ G<K< SMc< አይ ከሆነ፤ መÖÃቁን አጠናቅ፡፡ ለጊዜአቸውም ምስጋና አቅርብ፡፡
