# Supplementary material for: Coverage of antenatal, intrapartum, and newborn care in 104 districts of Ethiopia: A before and after study four years after the launch of the national Community-Based Newborn Care programme
Source: PLoS One. 2021 Aug 5;16(8):e0251706. doi: 10.1371/journal.pone.0251706 (PMC8341496; doi:10.1371/journal.pone.0251706)
Supplement: S1 Table — (PDF) [file pone.0251706.s005.pdf]

S1 Table. Description of the Community-Based Newborn Care (CBNC) programme detailing the implementation activities adapted from Template for Intervention Description and Replication (TIDieR) framework

Activities	Activity type	Target audience	Where and how much	Facilitators	What was the purpose, material and procedure used
**A. Training**					
1. Cascaded training of trainers	Onetime activity	Staff from federal ministry of health regional health bureaus, zonal health departments and health facility staff	Four-day training at national, regional, and zonal levels	Ministry of health and implementing partners	**Purpose:** to create a pool of trainers for each level of the health system.<br><br>**Materials:** facilitator's guide which provided instruction and content of training activities, instructional videos, participants manual, chart booklet (to assist with assessment, classification and treatment), CBNC registers, drugs (gentamicin, amoxicillin and chlorhexidine), syringe with needle, gloves, clinical thermometer, weighing scale, timer for counting breaths, family health guide[a], ambu bag, mannequin and wall chart.<br><br>**Procedure:** following the facilitator's guide, a face-to-face training on CBNC programme components.
2. Health centre staff training	Onetime activity	Health center staff with integrated management of newborn and childhood illnesses training	Two-day training at the zonal level	Zone health department staff	**Purpose:** to strengthen the referral linkages between health centres and satellite health posts, and train health centre staff so they can improve the quality of the support provided to health extension workers.<br><br>**Materials:** facilitator's guide which provided instruction and content of training activities, instructional videos, participants manual, chart booklet (to assist with assessment, classification and treatment), CBNC registers, drugs (gentamicin, amoxicillin and chlorhexidine), syringe with needle, gloves, clinical thermometer, weighing scale, timer for counting breaths, family health guide[a], ambu bag, mannequin and wall chart.<br><br>**Procedure:** following the facilitator's guide, a face-to-face training on CBNC programme components.
3. Health extension worker training	Onetime activity	Health extension workers	Four-day training at the zonal level	Zone health department staff	**Purpose:** to train HEWs to provide CBNC related services, mobilize community to seek care for newborn illnesses and work with the woman development army leaders to generate demand for maternal and newborn health services.<br><br>**Materials:** facilitator's guide which provided instruction and content of training activities, instructional videos, participants manual, chart booklet (to assist with assessment, classification and treatment), CBNC registers, drugs (gentamicin, amoxicillin and chlorhexidine), syringe with needle, gloves, clinical thermometer, weighing scale, timer for counting breaths, family health guide[a], ambu bag, mannequin and wall chart.<br><br>**Procedure:** following the facilitator's guide a skill-based training with both classroom sessions and intensive practical exercises.

Activities	Activity type	Target audience	Where and how much	Facilitators	What was the purpose, material and procedure used
4. Woman development army leaders' orientation	Continuous activity	Woman development army leaders	Continuous kebele level orientation	Health extension workers	**Purpose:** to provide orientation to woman development army leaders on how to provide counselling on promotive and preventive health behaviours, identify pregnant women and newborns, educate and identify pregnancy and newborn danger signs, promote health-seeking behaviour for pregnant women and newborns and encourage completion of treatment for sick young infants.<br><br>**Materials:** family health guide[a].<br><br>**Procedure:** through continuous meetings between health extension workers and woman development army leaders.
**B. Supervision, support, and mentorship**					
1. Post training follow-up visit	Onetime activity	Health extension workers	At health posts within 4-6 weeks post the initial CBNC training	Staff from district health office, health center, implementing partners, and an individual who facilitated the training of trainers	**Purpose:** to ensure newborn sepsis management skills (assessment, classification, treatment and registration) provided during the CBNC programme training were being applied, to note any challenges to implementation, and to collect data on any possible serious bacterial infection cases that had already been managed.<br><br>**Materials:** N/A<br><br>**Procedure:** face-to-face meeting with health extension workers.
2. Performance review and clinical mentoring meeting	Biannual	Health extension workers	Two-day meeting at the district level	Health center staff and implementing partners	**Purpose:** to assess the heath extension workers' performance on delivering service for young infants by reviewing CBNC registers, to provide clinical mentorship on the assessment and treatment of young infants and to identify bottlenecks to service provision.<br><br>**Materials:** a guideline for conducing performance review and clinical mentoring meeting.<br><br>**Procedure:** health center staff reviewed pregnant, newborn and CBNC registers brought by 10-20 health extension workers. For CBNC, they assessed if health extension workers classified a newborn correctly given the registered set of symptoms and, given the recorded classification, if they provided the appropriate treatment. Based on gaps identified from the register, health centre staff provided clinical training and mentorship. A visit to a household with a newborn was conducted together.
3. Program specific supportive supervision	Monthly	Health extension workers	Monthly supportive supervision at health posts	Health center staff	**Purpose:** to improve programme management, assess skill levels, and determine the number of newborns managed at health facilities.<br><br>**Materials:** standard supervision checklist.<br><br>**Procedure:** health center staff reviewed health extension workers activities for CBNC services and provided immediate feedback and continuous training.

[a] Family health guide is low literacy pictorial tool for behaviour change communication
