# Supplementary material for: Coverage of antenatal, intrapartum, and newborn care in 104 districts of Ethiopia: A before and after study four years after the launch of the national Community-Based Newborn Care programme
Source: PLoS One. 2021 Aug 5;16(8):e0251706. doi: 10.1371/journal.pone.0251706 (PMC8341496; doi:10.1371/journal.pone.0251706)
Supplement: S2 Table — (PDF) [file pone.0251706.s006.pdf]

S2 Table. Key components of the CBNC programme training for health extension workers

**1. Pregnancy identification and antenatal care**<br>- Introduction of pregnancy identification<br>- Registering pregnant women and calculating expected due date<br>- Focused antenatal care<br>- Barriers to antenatal care<br>- Home care during pregnancy<br>- Use of Family Health Guide[a]	**2. Birth preparedness and complication readiness**<br>- Counselling skills for birth planning<br>- Using Family Health Guide[a] for counselling
**3. Delivery**<br>- Safe and clean delivery<br>- Promotion of skilled delivery<br>- Barriers to skilled delivery<br>- Danger signs during labour and delivery	**4. Essential newborn care**<br>- Brief introduction of essential newborn care<br>- Steps of immediate newborn care using a mannequin.
**5. Postpartum care**<br>- Content and timing of postnatal care home visit<br>- Hand washing, measuring temperature and weight and counselling skills during routine postnatal care home visit<br>- Maternal danger signs in the postpartum period<br>- Barriers to and solutions for postnatal care home visit<br>- Practice filling out the Integrated Maternal and Child Care Card and family folder	**6. Management of the sick young infant**<br>- Management of birth asphyxia preterm and/or low birth weight babies<br>- Assessment and classification of very severe disease, local bacterial infection, jaundice diarrhoea and HIV infection in young infants
**7. Assessing young infants' weight and feeding**<br>- How to read a weight for age chart<br>- How to check for feeding problems and assess breastfeeding<br>- Signs of good attachment for breastfeeding<br>- How to teach correct positioning and attachment for breastfeeding<br>- Assessing young infants for underweight and feeding problems<br>- Assessing young infants for immunization status	**8. Identification, treatment and counselling of the mother and following up on sick young infants**<br>- Antenatal and postnatal care home visits<br>- Actions to be taken to identify treatment<br>- Steps of newborn resuscitation<br>- Immunizing the sick young infant<br>- Expressing and feeding the preterm infant with breast milk
**9. When referral is not possible**<br>- Amoxicillin and gentamycin dosage<br>- Calculating correct doses of amoxicillin and gentamycin<br>- Demonstration of giving gentamycin injection<br>- Practice of giving gentamycin injection<br>- Counselling and negotiation skills during postnatal home visit to sick young infant	**10 Care of the sick young infant**<br>- Providing follow-up care to complete treatment for very severe disease<br>- Negotiation skills with the caretaker<br>- Filling out the young infant register

[a] Family health guide is low literacy pictorial tool for behaviour change communication
