# Supplementary material for: Coverage of antenatal, intrapartum, and newborn care in 104 districts of Ethiopia: A before and after study four years after the launch of the national Community-Based Newborn Care programme
Source: PLoS One. 2021 Aug 5;16(8):e0251706. doi: 10.1371/journal.pone.0251706 (PMC8341496; doi:10.1371/journal.pone.0251706)
Supplement: S3 Table — (PDF) [file pone.0251706.s007.pdf]

S3 Table. Antenatal care and institutional delivery for mothers who had a birth in the 3-15 months prior to the baseline (October –December 2013) and follow-up (November-December 2017) surveys.

	OR	P-value	AOR* (95%CI)	P-value
**1. Early identification of pregnancy**				
One or more antenatal care visit	2.30 (1.78,3.0)	<0.0001	2.25 (1.73,2.92)	<0.0001
Informed woman development army leader of pregnancy	0.74 (0.34,1.62)	0.46	0.61 (0.28,1.31)	0.20
**2. Provision of focused antenatal care**				
4 or more antenatal care visits	2.03 (1.67,2.48)	<0.0001	2.01 (1.64,2.48)	<0.0001
Among those having any antenatal care visit				
First antenatal care visit at a health centre	1.22 (0.95,1.57)	0.12	1.23 (0.95,1.59)	0.12
Advised on birth preparedness plan	0.76 (0.60,0.97)	0.03	0.76 (0.60,0.98)	0.03
Informed about pregnancy danger signs	0.87 (0.68,1.11)	0.25	0.89 (0.69,1.14)	0.35
Informed about breastfeeding	1.07 (0.83,1.39)	0.58	1.15 (0.88,1.49)	0.30
Received information on nutrition	0.62 (0.49,0.80)	<0.001	0.65 (0.50,0.85)	<0.001
Weight measured	1.00 (0.74,1.35)	0.99	1.01 (0.75,1.37)	0.92
Height measured	1.15 (1.04,1.26)	0.09	1.15 (1.04,1.27)	0.01
Blood pressure measured	0.84 (0.65, 1.07)	0.16	0.84 (0.66,1.08)	0.18
Gave urine sample for a test	2.04 (1.57,2.65)	<0.0001	2.06 (1.58,2.69)	<0.0001
Gave blood sample for syphilis test	1.62 (1.21,2.17)	<0.0001	1.65 (1.21,2.25)	<0.0001
Received iron folate tablets or iron syrup	1.52 (1.17,1.99)	<0.01	1.54 (1.17,2.01)	<0.01
Received HIV testing	0.44 (0.34,0.56)	<0.0001	0.43 (0.33,0.55)	<0.0001
**3. Promotion of institutional delivery**				
Hospital or health centre delivery	5.62 (4.47,7.09)	<0.0001	5.52 (4.34,7.01)	<0.0001

*Adjusted for maternal age and education.
