# Supplementary material for: Coverage of antenatal, intrapartum, and newborn care in 104 districts of Ethiopia: A before and after study four years after the launch of the national Community-Based Newborn Care programme
Source: PLoS One. 2021 Aug 5;16(8):e0251706. doi: 10.1371/journal.pone.0251706 (PMC8341496; doi:10.1371/journal.pone.0251706)
Supplement: S4 Table — (PDF) [file pone.0251706.s008.pdf]

S4 Table. Clean and safe delivery, immediate newborn care and complications management for mothers who had a birth in the 3-15 months prior to the baseline (October – December 2013) and follow-up (November-December 2017) surveys

	**Home deliveries**				**Institutional deliveries**			
	**OR**	**P-value**	**AOR***	**P-value**	**OR**	**P-value**	**AOR***	**P-value**
**4. Clean and safe Delivery**								
Birth assistant washed hands with soap [a]	0.79 (0.60,1.03)	0.09	0.80 (0.60,1.05)	0.12	0.54 (0.39,0.75)	<0.001	0.54 (0.39,0.76)	<0.001
Birth assistant wore gloves [b]	1.24 (0.85,1.82)	0.26	1.26 (0.84,1.87)	0.26	0.39 (0.17,0.90)	0.02	0.37 (0.15,0.91)	0.03
Delivery took place on a clean surface[c]	0.26 (0.18,0.38)	<0.0001	0.25 (0.17,0.37)	<0.0001	0.73 (0.33,1.66)	0.45	0.69 (0.30,1.61)	0.39
**5. Immediate newborn care**								
Clamp or new or boiled thread or string used to tie cord	1.23 (0.91,1.69)	0.17	1.30 (0.94,1.77)	0.10	1.04 (0.71,1.53)	0.82	1.09 (0.73,1.63)	0.66
New razor blade or sterilized scissors used to cut cord	0.50 (0.32,0.79)	<0.01	0.49 (0.32,0.77)	<0.01	0.76 (0.52,1.09)	0.13	0.78 (0.53,1.13)	0.18
Antiseptic used on the cord	6.98 (2.34,20.81)	<0.0001	7.17 (2.39,21.55)	<0.0001	3.75 (1.76,8.02)	<0.001	3.66 (1.73,7.74)	<0.001
Newborn postnatal check in first 2 days	0.21 (0.09,0.50)	<0.0001	0.20 (0.08,0.48)	<0.0001	0.15 (0.08,0.29)	<0.0001	0.15 (0.08,0.29)	<0.0001
**6. Recognition and management of asphyxia**								
Newborn with difficulty crying/breathing	0.31 (0.17,0.58)	<0.0001	0.30 (0.16,0.56)	<0.0001	0.43 (0.24,0.71)	<0.001	0.43 (0.26,0.70)	<0.001
Among them, resuscitated newborns	2.48 (0.40,15.21)	0.31	1.72 (0.23,12.67)	0.59	0.58 (0.24,1.38)	0.21	0.52 (0.17,1.55)	0.23
7. Prevention and management of hypothermia								
Newborn placed on mother's belly or chest immediate after birth	2.06 (1.48,2.88)	<0.0001	2.06 (1.47,2.88)	<0.0001	1.49 (1.00,2.22)	0.05	1.54 (1.02,2.32)	0.04
Bathing of newborn delayed for 24 hrs	1.79 (1.30,2.47)	<0.001	1.79 (1.29,2.49)	<0.001	1.21 (0.82,1.79)	0.34	1.25 (0.84,1.86)	0.26
**8. Management of pre-term and low birth weight neonates**								
Newborn weighed at birth	1.43 (0.89,2.31)	0.13	1.39 (0.86,2.25)	0.18	0.94 (0.67,1.32)	0.73	0.97 (0.69,1.36)	0.84

*Adjusted for maternal age and education.
