# Supplementary material for: Coverage of antenatal, intrapartum, and newborn care in 104 districts of Ethiopia: A before and after study four years after the launch of the national Community-Based Newborn Care programme
Source: PLoS One. 2021 Aug 5;16(8):e0251706. doi: 10.1371/journal.pone.0251706 (PMC8341496; doi:10.1371/journal.pone.0251706)
Supplement: S5 Table — (PDF) [file pone.0251706.s009.pdf]

S5 Table. Treatment of sick young infants reported by mothers who had a birth in the 3-15 months prior to the baseline (October –December 2013) and follow-up (November-December 2017) surveys.

	**OR**	**P-value**	**AOR***	**P-value**
**Management of possible serious bacterial infection at community level**				
Children sick in the first 59 days of life	0.88 (0.64,120)	0.41	0.86 (0.63,1.18)	0.35
Symptoms of possible serious bacterial infection[c]	0.92 (0.66,1.30)	0.65	0.91 (0.64,1.29)	0.61
Among young infants with possible serious bacterial infection				
Amoxicillin for 7 days	9.2 (4.52,18.85)	<0.0001	12.17 (5.47,27.07)	<0.0001
Gentamicin for 7 days	3.74 (1.37,10.18)	0.01	3.96 (1.37,11.46)	0.01
Amoxicillin and gentamicin and for 7 days	5.40 (1.45,20.09)	0.01	4.90 (1.18,20.23)	0.03

*Adjusted for maternal age and education.
